# Anticancer activity of a novel methylated analogue of *L-*mimosine against an in vitro model of human malignant melanoma

**DOI:** 10.1007/s10637-019-00809-0

**Published:** 2019-06-26

**Authors:** Sotiris Kyriakou, Melina Mitsiogianni, Theodora Mantso, William Cheung, Stephen Todryk, Stephany Veuger, Aglaia Pappa, David Tetard, Mihalis I. Panayiotidis

**Affiliations:** 1 Department of Applied Sciences, Northumbria University, Newcastle Upon Tyne, UK; 2 Department of Molecular Biology & Genetics, Democritus University of Thrace, Alexandroupolis, Greece

**Keywords:** Metal chelators, *L-*mimosine analogues, Skin cancer, Anticancer activity, Melanoma

## Abstract

The anticancer activity of a series of novel synthesized, hydroxypyridone-based metal chelators (analogues of *L-*mimosine) was evaluated in an in vitro model of melanoma consisting of malignant melanoma (A375), non-melanoma epidermoid carcinoma (A431) and immortalized non-malignant keratinocyte (HaCaT) cells. More specifically, we have demonstrated that the *L-*enantiomer of a methylated analogue of *L-*mimosine (compound 22) can exert a potent anticancer effect in A375 cells when compared to either A431 or HaCaT cells. Moreover, we have demonstrated that this analogue has the ability to i) promote increased generation of reactive oxygen species (ROS), ii) activate both intrinsic and extrinsic apoptosis and iii) induce perturbations in cell cycle growth arrest. Our data highlights the potential of compound 22 to act as a promising therapeutic agent against an in vitro model of human malignant melanoma.

**Electronic supplementary material:**

The online version of this article (10.1007/s10637-019-00809-0) contains supplementary material, which is available to authorized users.

## Introduction

Skin cancer is categorized into three main types based on their cellular localization and clinical behavior including basal and squamous cell carcinoma as well as melanoma [1, 2]. In particular, malignant melanoma is a tumor arising in melanocytes and once in the progressive stage it can invade and penetrate beyond the dermis layer [3]. Metastasis occurs in the lymph nodes and/or distant sites like lungs, liver and nervous system and therefore development of clinically effective therapeutic strategies is of utmost importance [4]. Despite the fact that transition metals have a significant importance in maintaining health, they can also contribute into the development and progression of many types of cancer. For example, elevated copper levels can promote tumor development, angiogenesis and metastasis while other studies have shown the importance of such elevation in the formation of soluble extracellular E-cadhenin fragment (sE-CAD) that is implicated in cancer cell invasion [5–8]. In the case of iron, reactive oxygen species (ROS) are formed and shown to be involved in carcinogenesis by various means including overexpression of proteins participating in angiogenesis and metastasis (e.g. SNAIL, AP-1 and VEGF), activation of the oncogenic NF-κB pathway and others [9–13]. Finally, zinc overload, can lead to overexpression of zinc-depended enzymes like matrix metalloproteinases (MMPs) that can degrade components of the extracellular matrix (ECM) thus further contributing to angiogenesis and metastasis [14, 15].

**Fig. 1:**
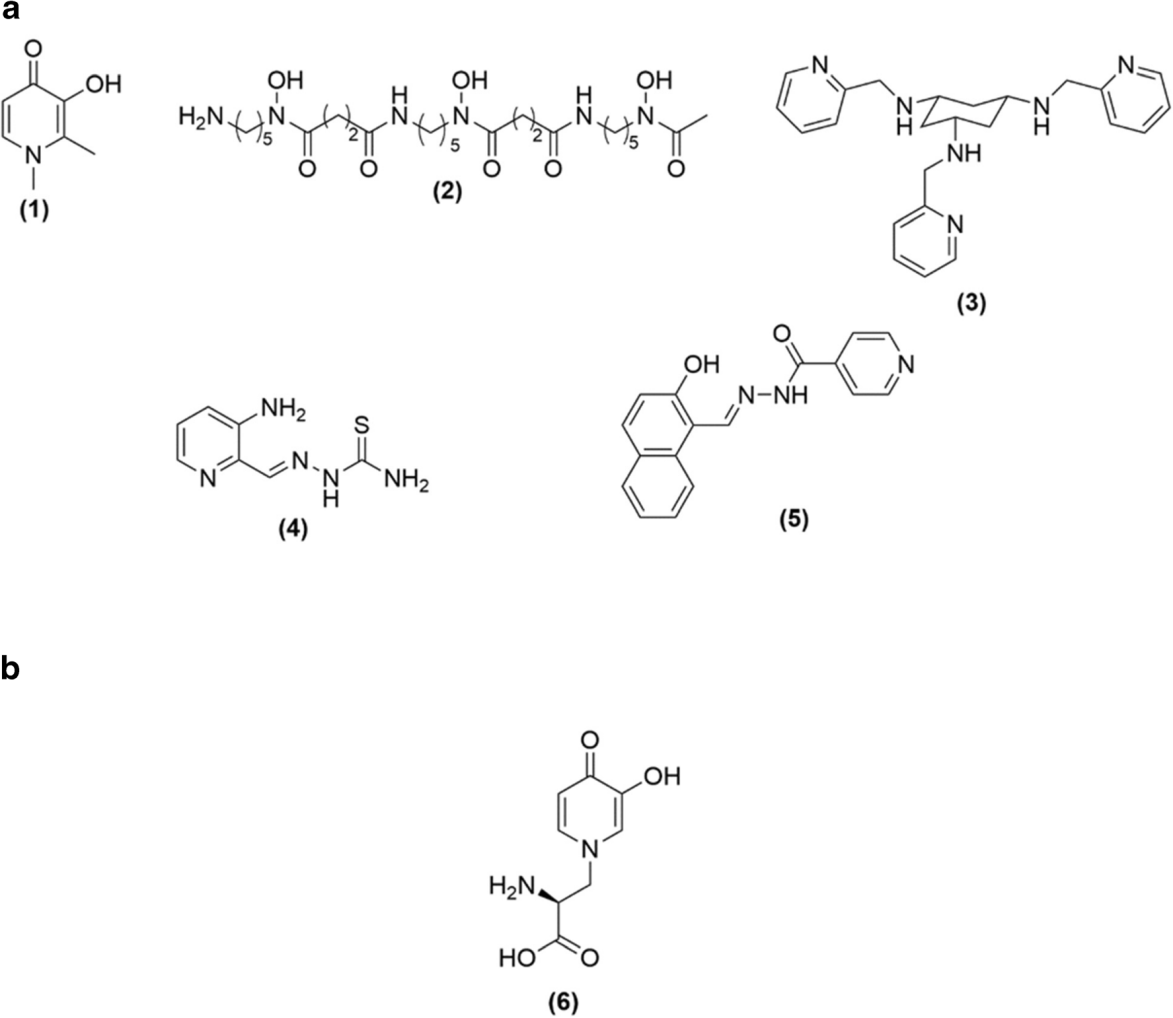
**a** Structural representation of selective metal chelators with anti-cancer properties. Deferiprone (DFP) (**1**), Desferrioxamine (DFO) (**2**), Tachpyridine (**3**), Triapine (**4**) and 2-hydroxy-1-naphthyl aldehyde isonocitinyl hydrazone (**5**); **b** Structure of L-mimosine (**6**)

For this reason, it is not surprising that metal chelators are being considered as anticancer agents by restoring metal homeostasis and consequently inhibiting i) cell growth, ii) intracellular ROS formation and iii) cell proliferation [16, 17]. As a result, a variety of them have been synthesized (e.g. deferiprone; DFP [18], desferrioxamine; DFO [19], tachpyridine [20], triapine [21] and 2-hydroxy-1-naphthyl aldehyde isonocitinyl hydrazine [22]) all of which are capable of acting as anticancer agents (Fig. 1a). However, the difficulty associated with their development is the risk for potential side-effects due to their ability to interact with various metalloproteins. For example, DFP used in the treatment of iron overload is known to inhibit lipoxygenase and tyrosine hydroxylase and is also associated with agranulocytosis [23–25].

*L*-mimosine is a natural 3-hydroxy-4(1*H*)pyridinone (3,4-HOPO) iron chelator containing an amino acid side chain (Fig. 1b) that is derived from the plants *Mimosa* and *Leucaena genara* endowed with a range of bioactivities including anti-cancer, anti-inflammatory, anti-viral, anti-fibrotic, etc. [26, 27]. Previously, *L*-mimosine was shown to i) inhibit tyrosinase and thyroxine decarboxylases, ii) induce cell death and cell cycle growth arrest, iii) suppress elongation of DNA replication, iv) disrupt deoxyribonucleotide metabolism and v) inhibit the transcription of serine hydroxymethyltransferase [28–39]. Finally, *L-*mimosine has been shown to exert anticancer activity against various melanoma cell lines and although initially appeared as a potentially attractive anticancer agent, its marked cytotoxic side-effects have discouraged its further development [40–42].

To the best of our knowledge, there are no reports on how *L*-mimosine can enter cells. However, being a close analogue of *L*-DOPA, it may be that it acts as a substrate of the large neutral amino acids’ transporter namely LAT-1 which is known to possess a wide substrate specificity including *L*-DOPA [43–46]. Interestingly, LAT-1 is overexpressed in various cancer cells thus providing an opportunity to specifically target them [47]. To combat the side-effects of *L*-mimosine it is, however, necessary to identify safer analogues. In particular, the 3,4-HOPO chelating moiety can be replaced by relevant isomers such as 1-hydroxy-2(1*H*)- pyridinone (1,2-HOPO) and 3-hydroxy-2(1*H*)-pyridinone (3,2-HOPO) or the less powerful coordinating group 3-hydroxy-4-pyranone. As the ligand binding pocket of LAT-1 has hydrophobic domains provided by residues F252, F402 and V148, *L*-mimosine can also be made more lipophilic to help target the LAT-1 transport mechanism [46].

The aim of the current study was to i) design and synthesize a series of *L*-mimosine analogues and ii) assess their anticancer activity (e.g. viability, apoptosis, necrosis, ROS and cell cycle growth arrest) in an in vitro model of human malignant melanoma consisting of melanoma (A375), non-melanoma (A431) and non-malignant immortalized keratinocyte (HaCaT) cells. The latter cell line was utilized as a control, non-malignant one (predominantly existing in the epidermis thereby surrounding a malignant melanocyte) allowing us to determine potential “side” cytotoxicity. Finally, the non-melanoma cells provide a means of assessing the specificity of an observed anticancer activity between melanoma and non-melanoma skin cancer cells.

## Materials and methods

### Chemicals, equipment and organic synthesis

All chemical reagents were purchased from Sigma-Aldrich (St. Louis, MO, USA), Alfa Aesar (Lancashire, UK), Fluorochem (Derbyshire, UK), TCI (Oxford, UK) and were used without any further purification. All chemical solvents were purchased from Fisher Scientific (Hampton, NH, USA) and Sigma Aldrich (St. Louis, MO, USA), at either HPLC or reagent grade. When required, solvents were dried over activated 4 Å molecular sieves.

NMR Spectroscopy was performed on JEOL ELS400 Delta Spectrometer at frequencies of 400 MHz for ^1^H NMR, 101 MHz for ^13^C NMR. Chemical shifts were recorded as parts per million (ppm) with tetramethylsilane (TMS) as the internal standard. Solvents used included deuterated dimethyl sulfoxide (DMSO-*d*_*6*_), deuterated chloroform (CDCl_3_), deuterated methanol (MeOH-*d*_*4*_), deuterated water (D_2_O) and deuterated TFA (CF_3_CO_2_D). Chemical shifts were observed with integrals, splitting and J values, multiplicity of the signals were recorded as singlet (s), doublet (d), triplet (t), quartet (q). In addition, the multiplicities (which have further coupling) were recorded e.g. double doublet (dd). High Resolution Mass Spectrometry (HRMS) was performed on Thermo Q-Exactive spectrometer with electrospray ionisation (ESI) (Thermo Fisher Scientific, Cramlington, UK) while High Performance Liquid Chromatography (HPLC) (Agilent Technologies, 1260 Infinity) analysis was carried out on a Phenomenex Column (HYPERSIL 5u C18, 150 × 4.60 mm). Flash Chromatography was performed on Biotage® Isolera One using Biotage® SNAP-Ultra flash chromatography cartridges 10-100 g size (Biotage, Uppsala, Sweden).

Finally, detailed description of the synthesis and characterization of the screened molecules is shown in the Supplementary Material**.**

### Cell lines

The human malignant melanoma (A375) and epidermoid carcinoma (A431) cell lines were purchased from Sigma-Aldrich (St. Louis, MO, USA). The human immortalized keratinocyte (HaCaT) cell line was kindly provided by Dr. Broby (Public Health England, UK). All cell lines were authenticated with the STR method and were also tested for mycoplasma contamination. In addition, they were maintained in DMEM medium with high glucose content, supplemented with 10% FBS, 2 mM L-glutamine, and 1% pen/strep (100 U/mL penicillin, 100 μg/mL streptomycin) and cultured in a humidified atmosphere at 37 °C and 5% CO_2_, grown as monolayers and sub-cultured at 80–90% confluency. All cell lines were cultured for 15–20 passages before new stocks were utilized. Finally, all media and reagents were purchased from Labtech (East Sussex, UK) whereas all cell culture plasticware were obtained from Corning (Corning, NY, USA).

### Determination of cell viability

The Alamar-blue assay was utilized in this set of experiments. Briefly, A375, A431, and HaCaT cells were seeded in 100 μL/well into 96-well plates and incubated overnight prior to exposure to each of the hydroxypyridone compounds (e.g. compounds 10, 11, 18, 22, 23 and 29). Density of A375 cells was 8000, 4000, 2000 cells/well and for A431 and HaCaT cells 10,000, 5000, 2500 cells/well for 24, 48 and 72 h, respectively. On the following day, cells were exposed to a range of concentrations (10–1000 μM) over different incubation periods. For control conditions, cells were incubated with complete medium only. At the indicated time points, resazurin [dissolved in PBS (1 mg/ml final concentration)] was added in an amount equal to 1/10 of the volume in each well and incubated for 2–4 h (depending on the type of cancer cell line), at 37 °C. The plates were then centrifuged, and absorbance was recorded at 570 nm and 600 nm (reference wavelength) using a Spark multimode plate reader (Tecan, Switzerland). The levels of cell viability were estimated and expressed as percentage of control cells.

### Morphological observation of cells

A375 cells were seeded in 100 mm dishes and exposed to either complete medium only (control) or 100 μM of compound 22 for 24, 48 and 72 h. The density of A375 cells was 1.4 × 10^6^, 0.7 × 10^6^ and 0.4 × 10^6^ per dish for 24, 48 and 72 h, respectively. At the indicated time points, the morphology of cells was observed by an inverted phase contrast microscope (ZOE fluorescent cell imager, Bio-rad, CA, USA) and images were captured at 20x magnification.

### Determination of ROS

A375 cells were seeded in 100 mm dishes (1.4 × 10^6^, 0.7 × 10^6^ and 0.4 × 10^6^ per dish for 24, 48 and 72 h, respectively) and exposed to 100 μM of compound 22. Then, they were harvested and washed twice with PBS and a single cell suspension of 10^6^ cells/mL was prepared. Dihydrorhodamine 123 (DHR 123; 10 μM) was added in the suspension and incubated for 5 min at 37 °C. Then, DAPI (1 μM) was added to each sample and incubated for 5 min in order to determine the percent of dead cells in the suspension. Data acquisition and analysis of 10,000 events, for each sample, was performed using a FACS Canto II flow cytometer (BD Biosciences, San Jose, CA, USA). DAPI-positive cells were excluded from further analysis of the results.

### Determination of apoptosis

The CellEvent Caspase 3/7 Green flow cytometry assay kit was utilized for the detection of apoptosis according to the manufacturer’s instructions. Briefly, cells were plated into 100 mm dishes and allowed to adhere overnight. Density of A375 cells was 1.4 × 10^6^, 0.7 × 10^6^ and 0.4 × 10^6^ cells per dish for 24, 48 and 72 h, respectively. The next day, cells were exposed to 100 μM of compound 22 for 24, 48 and 72 h. Next, cells were harvested, washed twice with PBS and a single cell suspension of 10^6^ cells/mL was prepared. Then, 0.5 μL of CellEvent Caspase 3/7 Green detection reagent was added into 0.5 mL of each cell suspension and samples were incubated at 37 °C for 30 min. Five (5) min prior to the end of the incubation period, 1 μM of DAPI was added. Data acquisition and analysis of 20,000 events, for each sample, was performed using a FACS Canto II flow cytometer (BD Biosciences, San Jose, CA, USA). Caspase-3/7-positive cells were identified as apoptotic whereas DAPI-positive cells as necrotic.

### Determination of cell cycle kinetics

The FxCycle PI/RNase staining solution was used according to the manufacturer’s instructions. Following exposure to 100 μM of compound 22, cells were harvested and washed twice with PBS. The density of A375 cells was 1.4 × 10^6^, 0.7 × 10^6^ and 0.4 × 10^6^ cells per dish for 24, 48 and 72 h, respectively. Approximately 0.5 × 10^6^ cells were fixed in cold 70% ethanol, for 1 h or longer, at 4 °C until further processing. Cells were then washed twice with PBS to remove ethanol and finally suspended in FxCycle PI/RNase staining solution for 30 min at RT in the dark. Data acquisition and analysis of 10,000 events, for each sample, was performed using a FACS Canto II flow cytometer (BD Biosciences, San Jose, CA, USA).

### Preparation of cell lysates and protein determination

A375 cells were plated in 100 mm dishes and cultured overnight at 37 °C at a density of 1.4 × 10^6^, 0.7 × 10^6^ and 0.4 × 10^6^ cells per dish for 24, 48 and 72 h respectively Next day, cells were treated with 100 μM of compound 22 for 24, 48 and 72 h and then trypsinized, washed twice with ice-cold PBS and pellets were collected after centrifugation at 2000 rpm for 3 min at 4 °C. Cell pellets were then lysed in lysis buffer (10 mM HEPES at pH 7.9, 10 mM KCl, 0.1 mM EDTA, 1.5 mM MgCl_2_, 0.2% NP40) and supplemented with Protease Inhibitor Tablets (Thermo Scientific, Waltham, MA, USA). Then, they were left on ice while periodically vortexed over a 30 min period and sonicated (three cycles at 10 amplitudes for 20 s on ice) to disrupt cellular membranes. Cell lysates were centrifuged at full speed (15,000 *rpm*) for 10 min at 4 °C and supernatants were transferred in new tubes. Protein content was determined by utilizing the BCA protein assay kit (Thermo Scientific, Waltham, MA, USA), according to the manufacturer’s protocols. Protein extracts were stored at −20 °C until usage.

### Western immunoblotting

Forty micrograms (40 μg) of cytoplasmic protein extracts were separated by SDS-polyacrylamide gels and transferred electrophoretically onto PVDF membranes (either 0.45 or 0.2 μm) using mini gel tank sand mini blot modules (Invitrogen, Carlsbad, CA, USA). The blots were then blocked in 5% non-fat milk powder in TBST buffer (50 mM Tris-HCl, 150 mM NaCl at pH 7.6 and 0.1% Tween-20) for 2 h at RT. After blocking, membranes were washed three times with TBST and incubated overnight at 4 °C, under agitation, with the appropriate primary antibody (e.g. 1:1000 for anti-Caspase-8, anti-Caspase-9, anti-Apaf-1, anti-BID, and 1:20000 for anti-Tubulin) and according to the manufacturer’s protocol. All antibodies were purchased from Cell Signaling Technology (Danvers, MA, USA). Next day, membranes were incubated with the appropriate horseradish peroxidase-conjugated secondary antibody (mouse or rabbit at 1:1000) for 1 h at RT, under agitation, after being washed three times with TBST. Membranes were washed three times with TBST and labelled protein bands were detected by utilizing the SuperSignal West Pico PLUS Chemiluminescent Substrate kit from Thermo Scientific (Waltham, MA, USA) according to the manufacturer’s protocol. Protein bands were visualized with the use of the G:BOX Chemi XX6/XX9 gel imaging system (Syngene, Cambridge, UK).

### Statistical analysis

Data were expressed as mean values ± standard deviation (SD) and comparisons were made between control and treated groups. Statistical analyses were performed by one-way ANOVA with Tukey’s test for multiple comparisons after using the SPSS v.22 software. Finally, statistical significance was set at *p* < 0.05, *p* < 0.01 and *p* < 0.001.

## Results

**Fig. 2:**
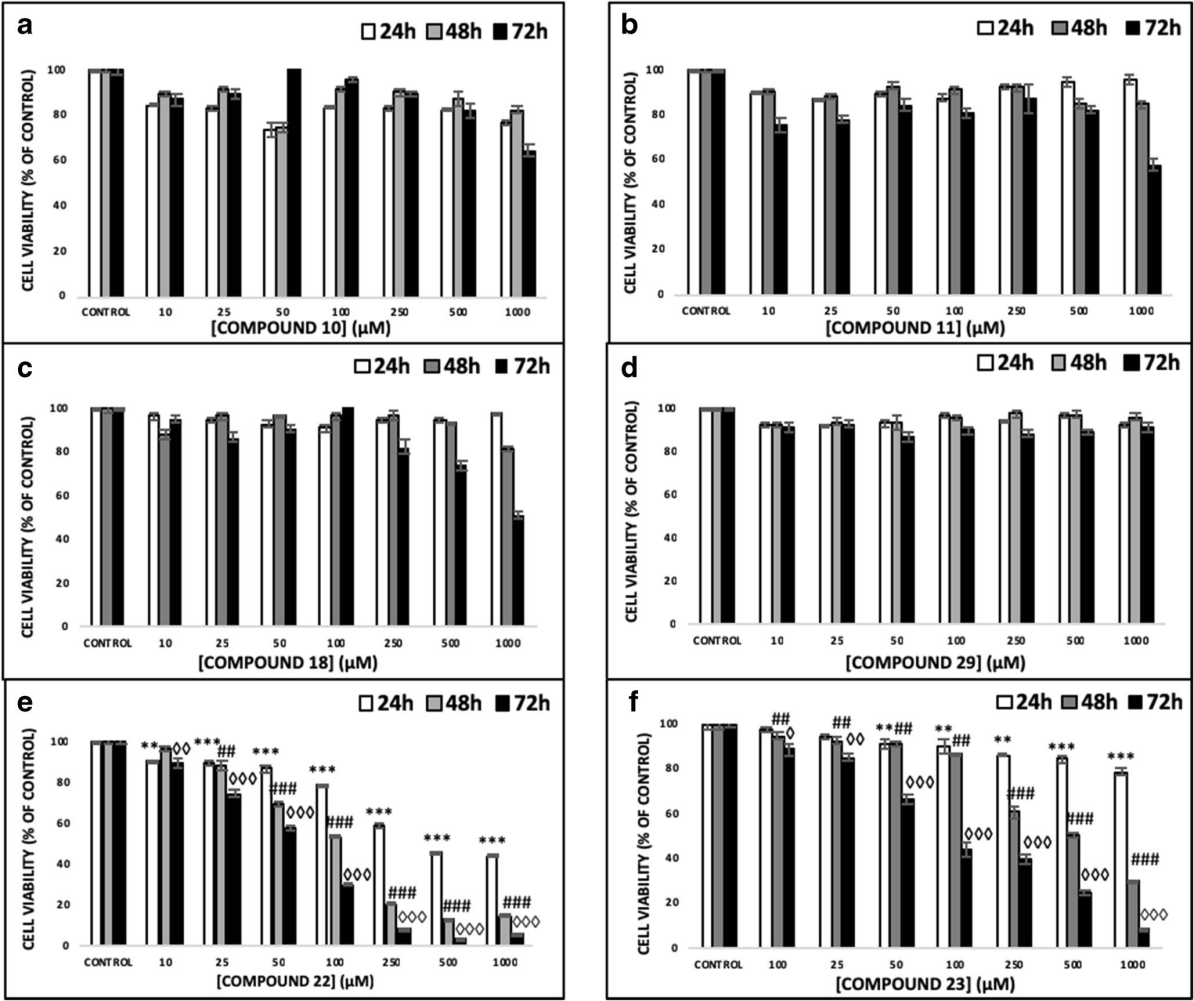
The ability of hydroxypyridones to induce cytotoxicity in A375 cells. Cells were exposed to a range of 10–1000 μM concentrations of (**a**) compound 10, (**b**) compound 11, (**c**) compound 13, (**d**) compound 29, (**e**) compound 22 and (**f**) compound 23 for 24, 48 and 72 h. Data shown are means ± SD of 5 replicates from three independent experiments. Asterisk (*), hashtag (#) or rhombus (◊) denote statistical significance when compared to their respective controls at *p* < 0.05. **, ## and ◊◊ denote statistical significance at *p* < 0.01 whereas ***, ###, ◊◊◊ at *p* < 0.001

**Fig. 3:**
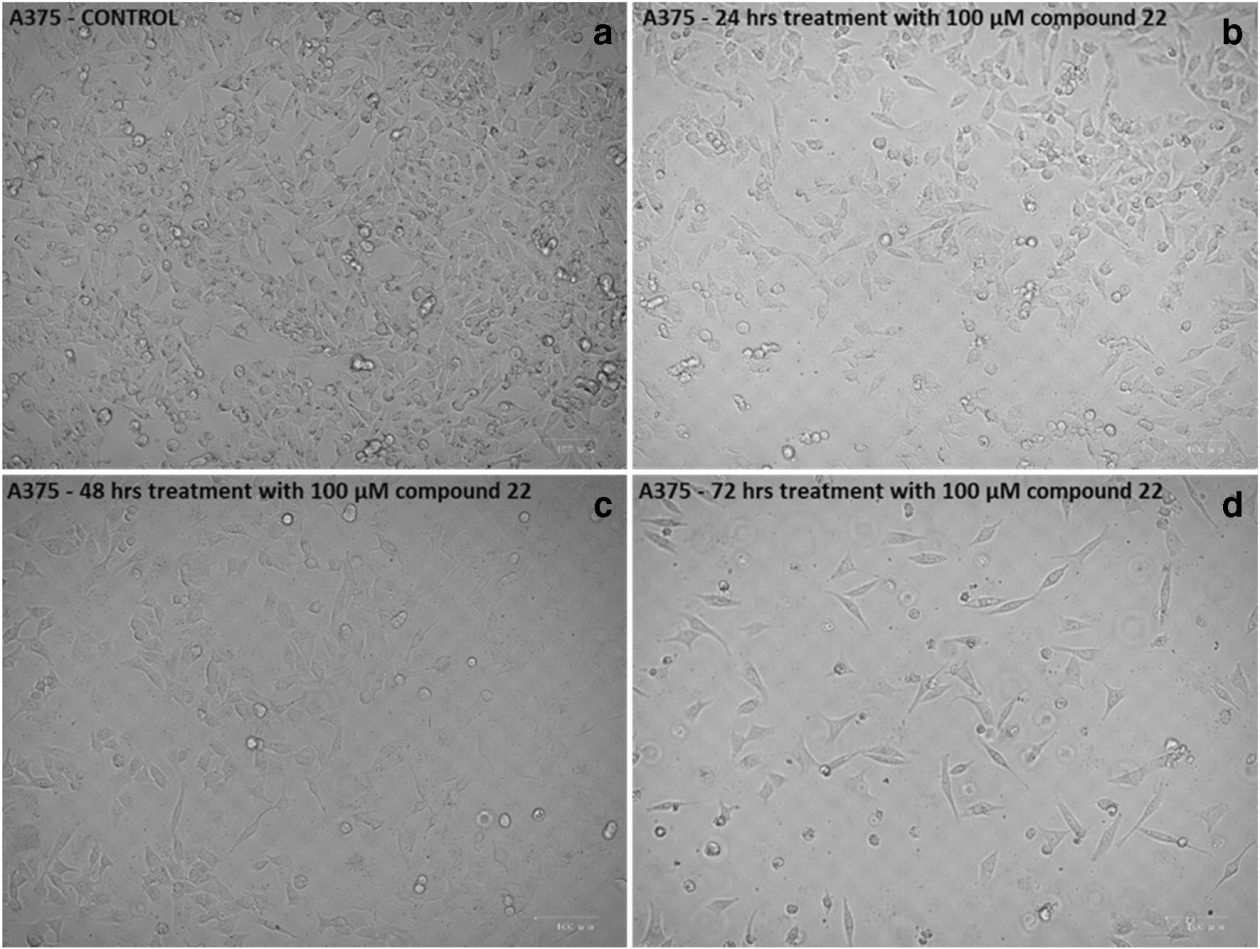
The ability of compound 22 to induce cytotoxicity in A375 cells. Control cells (**a**) and those exposed to 100 μM of compound 22 at 24 h (**b**), 48 h (**c**) and 72 h (**d**) were visualized under inverted phase contrast microscope

**Fig. 4:**
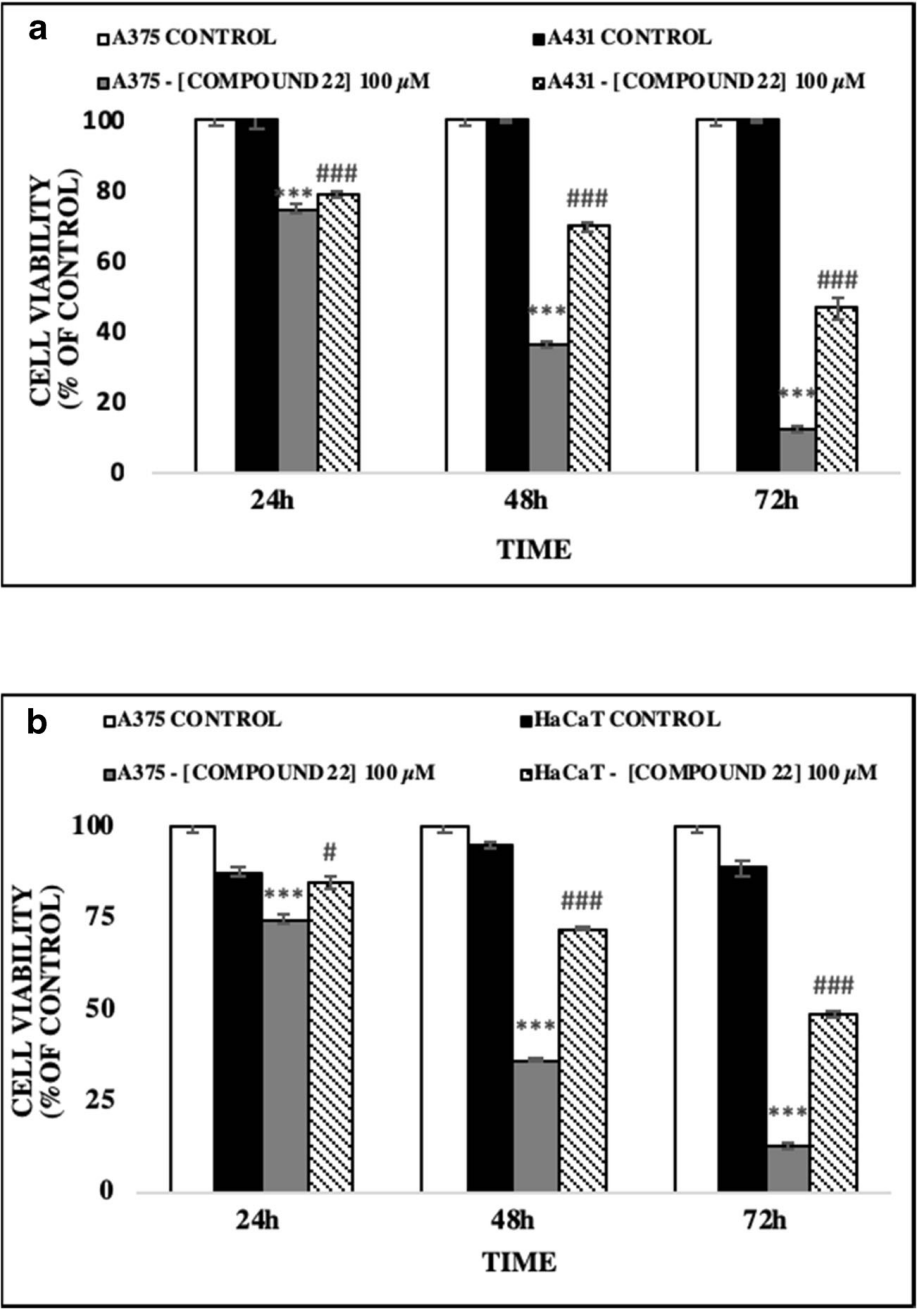
The ability of compound 22 to induce cytotoxicity in (**a**) non-melanoma (A431) cells and (**b**) non-malignant keratinocyte (HaCaT) cells. Cells were exposed to100 μM of compound 22 for 24, 48 and 72 h. Data shown are means ± SD of 5 replicates from three independent experiments. Asterisk (*), hashtag (#) or rhombus (◊) denote statistical significance when compared to their respective controls at *p* < 0.05. **, ## and ◊◊ denote statistical significance at p < 0.01 whereas ***, ###, ◊◊◊ at *p* < 0.001

The anticancer activity of a number of hydroxypyridinone (HOPO) core metal chelators was evaluated in human malignant melanoma (A375) cells including those of 1,2-HOPO (compound 18), 2,3-HOPO (compound 29), 3,4-HOPO (compounds 11, 22, 23) and hydroxypyranone (compound 10). Each compound was assessed at a range of concentrations (10–1000 μM) and time points (24–72 h). Our results revealed that A375 cells were resistant to exposure with compounds 10 and 29 while compounds 11 and 18 only showed noteworthy activity at concentrations as high as 1000 μM, after 72 h treatment (Figs. 2a–d). However, compounds 22 and 23 showed significant time- and concentration-dependent cytotoxicity. More specifically, cell viability levels reached an EC_50_ at a concentration of 100 μM after 48 and 72 h of exposure to compounds 22 and 23 respectively (Figs. 2e and f). Furthermore, at 24 h of exposure with compounds 22 and 23 cell viability levels were either decreased or remained almost at control levels respectively. Overall, treatment with compound 22 was more potent (EC_50_ value of 100 μM) when compared to compound 23 (EC_50_ value of approximately 250 μM), after 48–72 h of exposure, an observation which was also evident under inverted phase contrast microscopy (Fig. 3). Although it is well established that both enantiomers have similar activity, the preference for the *L*-enantiomer (compound 22) is likely explained by the slight preference of LAT-1 for the *L*-enantiomers of amino acids [44]. Interestingly, the observed order of activity (compounds 22 and 23 > > compound 18 > compound 11 > compound 10 = compound 29) does not match the order expected only from their metal binding efficiency (3,4-HOPO > > 3,2-HOPO ≈ 1,2- HOPO > hydroxypyranones) [44–47]. This suggests that the activity is guided by a more complex combination of molecular properties. Finally, we evaluated the observed cytotoxic potency of compound 22 (at 100 μM) in non-melanoma (A431) as well as non-malignant keratinocyte (HaCaT) cells in an attempt to document any potential specificity towards A375 cells. Our observations revealed that A431 and HaCaT cells were also affected by compound 22 but nevertheless were shown to be more resistant than A375 cells (Figs. 4a and b). Taken together, our data indicate that compound 22 exerts a higher degree of potency against A375 cells while A431 and HaCaT cells remain considerably more resistant.

**Fig. 5:**
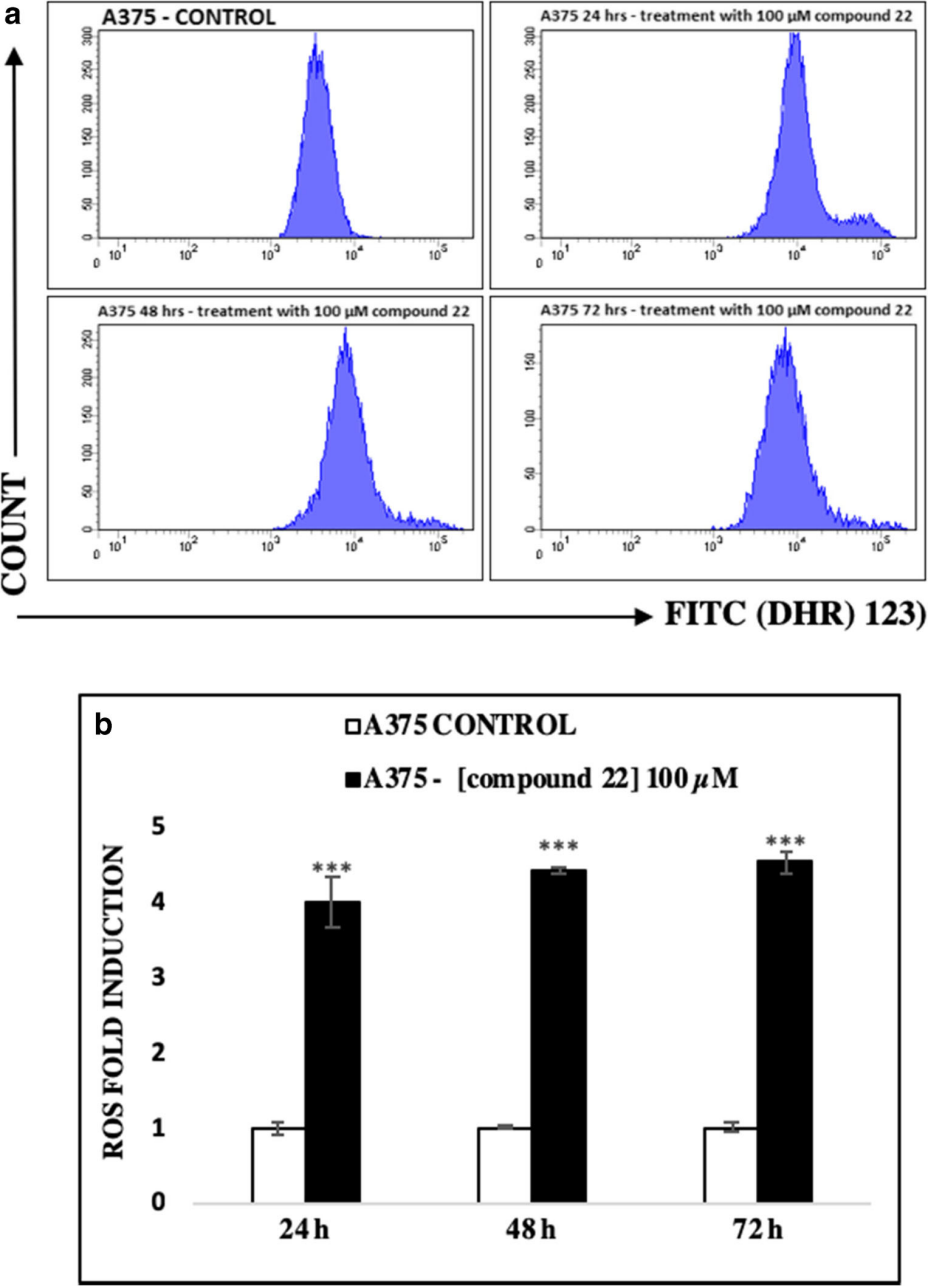
The ability of compound 22 to induce generation of oxidative stress in A375 cells. Cells were exposed to 100 μM of compound 22 for 24, 48 and 72 h and monitored by means of (**a**) flow cytometry in addition to being quantitated as (**b**) ROS fold induction. Data shown are means ± SD of 3 replicates from three independent experiments. Asterisks (***) denote statistical significance at *p* < 0.001 when compared to the respective controls after of exposure

On the other hand, the ability of compound 22 to induce intracellular ROS generation was also evaluated by means of flow cytometry and after utilizing a fluorescent DHR 123 probe as a ROS detector. Our data showed that the FITC spectrum increased dramatically in the exposed group compared to the unexposed (control) one (Fig. 5a). Interestingly, treatment of A375 cells with 100 μM of compound 22 induced a significant increase in intracellular ROS levels, during the first 24 h, which were sustained at each time point thereafter (Fig. 5b).

**Fig. 6:**
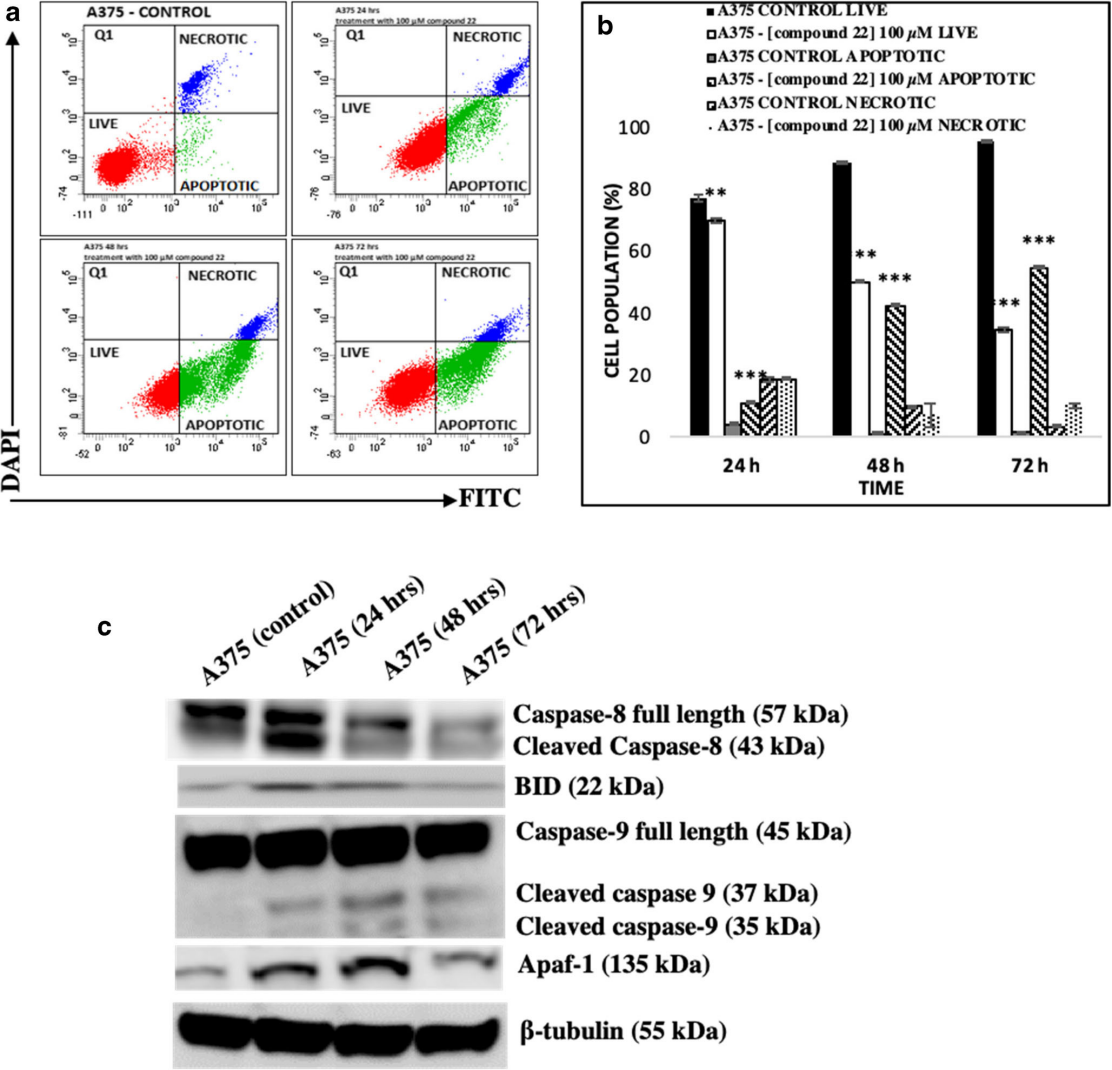
The ability of compound 22 to induce apoptosis in A375 cells. Briefly, cells were exposed to 100 μM of compound 22 at 24, 48 and 72 h and then the number of live, apoptotic and necrotic cells were recorded by means of (**a**) flow cytometry and also quantified as (**b**) percent of total cell population. Data shown are means ± SD of 3 replicates from three independent experiments. Asterisks (**) and (***) denote statistical significance at p < 0.01 and p < 0.001 respectively when compared to their respective control (untreated cells); (**c**) The ability of compound 22 to induce the expression of intrinsic and extrinsic apoptotic markers in A375 cells. Cells were subjected to 100 μM of compound 22 for 24, 48 and 72 h and protein expression levels of full length and cleaved caspases-8 and -9 were recorded in addition to those of BID and Apaf-1

Moreover, the number of apoptotic and necrotic A375 cells was evaluated after exposure to compound 22. To distinguish between these two modes of cell death, the CellEvent Caspase 3/7 Green detection reagent was utilized as an activated caspase 3/7 activity indicator whereas DAPI as an indicator for necrosis (Fig. 6a). Our data showed significant cell death during the first 24 h, an effect which was intensified (over time) in a manner where live cells were reduced while necrotic cells remained at steady levels (Fig. 6b). In addition, a more detailed characterization of various key proteins representative of the intrinsic and extrinsic apoptotic pathways was performed (by western immunoblotting) in A375 cells exposed to compound 22 (at 100 μM). The expression levels of cleaved and full length Caspase-8 were indicative of the involvement of the extrinsic apoptotic pathway [48–51] whereas those of Caspase- 9 and Apoptotic protease-activating factor 1 (Apaf-1) for the intrinsic one [52–55]. In addition, expression levels of BID were reduced representative of the well-established interplay between the two apoptotic pathways [50]. Overall, the activation of Caspase-8 was evident by the presence of increased cleaved and/or reduced un-cleaved “full” length protein expression levels observed as early as 24 h after exposure (and remained as such throughout the entire time-course) thus indicating the activation of the extrinsic apoptotic pathway (Fig. 6c). Furthermore, the expression levels of BID were also shown to be elevated at 24–48 h (indicative of a concomitant activation of the intrinsic apoptotic pathway) while were reduced back to control levels after 72 h of exposure (Fig. 6c). Further evidence for the concomitant activation of the intrinsic apoptotic pathway was revealed after examining the expression levels of Caspase-9 and Apaf-1 throughout the time-course (Fig. 6c). To this end, several studies have shown that activation of Caspase-8 as well as that of Caspase-9 and Apaf-1 (via formation of the apoptosome) can lead to the activation of Caspase-3 which is an established mechanism for the execution of apoptosis [50–59].

**Fig. 7:**
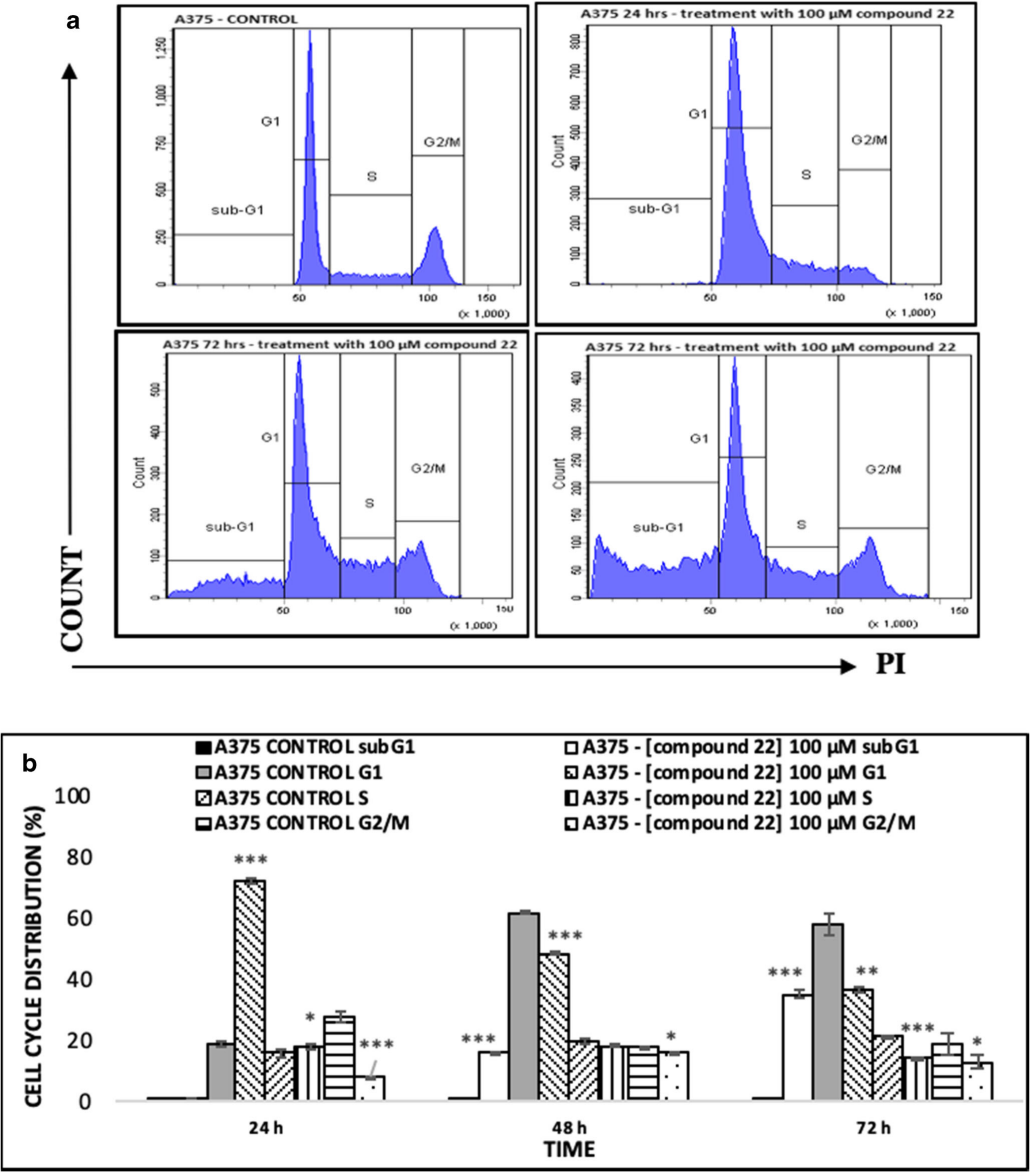
The ability of compound 22 to induce cell cycle growth arrest in A375 cells. Cells were exposed to 100 μM of compound 22 at 24, 48 and 72 h and then the number of cells were recorded at each stage of the cell cycle by means of (**a**) flow cytometry and also quantified as (**b**) percent of total DNA cellular content accumulated at each phase of the cell cycle (e.g. sub-G1, G1, S or G2/M). Data shown are means ± SD of 3 replicates from three independent experiments. Asterisks (***) denote statistical significance at *p* < 0.01 and *p* < 0.001 respectively when compared to their respective control (untreated cells)

Finally, the ability of compound 22 to induce cell cycle growth arrest was assessed by using the FxCycle PI/RNase staining solution for quantification of DNA content under each phase of the cell cycle and subsequent analysis by flow cytometry (Fig. 7a). Our results show that 24 h of exposure cause a statistically significant elevation of the G1 phase followed by a reduction of the G2/M phase while the S phase remained unaffected. Interestingly, at 48 h, a significant increase of the sub-G1 phase was also observed followed by a marked reduction of the G1 phase while the S- and G2/M phases remained relatively unaffected. Furthermore, this effect was intensified at 72 h of exposure (Fig. 7b).

## Discussion

The capacity of metal chelators to act as anticancer agents (and potentially as clinically effective treatment options) has been reported in various in vitro cancer models including malignant melanoma and leukemia [60, 61]. During our study, a series of hydroxypyridinones-based analogues of *L-*mimosine were synthesized and their anticancer activity was evaluated against an in vitro model of human malignant melanoma. Of these, a methylated analogue (*L*-*N*- substituted 3.4-HOPO) of *L*-mimosine (compound 22) was shown to be the most cytotoxic with a higher degree of potency in A375 cells compared to A431 and HaCaT cells. Previous experimental studies, utilizing dipeptides of *L-*mimosine against melanoma, suggested that the selectivity of this class of HOPO-based molecules was mainly due to their ability to inhibit and consequently down-regulate tyrosinase which causes perturbations in melanin production [62, 63] the levels of which are increasing in metastatic melanoma thereby reducing the outcome of radiotherapy. On such basis, it has been postulated that inhibition of melanogenesis could potentially improve the therapeutic outcome of radiotherapy [64, 65].

On another note, hydroquinone core-based precursors of melanin can act as redox cyclers mainly in the presence of iron species [66–68]. Previously, it has been shown that combinational therapy with metal chelators and Celecoxib (a drug acting as a COX-2 inhibitor) can dramatically suppress metastatic melanoma by inhibiting COX-2 associated cell signalling pathway(s) [69]. Additionally, other experimental studies have shown that melanoma cells produce large amounts of extracellular superoxide anion (compared to normal melanocytes) suggesting that they are constantly exposed to an oxidative stress environment induced by elevated levels of intracellular ROS [70]. To this end, we have demonstrated that treatment with 100 μM of compound 22 stimulates the production of intracellular ROS (by approximately 4-fold) compared to the untreated control, at 24 h post-treatment, and this effect was retained over the entire time-course. Despite the fact that HOPOs are known to acts as antioxidants, compound 22 has been shown to induce generation of ROS presumably as a consequence of pro-oxidant effects [71, 72]. Nevertheless, this finding is in agreement with another study utilizing glioma cells treated with *L-*mimosine during which the authors observed apoptotic induction as the result of the release of mitochondria-derived ROS together with activation of p38 and JNK [73]. Finally, another study has shown that a different class of metal chelators (i.e. thiosemicarbazones) have the ability to induce cytotoxicity on melanoma cells by disrupting the cellular antioxidant defence system thus causing elevation of intracellular ROS levels [74].

In investigating further into the anti-proliferative effect(s) of compound 22, we focused our efforts into the mode of cell death activation. Our findings revealed triggering of apoptosis evident by the induction of both extrinsic and intrinsic cascades (via activation of caspases-8 and -9 respectively) suggesting that compound 22 induces apoptosis in a similar manner to *L-*mimosine [73, 75, 76]. To this end, another study has shown that melanoma cells induce apoptosis in response to treatment with metal chelators in an attempt to compensate for an increased load of ROS [77]. On the other hand, ROS can act as second messengers capable of regulating several diverse cellular functions including cell survival and proliferation [78]. Therefore, elevated levels of ROS can trigger the activation of caspases thus initiating apoptosis. In fact, lower ROS concentrations have been shown to induce cell survival responses whereas higher ones can activate death processes including apoptosis [79]. Last but not least, metal chelators can inhibit cell growth by either inducing apoptosis such as in Kaposi sarcoma cells [80] and/or by blocking cell cycle progression like in the cases of breast [32] and lung [81] cancer cells. To our knowledge, this is the first report that (i) presents the screening of newly synthesized hydroxypyridinone-based analogues of *L*-mimosine and also (ii) describes how compound 22 exerts its anticancer activity against an in vitro model of human malignant melanoma. This is of utmost importance as there is a great need to synthesize novel metal chelators with the capacity to minimize side-effects and increase therapeutic effectiveness. In the clinical setting, this is of particular interest as it could potentially translate into better therapeutic management and consequently better quality of life in these patients.

## Electronic supplementary material

ESM 1 Additional figures illustrating purity data; (HPLC chromatographs), structure verification; ^1^H-NMR, ^13^C-NMR and HRMS spectra for all the intermediates and final products generated in this study. This material is available to authorized users. (PDF 4717 kb)
