# Supplementary material for: Anticancer activity of a novel methylated analogue of L-mimosine against an in vitro model of human malignant melanoma
Source: Invest New Drugs. 2019 Jun 26;38(3):621–33. doi: 10.1007/s10637-019-00809-0 (PMC7211211; doi:10.1007/s10637-019-00809-0)
Supplement: Supplementary file 1 — Additional figures illustrating purity data; (HPLC chromatographs), structure verification; 1H-NMR, 13C-NMR and HRMS spectra for all the intermediates and final products generated in this study. This material is available to authorized users. (PDF 4717 kb) [file 10637_2019_809_MOESM1_ESM.pdf]

SUPPLEMENTARY MATERIAL

Anticancer activity of a novel methylated analogue of *L*-mimosine against an *in vitro* model of human malignant melanoma

Sotiris Kyriakou[1], Melina Mitsiogianni[1], Theodora Mantso[1], William Cheung[1], Stephen Todryk[1], Stephany Veuger[1], Aglaia Pappa[2], David Tetard[*1] and Mihalis I. Panayiotidis[*1]

[1]Department of Applied Sciences, Northumbria University, Ellison Building, NE1 8ST, Newcastle Upon Tyne, UK; [2]Department of Molecular Biology & Genetics, Democritus University of Thrace, Alexandroupolis, 68100, Greece

TABLE OF CONTENTS

ORGANIC SYNTHESIS 5

Synthesis of *rac*-10 and *rac*-11 5

Synthesis of *rac*-18........ 9

Synthesis of *L*-22 and *D*-23 14

Synthesis of *rac*-29........ 17

PROOF OF PRODUCTS PURITY BY HPLC........ 21

Figure S1: Analytical HPLC chromatogram of compound 10 21

Figure S2: Analytical HPLC chromatogram of compound 11 21

Figure S3: Analytical HPLC chromatogram of compound 18 22

Figure S4: Analytical HPLC chromatogram of compound 22 22

Figure S5: Analytical HPLC chromatogram of compound 29 23

^{1}H AND ^{13}C-NMR FOR ALL INTERMEDIATES AND FINAL PRODUCTS; HRMS FOR NOVEL COMPOUNDS AND FINAL PRODUCTS........ 24

Figure S6: (A) ^{1}H-NMR spectra of 5-(benzoyloxy)2-(hydroxymethyl)-4H-pyran-4-one (7) at 400 MHz in DMSO-d_6 and (B) ^{13}C-NMR spectra of 5-(benzoyloxy)2-(hydroxymethyl)-4H-pyran-4-one (7) at 100 MHz in DMSO-d_6. 25

Figure S7: (A) ^{1}H-NMR spectra of [5-(benzyloxy)-4-oxo-4H-pyran-2-yl]methyl 4-methylbenzene-1-sulfonate (8) at 400 MHz in $CDCl_3$, (B)^{13}C-NMR spectra of [5-(benzyloxy)-4-oxo-4H-pyran-2-yl]methyl 4-methylbenzene-1-sulfonate (8) at 100 MHz in $CDCl_3$ and (C) HRMS of [5-(benzyloxy)-4-oxo-4H-pyran-2-yl]methyl 4-methylbenzene-1-sulfonate (8)........ 27

Figure S8: (A) ^{1}H-NMR spectra of 1,3-diethyl 2-{[5-(benzyloxy)-4-oxo-4H-pyran-2-yl]methyl}-2-acetamidopropanedioate (9) at 400 MHz in DMSO-d_6 and (B)^{13}C-NMR spectra of 1,3-diethyl 2-{[5-(benzyloxy)-4-oxo-4H-pyran-2-yl]methyl}-2-acetamidopropanedioate (9) at 100 MHz in DMSO-d_6 and (C) HRMS of 1,3-diethyl 2-{[5-(benzyloxy)-4-oxo-4H-pyran-2-yl]methyl}-2-acetamidopropanedioate (9) 29

Figure S9: ^{1}H-NMR spectra r*ac*-2-amino-3-(5-hydroxy-4-oxo-4H-pyran-2-yl)propanoic acid (10) at 400 MHz in D_2O/CF_3CO_2D and (B)^{13}C-NMR spectra of *rac*-2-amino-3-(5-hydroxy-4-oxo-4H-pyran-2-yl)propanoic acid (10) at 100 MHz in D_2O/CF_3CO_2D and (C) HRMS of *rac*-2-amino-3-(5-hydroxy-4-oxo-4H-pyran-2-yl)propanoic acid (10). 31

Figure S10: ^{1}H-NMR spectra *rac*-2-amino-3-(5-hydroxy-4-oxo-1,4-dihydropyridin-2-yl)propanoic acid (11) at 400 MHz in D_2O/CF_3CO_2D and (B)^{13}C-NMR spectra of *rac*-2-amino-3-(5-hydroxy-4-oxo-1,4-dihydropyridin-2-yl)propanoic acid (11) at 100 MHz in D_2O/CF_3CO_2D and (C) HRMS of *rac*-2-amino-3-(5-hydroxy-4-oxo-1,4-dihydropyridin-2-yl)propanoic acid (11) 33

Figure S11: (A) ^{1}H-NMR spectra of 5-bromo-2-methoxypyridine (12) at 400 MHz in $CDCl_3$, (B)^{13}C-NMR spectra of 5-bromo-2-methoxypyridine (12) at 100 MHz in $CDCl_3$. 34

Figure S12: (A) ^{1}H-NMR spectra of 6-methoxypyridine-3-carbaldehyde (13) at 400 MHz in $CDCl_3$, (B)^{13}C-NMR spectra of 6-methoxypyridine-3-carbaldehyde (13) at 100 MHz in $CDCl_3$ 35

Figure S13: ^{1}H-NMR spectra (4Z)-4-[(6-methoxypyridin-3-yl)methylidene]-2-methyl-4,5-dihydro-1,3-oxazol-5-one (14) at 400 MHz in $CDCl_3$ and (B)^{13}C-NMR spectra of (4Z)-4-[(6-methoxypyridin-3-yl)methylidene]-2-methyl-4,5-dihydro-1,3-oxazol-5-one (14) at 100 MHz in $CDCl_3$ and (C) HRMS of (4Z)-4-[(6-methoxypyridin-3-yl)methylidene]-2-methyl-4,5-dihydro-1,3-oxazol-5-one (14). 37

Figure S14: ^{1}H-NMR spectra of (2Z)-2-acetamido-3-(6-methoxypyridin-3-yl)prop-2-enoic acid (15) at 400 MHz in $CDCl_3$ and (B)^{13}C-NMR spectra of (2Z)-2-acetamido-3-(6-methoxypyridin-3-yl)prop-2-enoic acid (15) at 100 MHz in $CDCl_3$ and (C) HRMS of (2Z)-2-acetamido-3-(6-methoxypyridin-3-yl)prop-2-enoic acid (15) 39

Figure S15: ^{1}H-NMR spectra of *rac*-2-acetamido-3-(6-methoxypyridin-3-yl)propanoic acid (16) at 400 MHz in MeOH-d_4 and (B)^{13}C-NMR spectra of *rac*-2-acetamido-3-(6-methoxypyridin-3-yl)propanoic acid (16) at 100 MHz in MeOH-d_4 and (C) HRMS of *rac*-2-acetamido-3-(6-methoxypyridin-3-yl)propanoic acid (16) 41

Figure S16: ^{1}H-NMR spectra of rac-5-(2-carboxy-2-acetamidoethyl)-2-methoxypyridin-1-ium-1-olate (17) 43

at 400 MHz in DMSO-d_6 and (B)^{13}C-NMR spectra of rac-5-(2-carboxy-2-acetamidoethyl)-2-methoxypyridin-1-ium-1-olate (17) at 100 MHz in DMSO-d_6 and (C) HRMS of rac-5-(2-carboxy-2-acetamidoethyl)-2-methoxypyridin-1-ium-1-olate (17) 43

Figure S17: ^{1}H-NMR spectra of *rac*-2-amino-3-(1-hydroxy-6-oxo-1,6-dihydropyridin-3-yl)propanoic acid (18) 45

Figure S18: (A) ^{1}H-NMR spectra of 3-(benzyloxy)-2-methyl-1,4-dihydropyridin-4-one (19) at 400 MHz in $CDCl_3$, (B)^{13}C-NMR spectra of 3-(benzyloxy)-2-methyl-1,4-dihydropyridin-4-one (19) at 100 MHz in $CDCl_3$.......... 46

Figure S19: (A) ^{1}H-NMR spectra of (2L)-3-amino-2-{[(tert-butoxy)carbonyl]amino}propanoic acid (20) at 400 MHz in DMSO-d_6 and (B)^{13}C-NMR spectra of (2L)-3-amino-2-{[(tert-butoxy)carbonyl]amino}propanoic acid (20) at 100 MHz in DMSO-d_6.......... 47

Figure S20: (A) ^{1}H-NMR spectra (2L)-2-amino-3-(3-hydroxy-2-methyl-4-oxo-1,4-dihydropyridin-1-yl)propanoic acid (22) at 400 MHz in D_2O/CF_3CO_2D and (B)^{13}C-NMR spectra of (2L)-2-amino-3-(3-hydroxy-2-methyl-4-oxo-1,4-dihydropyridin-1-yl)propanoic acid (22) at 100 MHz in D_2O/CF_3CO_2D and (C) HRMS of (2L)-2-amino-3-(3-hydroxy-2-methyl-4-oxo-1,4-dihydropyridin-1-yl)propanoic acid (22). 49

Figure S21: (A) ^{1}H-NMR spectra of (2D)-3-amino-2-{[(tert-butoxy)carbonyl]amino}propanoic acid (21) at 400 MHz in DMSO-d6 and (B) ^{13}C-NMR spectra of (2D)-3-amino-2-{[(tert-butoxy)carbonyl]amino}propanoic acid (21) at 100 MHz in DMSO-d6. 50

Figure S22: (A) ^{1}H-NMR spectra (2D)-2-amino-3-(3-hydroxy-2-methyl-4-oxo-1,4-dihydropyridin-1-yl)propanoic acid (23) at 400 MHz in D_2O/CF_3CO_2D and (B)^{13}C-NMR spectra of (2D)-2-amino-3-(3-hydroxy-2-methyl-4-oxo-1,4-dihydropyridin-1-yl)propanoic acid (23) at 100 MHz in D_2O/CF_3CO_2D and (C) HRMS of (2R)-2-amino-3-(3-hydroxy-2-methyl-4-oxo-1,4-dihydropyridin-1-yl)propanoic acid (23). 52

Figure S23: (A) ^{1}H-NMR spectra of 5-(benzyloxy)-2-(hydroxymethyl)-1,4-dihydropyridin-4-one (24) at 400 MHz in DMSO-d_6 and (B)^{13}C-NMR spectra of 5-(benzyloxy)-2-(hydroxymethyl)-1,4-dihydropyridin-4-one (24) at 100 MHz in DMSO-d_6.......... 53

Figure S24: (A) ^{1}H-NMR spectra of 5-(benzyloxy)-4-chloro-2-(chloromethyl)pyridine (25) at 400 MHz in DMSO-d_6 and (B)^{13}C-NMR spectra of 5-(benzyloxy)-4-chloro-2-(chloromethyl)pyridine (25) at 100 MHz in DMSO-d_6. 54

Figure S25: (A) 1H-NMR spectra of 1,3-diethyl 2-{[5-(benzyloxy)-4-chloropyridin-2-yl]methyl}-2-acetamidopropanedioate (26) at 400 MHz in DMSO-d6 and (B)13C-NMR spectra of 1,3-diethyl 2-{[5-(benzyloxy)-4-chloropyridin-2-yl]methyl}-2-acetamidopropanedioate (26) at 100 MHz in DMSO-d6. 55

Figure S26: (A) ^{1}H-NMR spectra of 1,3-diethyl 2-acetamido-2-[(5-hydroxypyridin-2-yl)methyl]propanedioate (27) at 400 MHz in DMSO-d_6 and (B)^{13}C-NMR spectra of 1,3-diethyl 2-acetamido-2-[(5-hydroxypyridin-2-yl)methyl]propanedioate (27) at 100 MHz in DMSO-d_6. 56

Figure S27: (A) ^{1}H-NMR spectra of 1,3-diethyl 2-acetamido-2-[(5-hydroxy-6-iodopyridin-2-yl)methyl]propanedioate (28) at 400 MHz in DMSO-d_6 and (B)^{13}C-NMR spectra of 1,3-diethyl 2-acetamido-2-[(5-hydroxy-6-iodopyridin-2-yl)methyl]propanedioate (28) at 100 MHz in DMSO-d_6 57

Figure S28: (A) ^{1}H-NMR spectra of rac-2-amino-3-(5-hydroxy-6-oxo-1,6-dihydropyridin-2-yl)propanoic acid (29) at 400 MHz in D_2O/CF_3CO_2D, (B)^{13}C-NMR spectra of rac-2-amino-3-(5-hydroxy-6-oxo-1,6-dihydropyridin-2-yl)propanoic acid (29) at 100 MHz in D_2O/CF_3CO_2D and (C) HRMS of rac-2-amino-3-(5-hydroxy-6-oxo-1,6-dihydropyridin-2-yl)propanoic acid (29). 59

REFERENCES.......... 60

ORGANIC SYNTHESIS

Synthesis of *rac*-10 and *rac*-11

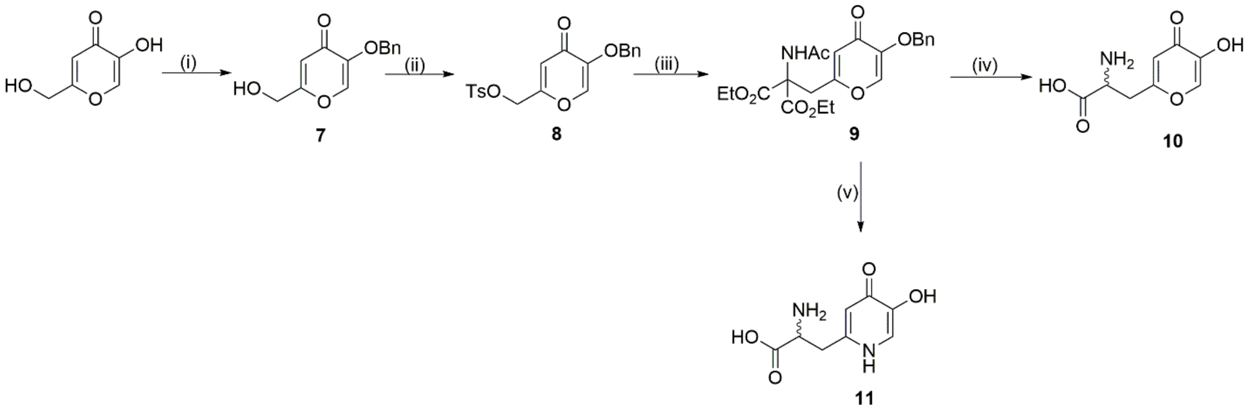

Scheme 1: Reagents and conditions for the synthesis of **compounds 10 and 11**. (**i**) **a**) $NaOH_{(aq)}$, MeOH, 40 mins, 110°C, **b**)BnBr, overnight, 120°C, 87%; (**ii**) TsCl, $NaOH_{(aq)}$, acetone, 20 mins, RT, 94%; (**iii**) Diethyl acetamidomalonate, NaH (60% in mineral oil) $DMF_{(dry)}$, overnight, RT, 95%; (**iv**) **a**) conc. HCl, 180°C, 3 hrs, **b**) conc. NH_4OH, pH 5, 5°C, 89%; (**v**) **a**) conc. NH_4OH, 130°C, 3 hrs, **b**) conc. HCl, 180°C, 3 hrs, **c**) conc. NH_4OH, pH 5, 5°C, 91%.

5-(benzyloxy)-2-(hydroxymethyl)-4*H*-pyran-4-one (compound 7) [1]

A sample of kojic acid (20 g, 141 mmol, 1eq) was dissolved in methanol (80 mL) and mixed with a solution of sodium hydroxide (6.2 g, 155 mmol, 1.1eq) in water (30 mL). The mixture was reflux for 40 min before the dropwise addition of benzyl bromide (19 mL, 155 mmol, 1.1eq). The mixture was allowed to reflux overnight. Upon completion of the reaction, the solvents were removed under reduced pressure and the residue was taken up in dichloromethane (200 mL) and washed with aqueous solution of sodium hydroxide (5%, 2 x 100 mL). The organic extracts were then washed with water, brine, dried over magnesium sulphate and concentrated under reduced pressure to give the crude product as yellowish crystals. The crude product was recrystallized from isopropanol, dried overnight at 65 °C affording the pure product **(compound 7)** as white crystals (29 g, 125 mmol, 87%). Mp: 126-129°C [lit: 128-130 °C] [1].^{1}H-NMR (400 MHz, DMSO-d_6): δ_H= 4.24 (d, J= 5.6 Hz, 2H, H-6), 4.89 (s, 2H, H-8), 5.70 (t, J= 5.6, 1H, H-7) 6.29 (s, 1H, H-4), 7.26-7.38 (m, 5H, H-10, H-11,

H-12), 8.13 (s, 1H, H-4) ppm; ^{13}C-NMR (100 MHz, DMSO-d_6): δ_C= 65.5 (C-6), 71.0 (C-7), 111.6 (C-1), 128.6 (Ar), 128.7 (Ar), 128.9 (Ar), 136.6 (C-8), 141.6 (C-4), 147.1 (C-3), 168.7 (C-3), 173.8 (C-2) ppm.

[5-(benzyloxy)-4-oxo-4*H*-pyran-2-yl]methyl 4-methylbenzene-1-sulfonate (compound 8) [2]

A sample of 5-(benzyloxy)-2-(hydroxymethyl)-4*H*-pyran-4-one (**compound 7**) (25 g, 108 mmol, 1eq) was dissolved in acetone (350 mL) and stirred vigorously before tosyl chloride (21 g, 110 mmol, 1.1eq) was added at RT. Then, a solution of sodium hydroxide (4.3 g, 108 mmol, 1eq) in water (18 mL) was added and the resulting mixture stirred at RT for 20 min. The crude product was precipitated upon addition of water (150 mL) and was purified by recrystallization from methanol/water affording the pure compound (**compound 8**) as pale-yellow crystals (38 g, 98 mmol, 94%). Mp: 111-114°C [lit: 112°C] [2]. ^{1}H-NMR (400 MHz, DMSO-d_6): δ_H= 2.47 (s, 3H, H-16), 4.77(s, 2H, H-6), 5.02 (s, 2H, H-7), 6.33 (s, 1H, H-1), 7.32-7.36 (m, 7H, H-9, H-10, H-11, H-14), 7.46 (s, 1H, H-1), 7.77 (d, J = 8 Hz, 2H, H-13) ppm; ^{13}C-NMR (100 MHz, DMSO-d_6): δ_C= 21.8 (C-15), 66.0 (C-6), 71.9 (C-7), 115.5 (C-1), 127.8 (Ar), 128.1 (Ar), 128.6 (Ar), 128.9 (Ar), 130.2 (Ar), 132.3 (Ar), 135.5 (Ar), 141.5 (C-4), 145.8 (Ar), 147.4 (C-3), 158.7 (C-5), 174.0 (C-2) ppm. HRMS (ESI) for $C_{20}H_{18}O_6S$; Theoretical [M+H]: 387.0824. Measured [M+H]: 387.0890.

1,3-diethyl 2-{[5-(benzyloxy)-4-oxo-4*H*-pyran-2-yl]methyl}-2-acetamidopropane dioate (compound 9)

In a solution of diethyl acetamidomalonate (10 g, 46 mmol, 2.1eq) in dry *N, N*-dimethyl formamide (70 mL), under nitrogen atmosphere, sodium hydride (60% in mineral oil, 2 g, 83 mmol, 3.7eq) was added in portions. Upon the evolution of hydrogen gas was ceasing, a sample of [5-(benzyloxy)-4-oxo-4*H*-pyran-2-yl]methyl 4-methylbenzene-1-sulfonate (**compound 8**)

(8.5 g, 22 mmol, 1eq) was added to the solution mixture which was stirred overnight at RT and protected from moisture. Upon completion of the reaction, the solvents were removed under reduced pressure forming a brown slurry which was mixed with water (100 mL) and stirred vigorously. The resulting crude product precipitated as brown solid, collected by filtration, left to dry overnight and purified by recrystallization (acetone/petroleum ether 60:80) affording the pure compound (**compound 9**) as light orange crystals (9.21 g, 21 mmol, 95%). Mp: 117-120°C [lit: 117-118°C] [1]. ^{1}H-NMR (400 MHz, DMSO-d_6): δ_H = 1.18 (t, J= 7.2 Hz, 6H, H-10), 1.93 (s, 3H, H-13), 3.41 (s, 2H, H-6), 4.14 (m, 4H, H-9), 4.90 (s, 2H, H-14), 6.10 (s, 1H, H-1), 7.37-7.44 (m, 5H, H-16, H-17, H-18), 8.15 (s, 1H, H-4), 8.51 (s, 1H, H-11) ppm; ^{13}C-NMR (100 MHz, DMSO-d_6): δ_C= 14.3 (C-10), 22.4 (C-13), 31.2 (C-6), 62.8 (C-9), 65.6 (C-7), 71.0 (C-14), 116.1 (C-4), 128.7 (Ar), 128.8 (Ar), 129.0 (Ar), 136.5 (Ar), 142.0 (C-1), 147.2 (C-3), 163.2 (C-5), 167.0 (C-12), 170.4 (C-8), 173.4 (C-2) ppm. HRMS (ESI) for $C_{22}H_{25}NO_8$; Theoretical [M+H]: 432.1655. Measured [M+H]: 432.1656.

***rac*-2-amino-3-(5-hydroxy-4-oxo-4*H*-pyran-2-yl)propanoic acid (compound 10)**

A solution of concentrated hydrochloric acid (HCl) (40 mL) and 1, 3-diethyl 2-{[5-(benzyloxy)-4-oxo-4*H*-pyran-2-yl] methyl}-2-acetamidopropanedioate (**compound 9**) (5.1 g, 11.84 mmol) was heated at 180°C for 3 hrs. Upon completion of the reaction, the solvents were removed under reduced pressure forming a brown solid which was dissolved in water (20 mL). The solution was treated with charcoal, filtered and the pH of the filtrate was adjusted to 5.0 by the dropwise addition of concentrated ammonium hydroxide. The resulting solution was kept overnight at 5°C. White crystals were precipitated, collected, washed with water, acetone, petrol ether (60:80) and dried in the air affording the pure compound (**compound 10**) as white crystals (2.10 g, 10.54 mmol, 89%). Mp: 116-117°C. ^{1}H-NMR (400 MHz, D_2O/CF_3COOD 8:2): δ_H = 2.63-2.75 (m, 2H, H-6), 3.84 (t, J= 6.8 Hz, 1H, H-7), 5.97 (s, 1H, H-1), 7.45 (1H, s, H-4) ppm; ^{13}C-NMR (100 MHz, D_2O/CF_3COOD 8:2): δ_C= 32.9 (C-6), 50.0 (C-7), 116.4 (C-

1), 119.3 (C-4), 142.5 (C-3), 144.2 (C-5), 169.4 (C-8), 175.5 (C-2) ppm. HRMS (ESI) for $C_8H_9NO_5$; Theoretical [M+H]: 199.0713. Measured [M+H]:199.0710.

***rac*-2-amino-3-(5-hydroxy-4-oxo-1,4-dihydropyridin-2-yl)propanoic acid (compound 11)** [1]

A portion of 1,3-diethyl 2-{[5-(benzyloxy)-4-oxo-4*H*-pyran-2-yl]methyl}-2 acetamidopropane dioate (**compound 9**) (4.2 g, 9.73 mmol) was mixed with a solution of concentrated ammonium hydroxide (25 mL) and the mixture was heated for 5 hrs in a stainless-steel bomb at 120°C. Upon completion of the reaction, the mixture was evaporated to dryness and the resulting solid was dissolved in a solution of concentrated HCl (30 mL). The resulting mixture was heated at 180°C for 3 hrs. The solvents were evaporated and the resulting crystals were dissolved in water (20 mL). The solution was treated with charcoal, filtered and the pH was adjusted to 5.0, using ammonia solution. The resulting solution was kept overnight at 5°C forming white crystals which were collected, washed with water, acetone, and light petroleum and dried affording the pure compound (**compound 11**) as white crystals (1.76 g, 8.9 mmol, 91%) Mp: 230-234°C [lit: >250°C] [1]. ^{1}H-NMR (400 MHz, D_2O/CF_3COOD 8:2): δ_H= 2.69-2.83 (m, 2H, H-6), 3.73 (t, J= 6.8 Hz, 1H, H-7), 6.53 (s, 1H, H-1), 7.3 (s, 1H, H-4) ppm; ^{13}C-NMR (100 MHz, D_2O/CF_3COOD): δ_C= 30.1 (C-6), 51.2 (C-7), 113.6 (C-1), 116.4 (C-4), 142.5 (C-3), 143.4 (C-5), 168.7 (C-8), 169.0 (C-2) ppm. HRMS (ESI) for $C_8H_{10}N_2O4$; Theoretical [M+H]: 198.0640. Measured [M+H]: 198.0870.

Synthesis of *rac*-18

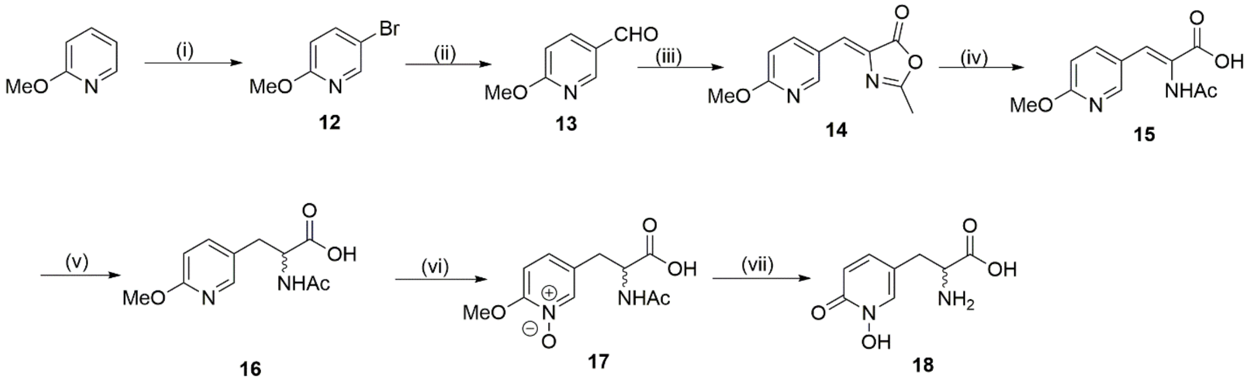

Scheme 2: Reagents and conditions for the synthesis of **compound 18**. (**i**) NBS, CH_3CN, 90°C, 2 hrs, 18%; (**ii**) *n*-BuLi, Et_2O, DMF, -35°C, 7 hrs, 67%; (**iii**) *N*-acetyl glycine, AcONa, Ac_2O, 130°C, 4 hrs, 63%;(**iv**) H_2O, reflux, 4 hrs, 81%; (**v**)H_2,10% Pd/ C (*cat*), MeOH, RT, 9 hrs, 53%; (**vi**) *m*-CPBA, DCM, MeOH, RT, 48 hrs, 77%; (**vii**) conc. HCl, reflux, 1hr, 54%.

5-bromo-2-methoxypyridine (compound 12) [3]

In a suspension of 2-methoxypyridine (15 g, 138 mmol, 1eq) in acetonitrile (415 mL), *N*-bromosuccinimide (30 g, 169 mmol, 1.22eq) was added and the resulting mixture was refluxed for 20 hrs. Upon completion of the reaction, as it was indicated by TLC (SiO_2, eluent: Petroleum Ether 60-80: Ethyl acetate, 8:2; UV-light), the mixture was filtered over a pad of silica. The solvents were evaporated, under reduced pressure, affording the crude product as orange oil which was then purified by an automated flash chromatography column (Isolera™ Biotech); R_f= 0.83 (Petrol Ether 60-80: Ethyl acetate, 95:5; UV light) affording intermediate (**compound 12**) as a pale-yellow oil (4.7 g, 25 mmol, 18%). ^{1}H-NMR (400 MHz, $CDCl_3$,) δ_H= 3.90 (s, 3H, H-7), 6.64 (d, J= 8.8 Hz, 1H, H-3), 7.61 (dd, J=2.8 Hz, J= 8.8 Hz, 1H, H-4), 8.18 (d, J= 2.8 Hz, 1H, H-6) ppm; ^{13}C-NMR (100 MHz, $CDCl_3$) δ_C= 53.6 (C-7), 111.6 (C-5), 112.5 (C-3), 140.9 (C-4), 147.5 (C-6), 162.8 (C-2) ppm.

6-methoxypyridine-3-carbaldehyde (compound 13) [3]

In a solution of 5-bromo-2-methoxypyridine (**compound 12**) (4.7 g, 25 mmol, 1eq) in dry diethyl ether (50 mL) and under inert atmosphere, *n*-Butyl Lithium (2.5 M in hexanes, 12 mL, 30 mmol, 1.2eq) was added at -35°C, and stirred until the formation of a brown precipitate.

Then, dry *N, N*-dimethyl formamide (5.4 mL, 2.8 eq) was added dropwise for 5 min. The resulting mixture was stirred at 0°C (~2 hrs), protected from moisture and under inert atmosphere. Upon completion of the reaction, as it was indicated by TLC (SiO_2, eluent Petroleum Ether 60:80: ethyl acetate, 80:20; UV light), the reaction was quenched by aqueous solution of ammonium chloride (5%, 25 mL). The aqueous layer was extracted with dichloromethane (3 x 50 mL). The combined organic extracts were dried over magnesium sulphate and concentrated under reduced pressure, forming the crude product as orange oil. The crude product was purified by automated flash chromatography column (Isolera™ Biotech); R_f = 0.43 (Petrol Ether 60-80: diethyl ether, 60:40; UV light) affording intermediate **(compound 13)** as yellow crystals (2.53 g, 16.7 mmol, 67%). Mp: 42-44°C, [lit: 42-46°C] [3]. ^{1}H-NMR (400 MHz, $CDCl_3$,) δ_H= 4.03 (s, 3H, H-9), 6.84 (d, J= 8.4 Hz, 1H, H-3), 8.05 (dd, J= 2.4 Hz, J= 8.4 Hz, 1H, H-4), 8.63 (d, J= 2.4 Hz, 1H, H-1), 9.96 (s, 1H, H-8) ppm; ^{13}C-NMR (100 MHz, $CDCl_3$) δ_C= 54.4 (C-9), 112.2 (C-4), 126.7 (C-6), 137.5 (C-5) 153.5 (C-3), 167.8 (C-2), 189.6 (C-7) ppm.

(4*Z*)-4-[(6-methoxypyridin-3-yl)methylidene]-2-methyl-4,5-dihydro-1,3-oxazol-5-one (compound 14)

The synthesis was performed according to a modified method previously published [4]. Briefly, in a solution of 6-methoxypyridine-3-carbaldehyde (**compound 13**) (1.84 g, 12.17 mmol, 1eq) in acetic anhydride (8 mL), a sample of *N*-acetyl glycine (2.04 g, 17.44 mmol, 1.5eq.) and a sample of sodium acetate (1.5 g, 18.29 mmol, 1.5eq) were added sequentially. The resulting mixture was stirred at 125°C for 4 hrs. Upon completion of the reaction, the mixture was poured into ice-water and stirred for a further 1 hr leading to the formation of a yellow solid of the crude product which was collected by vacuum filtration, washed with water and dried in air. The crude product was purified by recrystallization from methanol affording the pure product (**compound 14**) as pale-yellow solid (1.66 g, 7.6 mmol, 63%). Mp: 152-154°C. ^{1}H-NMR (400

MHz, $CDCl_3$,) δ_H= 2.40 (s, 3H, H-11), 4.00 (s, 3H, H-12), 6.83 (d, J= 8.8 Hz, 1H, H-3), 7.12 (s, 1H, H-7), 8.6 (d, J= 2.4 Hz, H-6), 8.67 (dd, J= 2.4 Hz, J= 8.8 Hz. H-4) ppm; ^{13}C-NMR (100 MHz, $CDCl_3$,) δ_C= 15.8 (C-11), 54.1 (C-12), 111.8 (C-3), 123.3 (C-7), 128.1 (C-5), 132.0 (C-8), 140.9 (C-4), 152.1 (C-6), 165.4 (C-10), 165.7 (C-9), 167.7 (C-2) ppm. HRMS (ESI) for $C_{11}H_{10}N_2O_3$; Theoretical [M+H]: 219.0691. Measured [M+H]: 219.0766.

(2*Z*)-2-acetamido-3-(6-methoxypyridin-3-yl)prop-2-enoic acid (compound 15)

The synthesis was performed according to a modified method previously published [4]. Briefly, a sample of (4*Z*)-4-[(6-methoxypyridin-3-yl)methylidene]-2-methyl-4,5-dihydro-1,3-oxazol-5-one (**compound 14**) (2 g, 9.16 mmol) was dissolved in a mixture of water (30 mL)/acetone (50 mL) and the resulting mixture was refluxed for 9 hrs. The solution was allowed to cool down to RT and then was concentrated, under reduced pressure, forming the crude product as yellow solid. The crude product was purified by recrystallization from methanol affording **compound 15** as pale brown crystals (1.76 g, 7.45 mmol, 81%). Mp: 162-164°C. ^{1}H-NMR (400 MHz, DMSO-d_6,) δ_H= 2.00 (s, 3H, H-10), 3.89 (s, 3H, H-13), 6.88 (d, J= 8.8 Hz, 1H, H-3), 7.25 (s, 1H, H-7), 7.98 (dd, J= 2.4 Hz, J= 8.8 Hz, 1H, H-4), 8.40 (d, J= 2.4 Hz, 1H, H-6), 9.48 (s, 1H, H-12) ppm; ^{13}C-NMR (100 MHz, DMSO-d_6) δ_C= 23.2 (C-10), 54.0 (C-13), 111.1 (C-3), 124.1 (C-7), 127.1 (C-5), 128.7 (C-8), 139.8 (C-4), 149.7 (C-6), 164.1 (C-10), 166.8 (C-9), 169.7 (C-2) ppm. HRMS (ESI) for $C_{11}H_{12}N_2O_4$; Theoretical [M+H]: 237.0797. Measured [M+H]: 237.0871.

***rac*-2-acetamido-3-(6-methoxypyridin-3-yl)propanoic acid (compound 16)**

In a suspension of (2*Z*)-2-acetamido-3-(6-methoxypyridin-3-yl)prop-2-enoic acid (**compound 15**) (1.76 g, 7.45 mmol) in methanol (50 mL), Pd/C (10%) was added. The reaction mixture was stirred under hydrogen gas at RT for 9 hrs. Upon completion of the reaction, the solution mixture was filtrated over a pad of Celite forming a yellow oil which was concentrated under reduced pressure. The resulting slurry was purified by automated flash chromatography column

(Isolera^{TM} Biotech) (SiO_2); R_f = 0.89 (dichloromethane: methanol 9:1; UV light) affording compound **compound 16** as a pale-yellow oil which was solidified on standing (940 mg, 3.94 mmol, 53%). Mp: 181-183°C. ^{1}H-NMR (400 MHz, CD_3OD) δ_H= 2.01 (3H, s, H-10), 2.95-2.30 (1H, m, H-7), 3.20-3.25 (m, 1H, H-7), 3.95 (s, 3H, H-13), 4.70-4.73 (m, 1H, H-8), 6.83 (d, J= 8.4 Hz, 1H, H-3), 7.67 (dd, J= 2.4 Hz, J= 8.4 Hz, 1H, H-4), 8.15 (d, J= 2.4 Hz, 1H, H-6) ppm; ^{13}C-NMR (100 MHz, CD_3OD) δ_C= 21.6 (C-10), 34.1 (C-7), 53.4 (C-13), 54.1 (C-8), 110.7 (C-3), 126.4 (C-4), 140.7 (C-5), 147.2 (C-6), 164.0 (C-2), 172.5 (C-11), 173.7 (C-9) ppm. HRMS (ESI) for $C_{11}H_{14}N_2O_4$; Theoretical [M+H]: 238.0953. Measured [M+H]: 238.0961.

***rac*-5-(2-carboxy-2-acetamidoethyl)-2-methoxypyridin-1-ium-1-olate (compound 17)**

In a suspension of 2-acetamido-3-(6-methoxypyridin-3-yl)propanoic acid (**compound 16**) (560 mg, 2.35 mmol, 1eq) in a solution mixture of dichloromethane/methanol (9:1, 30mL) a sample of m-chloroperoxy benzoic acid (1 g, 5.8 mmol, 2.5eq) was added. The resulting mixture was stirred at RT for 48 hrs under nitrogen atmosphere. Upon completion of the reaction, the solvents were removed under reduced pressure and the resulting yellowish slurry residue was washed several times with diethyl ether. Filtration of the product leaded to isolation of the title compound as a white powder (460 mg, 1.80 mmol, 77%). Mp: 189-192°C. ^{1}H-NMR (400 MHz, DMSO-*d6*) δ_H= 1.79 (s, 3H, H-10), 2.71-2.77 (m 1H, H-7), 2.95-2.99 (m, 1H, H-7), 3.93 (s, 3H, H-13), 4.38-4.43 (m, 1H, H-8), 7.13 (d, J= 8.7 Hz, 1H, H-12), 7.23 (dd, J= 2.0 Hz, J= 8.7 Hz, 1H, H-3), 8.11 (d, J= 2.0 Hz, 1H, H-6), 8.23 (1H, d, J=8.7 Hz, H-4) ppm; ^{13}C-NMR (100 MHz, DMSO-*d6*) δ_C= 22.8 (C-10), 33.2 (C-7), 53.2 (C-8), 57.5 (C-13), 109.0 (C-3), 128.1 (C-6), 128.3 (C-5), 139.9 (C-4), 157.5 (C-2), 169.8 (C-11), 173.2 (C-9) ppm. HRMS (ESI) for $C_{11}H_{14}N_2O_5$; Theoretical [M+H]: 254.0902. Measured [M+H]: 254.0912.

***rac*- 2-amino-3-(1-hydroxy-6-oxo-1,6-dihydropyridin-3-yl)propanoic acid (compound 18)**

A sample of *rac*-5-(2-carboxy-2-acetamidoethyl)-2-methoxypyridin-1-ium-1-olate (**compound 17**) (460 mg, 1.80 mmol) was dissolved in concentrated solution of HCl (20 mL).

The resulting mixture was refluxed for 3 hrs. Upon completion of the reaction, the mixture was concentrated to dryness leading to the formation of brownish crystals the target molecule (300 mg, 1.51 mmol, 84%). Mp: 120-122°C. ^{1}H-NMR (400 MHz, D_2O/CF_3COOD 8:2) δ_H= 2.96-3.10 (2H, m, H-7), 4.13 (d, J= 4.8 Hz, 1H, H-8), 6.63 (t, J= 9.2 Hz, 1H, H-4), 7.43 (d, J= 9.2 Hz, 1H, H-3), 7.94 (s, 1H, H-6), 8.49 (s, 3H, H-10) ppm; ^{13}C-NMR (100 MHz, D_2O/CF_3COOD 8:2) δ_C= 31.4 (C-7), 52.9 (C-8), 118.9 (C-3), 136.6 (C-5), 140.7 (C-6), 140.8 (C-4), 157.8 (C-2), 170.4 (C-9) ppm. A small portion of the titled molecule was dissolved in water and basified (pH 5.0) with ammonium hydroxide solution and kept at 5°C for several weeks until precipitation. HRMS (ESI) for $C_8H_{10}N_2O_4$; Theoretical [M+H]: 199.0713. Measured [M+H]: 199.0711.

Synthesis of *L*-22 and *D*-23

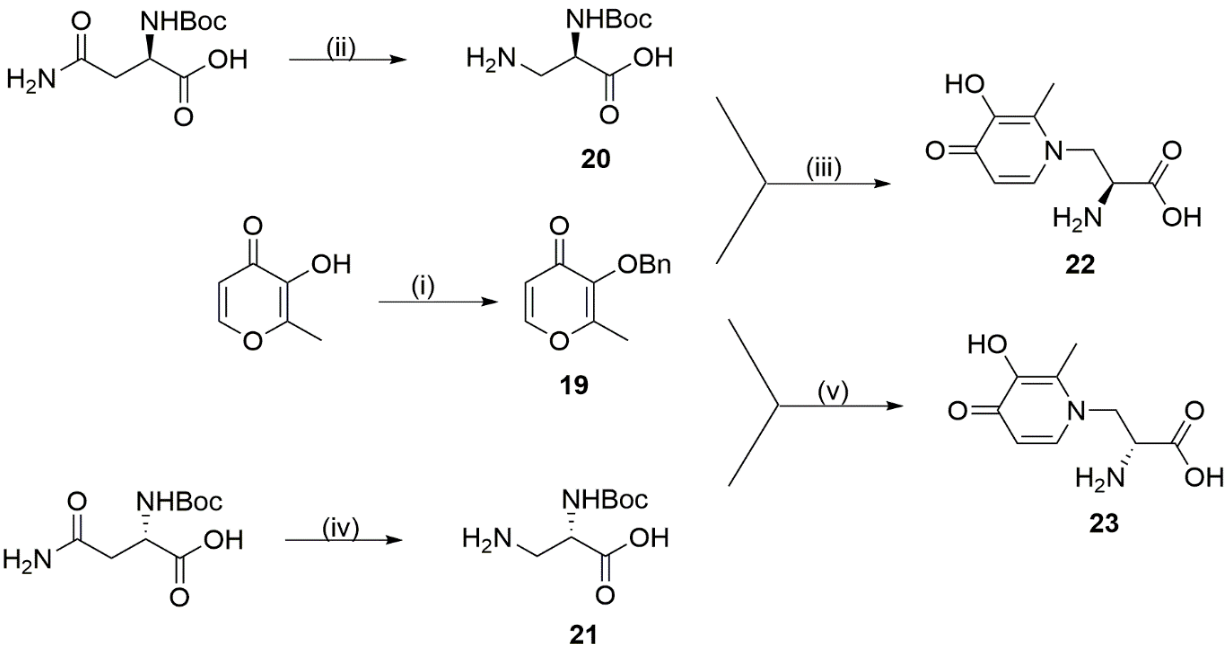

Scheme 3: Reagents and conditions for the synthesis of **compounds 22 and 23**. (i)BnBr, K_2CO_3, DMF, 80°C, 1 hr, 76%; (**ii**) **a**) *N*-Boc-L-Asn, Iodosobenzene diacetate, EtOAc: MeCN: H_2O, RT, 4hrs, 76%; **iii**) **a**) EtOH: H_2O, 8 days, **b)** conc. HBr, reflux, 20 mins, **c**) NH_3, pH 5, 5°C/,72 hrs, 63%. Same reagents and conditions for step *iv* (90%) and *v* (47%).

3-(benzyloxy)-2-methyl-1,4-dihydropyridin-4-one (compound 19) [7]

In a solution of maltol (10 g, 79.26 mmol, 1eq) in *N, N*- dimethyl formamide (100 mL), benzyl bromide (9.42 mL, 79.26 mmol, 1eq) was added and the solution mixture was stirred at 80°C for 15 min. Then, a sample of potassium carbonate (12.05 g, 87.18 mmol, 1.1eq) was added to the reaction mixture and the final mixture was heated at 80°C for a further 1 hr. Upon completion of the reaction, the excess of inorganic salt was removed by filtration and the filtrates were concentrated under reduced pressure. The resulting residue was dissolved in tetrahydrofuran (50mL) and any remaining of the inorganic salt was removed by filtration. Then, the filtrates were concentrated under reduced pressure affording the titled compound as a viscous orange oil (16.28 g, 75.29 mmol, 95%). ^{1}H-NMR (400 MHz, $CDCl_3$) δ_H= 2.07 (s, 3H, H-6), 5.13 (s, 2H, H-7), 6.37 (d, J= 5.6 Hz, 1H, H-1), 7.28-7.39 (m, 5H, H-9, H-10, H-11), 7.60 (d, J= 5.6 Hz, 1H, H-2) ppm; ^{13}C-NMR (100 MHz, $CDCl_3$) δ_C= 14.6 (C-6), 73.3 (C-7),

116.8 (C-1), 128.2 (Ar), 128.3 (Ar), 128.8 (Ar), 136.7 (Ar), 143.6 (C-4), 153.9 (C-2), 159.8 (C-3), 175.02 (C-5) ppm.

(2*L*)-3-amino-2-{[(tert-butoxy)carbonyl]amino}propanoic acid (compound 20) [5]

In a solution mixture composed of ethyl acetate (24 mL), acetonitrile (24 mL) and water (12 mL), *N*-Boc-*L*-asparagine (5.0 g, 21.5 mmol, 1eq) and iodosobenzene diacetate (8.32 g, 25.8 mmol, 1.2eq) were added. The resulting slurry, was stirred at 16°C for 30 min and then at 20°C for 4 hrs. Upon completion of the reaction, the mixture was cooled at 0°C for 15 min forming a white salt which was collected by filtration. The filter-cake was then washed with cooled ethyl acetate (30 mL) affording compound 20 as a white solid (3.34 g, 16.35 mmol, 76%). Mp. 203-207°C [lit: 207-212°C] [5]. ^{1}H-NMR (400 MHz, DMSO-d_6) δ_H= 1.38 (s, 9H, H-6), 2.67-2.73 (m, 1H, H-3), 2.99-3.02 (m, 1H, H-3), 3.57-3.61 (m, 2H, H-2), 6.16 (s, br, 1H, H-7) ppm; ^{13}C-NMR (100 MHz, DMSO-d_6) δ_C= 28.6 (C-6), 41.2 (C-3), 51.3 (C-2), 78.7 (C-5), 155.6 (C-4), 171.6 (C-1) ppm.

(2*D*)-3-amino-2-{[(tert-butoxy)carbonyl]amino} propanoic acid (compound 21) [5]

Same procedure as for (2*L*)-3-amino-2-{[(tert-butoxy)carbonyl]amino}propanoic acid (**compound 20**) was followed. White crystals (3.94 g, 19.3 mmol, 90%). Mp: 200-204°C [lit: 207-209°C] [5]. ^{1}H-NMR (400 MHz, DMSO-d_6) δ_H= 1.39 (s, 9H, H-6), 2.68-2.74 (m, 1H, H-3), 2.99-3.03 (m, 1H, H-3), 3.57-3.62 (m, 2H, H-2), 6.15 (s, br, 1H, H-7) ppm; ^{13}C-NMR (100 MHz, DMSO-d_6) δ_C= 28.5 (C-6), 41.4 (C-3), 51.6 (C-2), 78.6 (C-5), 155.6 (C-4), 171.6 (C-1) ppm.

(2*L*)-amino-3-(3-hydroxy-2-methyl-4-oxo-1,4-dihydropyridin-1-yl)propanoic acid (compound 22)

The synthesis was performed according to a modified method previously published [1]. A sample of 3-(benzyloxy)-2-methyl-1,4-dihydropyridin-4-one (**compound 19**) (4.9 g, 22.66 mmol, 1eq) was mixed with a sample of (2*L*)-2-amino-2-{[(tert-butoxy)carbonyl]amino}acetic

acid (**compound 20**) (2.9 g, 14.2 mmol, 0.6eq.) and dissolved in water (100 mL) and ethanol (100 mL) containing sodium hydroxide (2 g, 50 mmol). The resulting solution was allowed to stir at RT for 8 days. Then, the solution was acidified to pH 2.0 by the addition of concentrated HCl. The excess of solvents was removed under reduced pressure. The resulting residue was mixed with hydrobromic acid (48% w/v, 20 mL) and refluxed for 20 min. The solution mixture was concentrated under reduced pressure, and then the resulting solid was dissolved in water (20 mL), treated with charcoal and basified (pH 5.0) by the addition of ammonium hydroxide solution. The resulting solution was cooled to 5°C for 72 hrs, where brown crystals were precipitated. The crystals were collected washed with excess of water and dried on air affording the titled compound as pale brown crystals (3.23 g, 14.27 mmol, 63%). Mp: 165-168°C. ^{1}H-NMR (400 MHz, D_2O/CF_3COOD) δ_H= 1.90 (s, 3H, H-6), 3.85 (t, J= 7.2 Hz, 1H, H-8), 4.07-4.12 (m, 1H, H-7), 4.27-4.33 (m, 1H, H-7), 6.44 (d, J= 8 Hz, 1H, H-2), 7.36 (d, J= 8 Hz, 1H, H-1) ppm; ^{13}C-NMR (100 MHz, D_2O/CF_3COOD) δ_C= 13.4 (C-6), 49.17 (C-7), 52.24 (C-8), 119.54 (C-2), 141.59 (C-1), 143.28 (C-5), 147.33 (C-4), 170.96 (C-8), 176.7 (C-3) ppm. HRMS (ESI) for $C_9H_{12}N_2O_4$; Theoretical [M+H]: 213.0869. Measured [M+H]: 213.0866.

2-amino-3-(3-hydroxy-2-methyl-4-oxo-1,4-dihydropyridin-1-yl)propanoic acid (compound 23)

Same procedure was followed as for the one described for (2*D*)-amino-3-(3-hydroxy-2-methyl-4-oxo-1,4-dihydropyridin-1-yl)propanoic acid (**compound 21**). Pale brown crystals after re-crystallization from water/methanol (1.78 g, 8.43 mmol, 47%). Mp: 163-168°C. ^{1}H-NMR (400 MHz, D_2O/CF_3COOD) δ_H= 1.92 (s, 3H, H-6), 3.91 (t, J= 7.2 Hz, 1H, H-8), 4.23-4.29 (m, 1H, H-7), 4.49-4.55 (m, 1H, H-7), 6.37 (d, J= 8 Hz, 1H, H-2), 7.48 (d, J= 8 Hz, 1H, H-1) ppm; ^{13}C-NMR (100 MHz, D_2O/CF_3COOD) δ_C= 13.4 (C-6), 49.2 (C-7), 52.2 (C-8), 119.54 (C-2), 141.7 (C-1), 143.3 (C-5), 147. (C-4), 170.9 (C-8), 176.7 (C-3) ppm. HRMS (ESI) for $C_9H_{12}N_2O_4$; Theoretical [M+H]: 213.0869. Measured [M+H]: 213.0865

Synthesis of *rac*-29

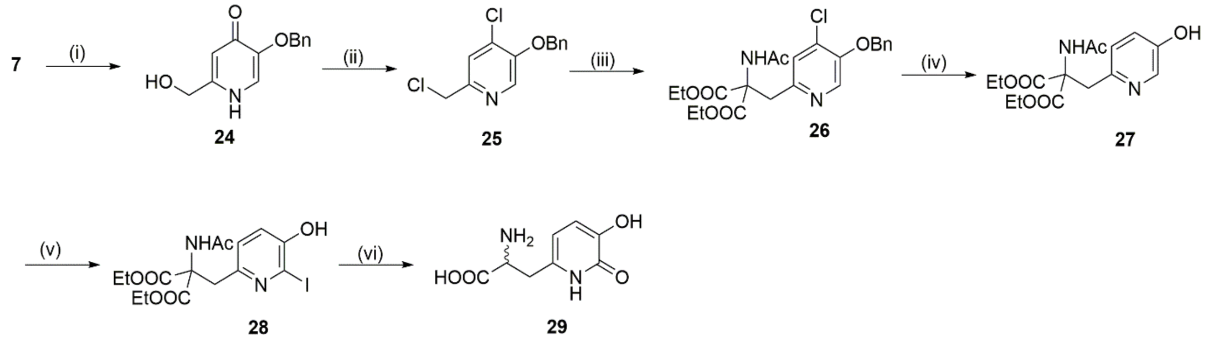

Scheme 4: Reagents and conditions for the synthesis of **compound 29**: (**i**) conc. NH_4OH, 5 hrs, 120°C, (**ii**) $POCl_3$, 40 mins, 120°C, (**iii**) Diethyl acetamidomalonate, NaH (60% in mineral oil), $DMF_{(dry)}$, overnight, RT, (**iv**) H_2, 5% Pd/C, methanol, RT, (**v**) Na_2CO_3 $_{(aq)}$, I_2, KI, overnight, RT, (**vi**) **a**) $Ba(OH)_2$ $_{(aq)}$, 24 hrs, 120°C, 24 hrs **b**) conc. HCl, 180°C, 1 hr, **c**) conc. NH_4OH, pH 5, 5°C.

5-(benzyloxy)-2-(hydroxymethyl)-1,4-dihydropyridin-4-one (compound 25) [6]

In a stainless-steel bomb, concentrated ammonium hydroxide (40 mL) was mixed with a sample of 5-(benzyloxy)-2-(hydroxymethyl)-4*H*-pyran-4-one (**compound 7**) (25 g, 107.64 mmol). The resulting mixture was heated at 120°C for 5 hrs. Upon completion of the reaction, volatiles were removed under reduced pressure. The resulting slurry was extracted with hot acetone, filtrated and washed with excess of hot acetone affording the titled compound as brown crystal (20 g, 86.48 mmol, 80%). M.p. 228-232°C [lit. 230-235°C] [6]. ^{1}H-NMR (400 MHz, DMSO-d_6) δ_H= 4.34 (s, 2H, H-8), 5.00 (s, 2H, H-11), 6.23 (s, 1H, H-1), 7.25-7.35 (m, 6H, H-4, H-13, H-14, H-15) ppm; ^{13}C-NMR (100 MHz, DMSO-d_6) δ_C= 60.3 (C-8), 70.9 (C-11), 112.0 (C-1), 124.0 (C-4), 128.2 (Ar), 128.3 (Ar), 128.4 (Ar), 128.7 (Ar), 128.8 (Ar), 137.8 (Ar), 147.0 (C-2), 149.7 (C-5), 171.6 (C-6) ppm.

5-(benzyloxy)-4-chloro-2-(chloromethyl)pyridine (compound 25) [6]

A sample of 5-(benzyloxy)-2-(hydroxymethyl)-1,4-dihydropyridin-4-one (**compound 24**) (13.84 g, 60 mmol) was added to a suspension of phosphorus oxychloride (42 mL), in portions, thus increasing the temperature of the reaction. After the solution mixture was returned back to RT, it was heated at 150°C for 40 min. Upon completion of the reaction, the mixture was

poured into ice-water and stirred vigorously. Addition of more ice into the stirred mixture enhanced the hydrolysis of phosphorous oxychloride and led to the precipitation of the pure product **(compound 19)** (14.5 g, 60 mmol, 87%) as a black solid which was isolated by filtration and left to dry overnight. Mp: 77-79°C [lit: 80-81°C] [6]. ^{1}H-NMR (400 MHz, DMSO-d_6) δ_H= 4.74 (s, 2H, H-7), 5.38 (s, 2H, H-8), 7.36-7.50 (m, 5H, H-10, H-11, H-12), 7.76 (s, 1H, H-1), 8.53 (s, 1H, H-4) ppm; ^{13}C-NMR (100 MHz, DMSO-d_6) δ_C= 45.4 (C-7), 71.5 (C-8), 125.7 (C-1), 128.2 (Ar), 128.4 (Ar), 128.8 (Ar), 129.1 (C-6), 133.1 (C-2), 135.5 (Ar), 136.2 (C-4), 149.4 (C-5), 150.8 (C-7) ppm.

1,3-diethyl-2-{[5-(benzyloxy)-4-chloropyridin-2-yl]methyl}-2-acetamidopropane dioate (compound 26) [6]

In dry *N,N*- dimethylformamide (62 mL) sodium hydride (60% in mineral oil, 2.16 g, 90 mmol, 1.7eq) was added. The solution was stirred at RT and then diethyl acetamidomalonate (11.25 g, 51.8 mmol, 1.7eq) was added in portion evolving hydrogen gas. Upon ceasing of hydrogen gas evolution, 5-(benzyloxy)-4-chloro-2-(chloromethyl) pyridine (**compound 25**) (13.84 g, 51.6 mmol, 1eq) was added. The resulting solution mixture was stirred overnight at RT. Upon completion of the reaction, acetic acid (25 mL) was added to neutralise the reaction mixture which was then concentrated, under reduced pressure, and the resulting syrup was dissolved in water (200mL) and extracted with diethyl ether (2 x 80 mL). The combined organic extracts were washed with brine, dried over magnesium sulphate and concentrated under reduced pressure, affording the titled compound as white crystals (22.7 g, 50.6 mmol, 98%), which was recrystallized from ethanol. Mp 119-122°C [Lit=118-120°C] [6]. ^{1}H-NMR (400 MHz, DMSO-d_6) δ_H= 1.18 (t, J= 7.2 Hz, 6H, H-12), 1.82 (s, 3H, H-9), 3.50 (s, 2H, H-6), 4.08 (q, J= 7.2 Hz, J= 14.4 Hz, 4H, H-11), 5.25 (s, 2H, H-13), 7.13 (1H, s, H-8), 7.30-7.44 (m, 5H, H-15, H-16, H-17), 8.02 (s, 1H, H-5), 8.34 (s. 1H, H-3) ppm; ^{13}C-NMR (100 MHz, DMSO-d_6) δ_C= 14.3 (C-12), 22.5 (C-9), 40.1 (C-6), 62.3 (C-11), 66.6 (C-13), 71.1 (C-7), 125.8 (C-5), 128.2 (C-1),

128.7 (Ar), 129.1 (Ar), 131.3 (Ar), 136.0 (Ar), 136.6 (C-3), 149.6 (C-2), 149.9 (C-4), 167.5 (C-10), 169.9 (C-8) ppm.

1,3-diethyl 2-acetamido-2-[(5-hydroxypyridin-2-yl)methyl]propanedioate (compound 27) [6]

A suspension of diethyl 2-{[5-(benzyloxy)-4-chloropyridin-2-yl]methyl}-2-acetamidopropanedioate (**compound 26**) (6 g, 13.36 mmol, 1eq) in methanol (75 mL), sodium acetate (6 g, 73.14 mmol, 5.5eq) and a catalytic amount of 10% Pd/C was stirred vigorously under hydrogen atmosphere. Upon completion of the reaction, as indicated by TLC (SiO_2, ethyl acetate 100%), the solution mixture was filtered over a pad of Celite, washed with methanol and diluted with water (150 mL) forming the titled compound as white crystals (2.94 g, 9.06 mmol, 68%) Mp: 152-154°C [Lit: 150-153°C] [6] ^{1}H-NMR (400 MHz, DMSO-d_6) δ_H= 1.1 (t, J= 7.2 Hz, 6H, H-12), 1.81 (s, 3H, H-9), 3.43 (s, 2H, H-6), 4.07 (q, J= 7.2 Hz, 4H, H-11), 6.78 (d, J= 8.8 Hz, 1H, H-5), 6.99 (dd, J= 3.2 Hz, J= 8.8 Hz, 1H, H-1), 7.87 (s, 1H, H-13), 7.91 (d, J= 3.2 Hz, 1H, H-3) ppm; ^{13}C-NMR (100 MHz, DMSO-d_6) δ_C= 14.3 (C-12), 22.6 (C-9), 39.9 (C-6), 66.8 (C-11), 69.0 (C-7) 122.8 (Ar), 125.1 (Ar), 137.6 (Ar), 146.5 (Ar), 152.8 (Ar), 167.7 (C-10), 169.7 (C-8) ppm.

1,3-diethyl-2-acetamido-2-[(5-hydroxy-6-iodopyridin-2-yl)methyl] propanedioate (compound 28) [6]

A sample of diethyl 2-acetamido-2-[(5-hydroxypyridin-2-yl)methyl] propanedioate (2.5 g, 7.7 mmol, 1eq) (**compound 27**) was dissolved in water (70 mL) containing sodium carbonate (1.54 g, 13.97 mmol, 1.8eq). A solution of iodine (1.92 g, 15.13 mmol, 2eq) and potassium iodide (2.32 g, 13.97 mmol) in water (50 mL) was added dropwise to the previous solution. The resulting mixture was stirred overnight at RT. Upon completion of the reaction, the solution mixture was neutralised with glacial acetic acid (5 mL), leading to the precipitation of the titled compound which was collected by filtration, washed with water and dried at 90°C. The titled

compound (**compound 28**) was obtained as a white powder (3.39 g, 7.54 mmol, 98%) M.p: 200-203°C. [Lit: 196-197°C] [6]. ^{1}H-NMR (400 MHz, DMSO-d_6) δ_H= 1.15 (t, J= 7.2 Hz, 6H, H-13), 1.83 (s, 3H, H-10), 3.34 (s, 2H, H-6), 4.11 (q, J= 7.2 Hz, 4H, H-13), 6.78 (d, J= 8 Hz, 1H, H-5), 6.98 (d, J= 8 Hz, 1H, H-1), 7.9 (s, 1H, H-8) ppm; ^{13}C-NMR (100 MHz, DMSO-d_6) δ_C= 14.4 (C-13), 22.5 (C-10), 39.0 (C-6), 62.2 (C-11), 66.8 (C-7), 111.1 (Ar), 121.7 (Ar), 125.1 (Ar), 148.0 (Ar), 153.2 (Ar), 167.5 (C-9), 169.9 (C-11) ppm.

rac-2-amino-3-(5-hydroxy-6-oxo-1,6-dihydropyridin-2-yl)propanoic acid (compound 29) [6]

A mixture of barium hydroxide (7.1 g, 41.43 mmol, 4.7eq) and diethyl 2-acetamido-2-[(5-hydroxy-6-iodopyridin-2-yl)methyl] propanedioate (**compound 28**) (4 g, 8.88 mmol, 1eq) in water (70 mL) was refluxed for 24 hrs. Upon completion of the reaction, the resulting barium salt was collected and refluxed with concentrated HCl (50 mL) for 1 hr. Once again, upon completion of the reaction, the solution mixture was evaporated to dryness yielding a yellowish salt of the crude product which was dissolved in water (20 mL), treated with charcoal and filtered. The pH of the filtrates was adjusted to 5.0 by addition of concentrated ammonia leading to precipitation of the titled compound appearing as white crystals (1.67 g, 8.44 mmol, 95%) M.p: 193-195°C. [Lit: 196-197°C] [6]. ^{1}H-NMR (400 MHz, DMSO-d_6) δ_H= 2.86-2.99 (m, 2H, H-6), 4.07 (t, J= 7.6 Hz, 1H, H-7), 6.06 (d, J= 7.2 Hz, 1H, H-5), 6.75 (d, J= 7.2 Hz, 1H, H-1) ppm; ^{13}C-NMR (100 MHz, DMSO-d_6) δ_C= 32.4 (C-6), 52.0 (C-7), 110.8 (C-5), 121.2 (C-1), 131.3 (C-2), 144.7 (C-4), 159.0 (C-3), 170.3 (C-8) ppm. HRMS (ESI) for $C_9H_{10}N_2O_4$; Theoretical [M+H]: 213.0869. Measured [M+H]: 213.0866.

PROOF OF PRODUCTS PURITY BY HPLC

Method: 100% H_2O with 5% ACN isocratic for 20 mins with flowrate of 1 $mL.min^{-1}$. Sample injection volume: 50 µL. Column temperature: 28 ºC

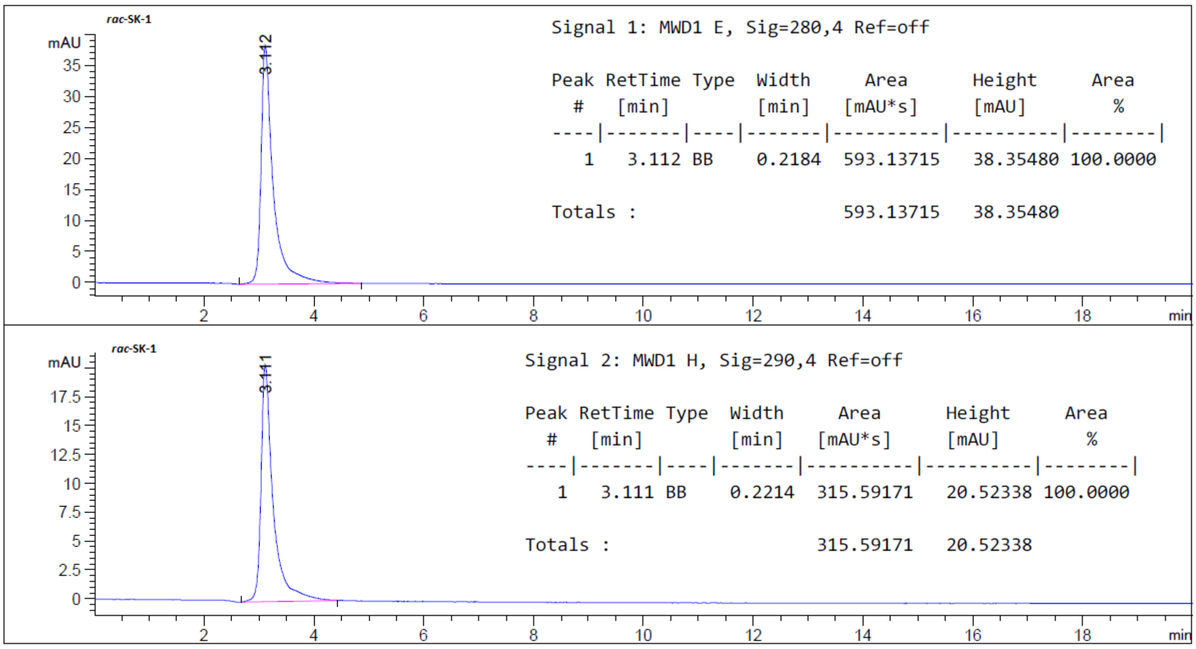

Figure S1: Analytical HPLC chromatogram of compound 10

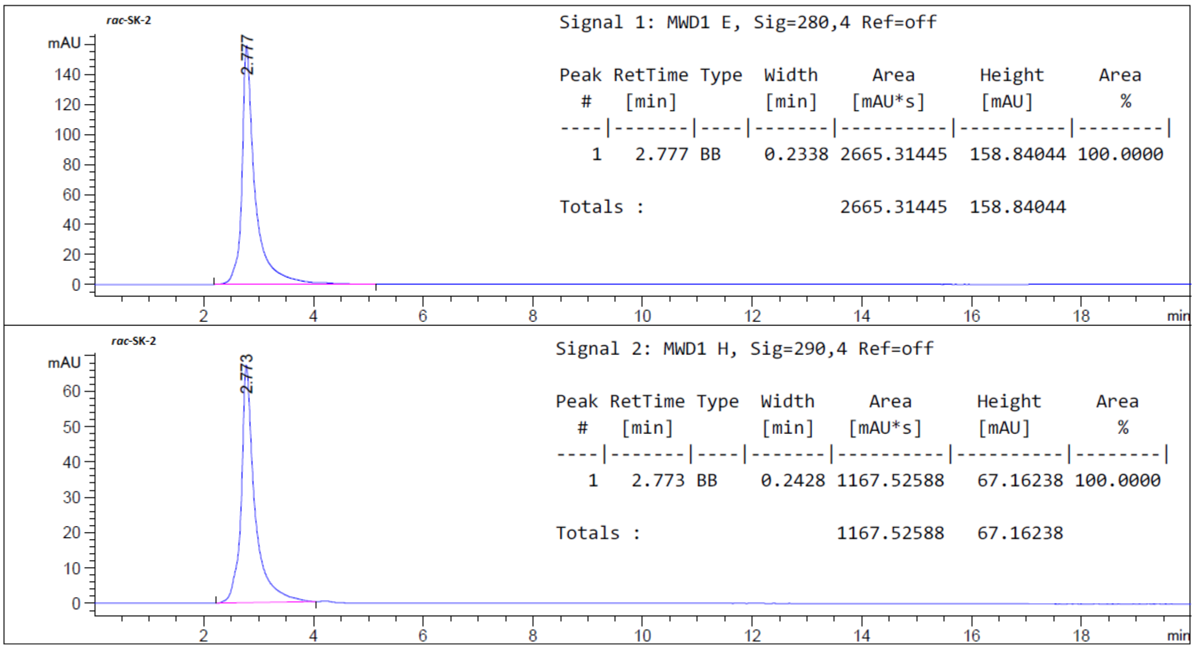

Figure S2: Analytical HPLC chromatogram of compound 11

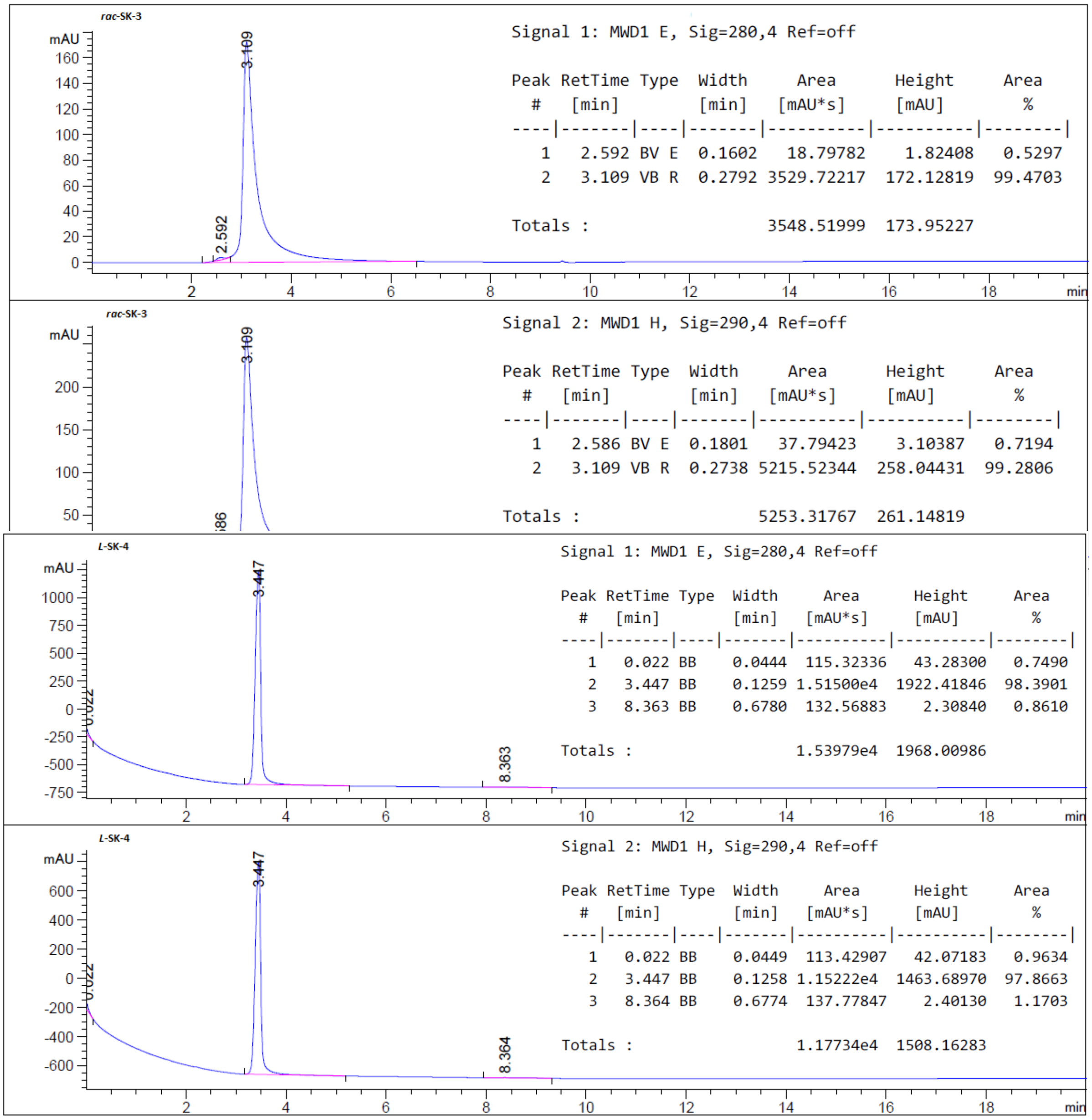

rac-SK-3 — Signal 1: MWD1 E, Sig=280,4 Ref=off

Peak #	RetTime [min]	Type	Width [min]	Area [mAU*s]	Height [mAU]	Area %
1	2.592	BV E	0.1602	18.79782	1.82408	0.5297
2	3.109	VB R	0.2792	3529.72217	172.12819	99.4703
Totals :				3548.51999	173.95227	

rac-SK-3 — Signal 2: MWD1 H, Sig=290,4 Ref=off

Peak #	RetTime [min]	Type	Width [min]	Area [mAU*s]	Height [mAU]	Area %
1	2.586	BV E	0.1801	37.79423	3.10387	0.7194
2	3.109	VB R	0.2738	5215.52344	258.04431	99.2806
Totals :				5253.31767	261.14819	

L-SK-4 — Signal 1: MWD1 E, Sig=280,4 Ref=off

Peak #	RetTime [min]	Type	Width [min]	Area [mAU*s]	Height [mAU]	Area %
1	0.022	BB	0.0444	115.32336	43.28300	0.7490
2	3.447	BB	0.1259	1.51500e4	1922.41846	98.3901
3	8.363	BB	0.6780	132.56883	2.30840	0.8610
Totals :				1.53979e4	1968.00986	

L-SK-4 — Signal 2: MWD1 H, Sig=290,4 Ref=off

Peak #	RetTime [min]	Type	Width [min]	Area [mAU*s]	Height [mAU]	Area %
1	0.022	BB	0.0449	113.42907	42.07183	0.9634
2	3.447	BB	0.1258	1.15222e4	1463.68970	97.8663
3	8.364	BB	0.6774	137.77847	2.40130	1.1703
Totals :				1.17734e4	1508.16283	

Figure S4: Analytical HPLC chromatogram of compound 22

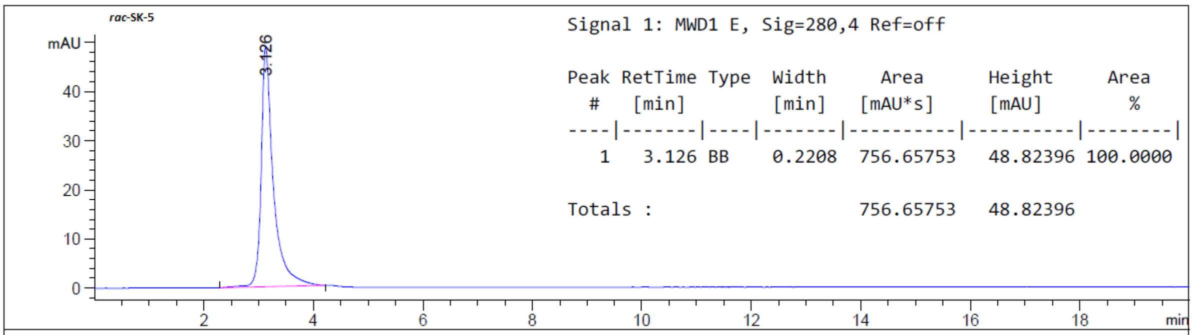

Signal 1: MWD1 E, Sig=280,4 Ref=off

Peak #	RetTime [min]	Type	Width [min]	Area [mAU*s]	Height [mAU]	Area %
1	3.126	BB	0.2208	756.65753	48.82396	100.0000
Totals :				756.65753	48.82396	

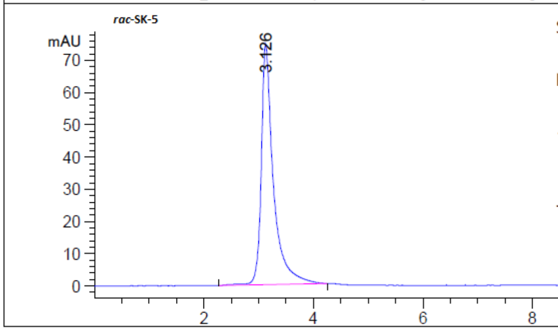

Signal 2: MWD1 H, Sig=290,4 Ref=off

Peak #	RetTime [min]	Type	Width [min]	Area [mAU*s]	Height [mAU]	Area %
1	3.126	BB	0.2198	1150.25171	74.65567	100.0000
Totals :				1150.25171	74.65567	

Figure S5: Analytical HPLC chromatogram of compound 29

^{1}H AND ^{13}C-NMR FOR ALL INTERMEDIATES AND FINAL PRODUCTS; HRMS FOR NOVEL COMPOUNDS AND FINAL PRODUCTS

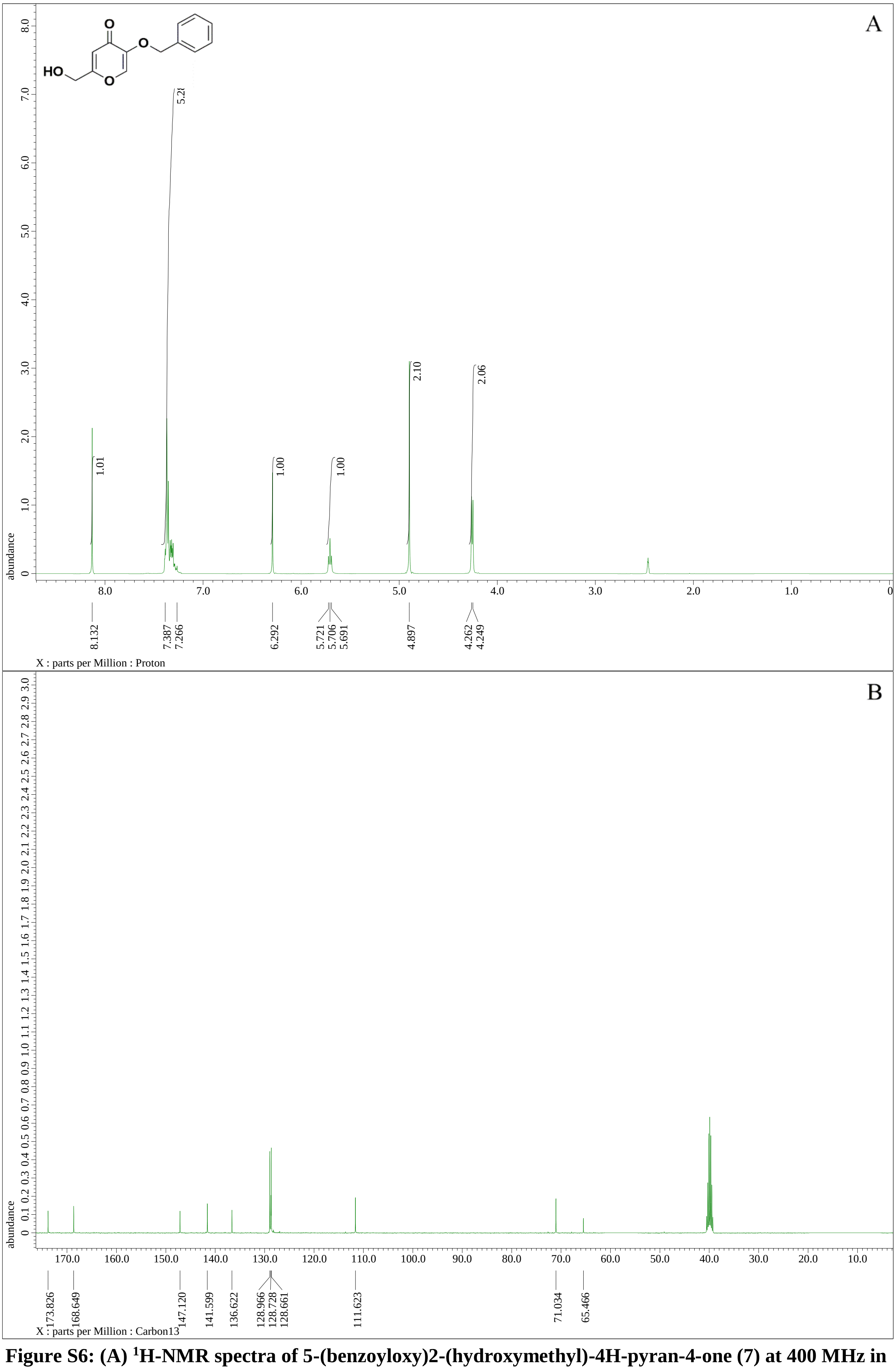

Figure S6: (A) ^{1}H-NMR spectra of 5-(benzoyloxy)2-(hydroxymethyl)-4H-pyran-4-one (7) at 400 MHz in DMSO-d_6 and (B) ^{13}C-NMR spectra of 5-(benzoyloxy)2-(hydroxymethyl)-4H-pyran-4-one (7) at 100 MHz in DMSO-d_6

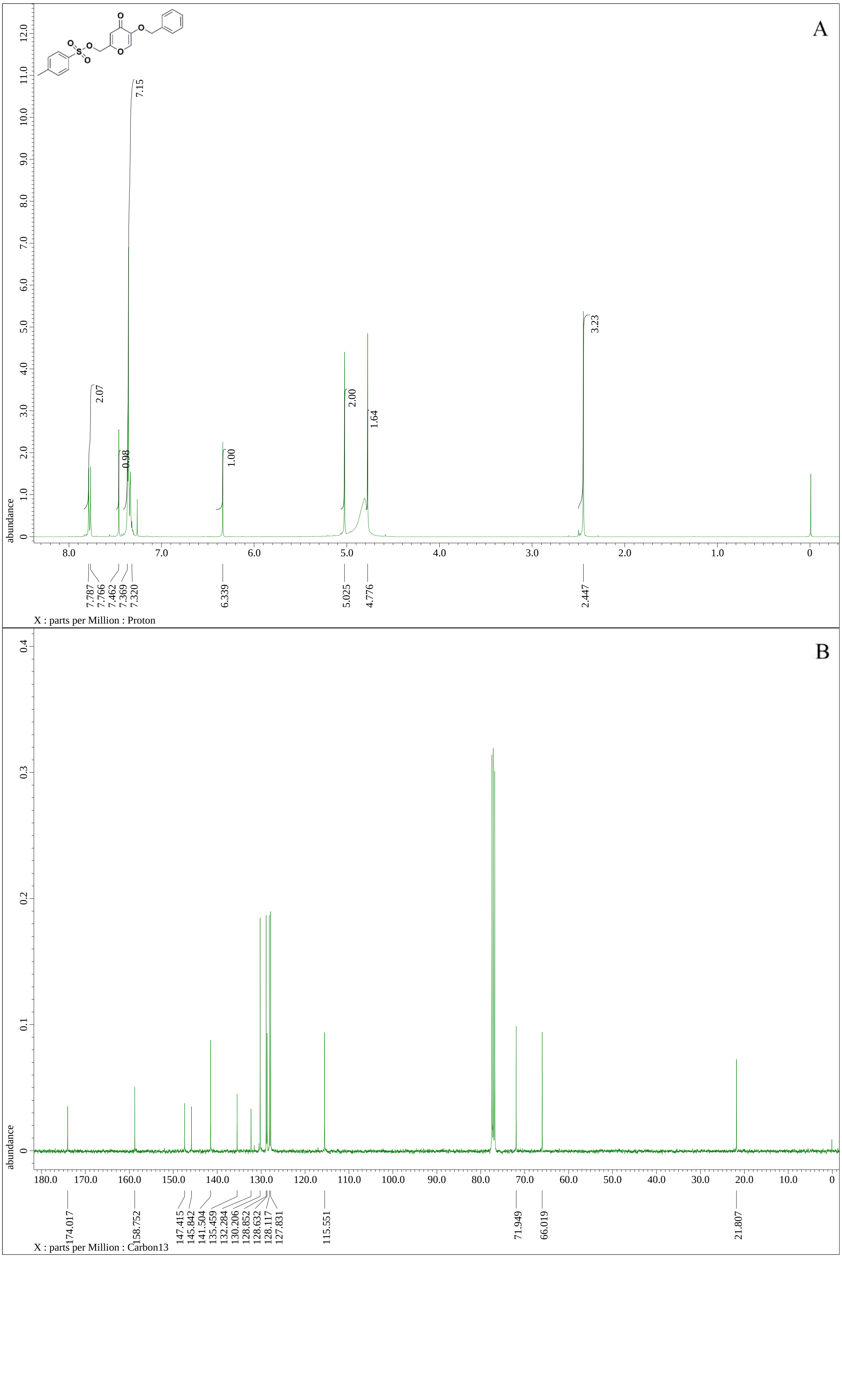

A
B
abundance
X : parts per Million : Proton
X : parts per Million : Carbon13
7.787
7.766
7.462
7.369
7.320
6.339
5.025
4.776
2.447
2.07
0.98
7.15
1.00
2.00
1.64
3.23
174.017
158.752
147.415
145.842
141.504
135.459
132.284
130.206
128.852
128.632
128.117
127.831
115.551
71.949
66.019
21.807

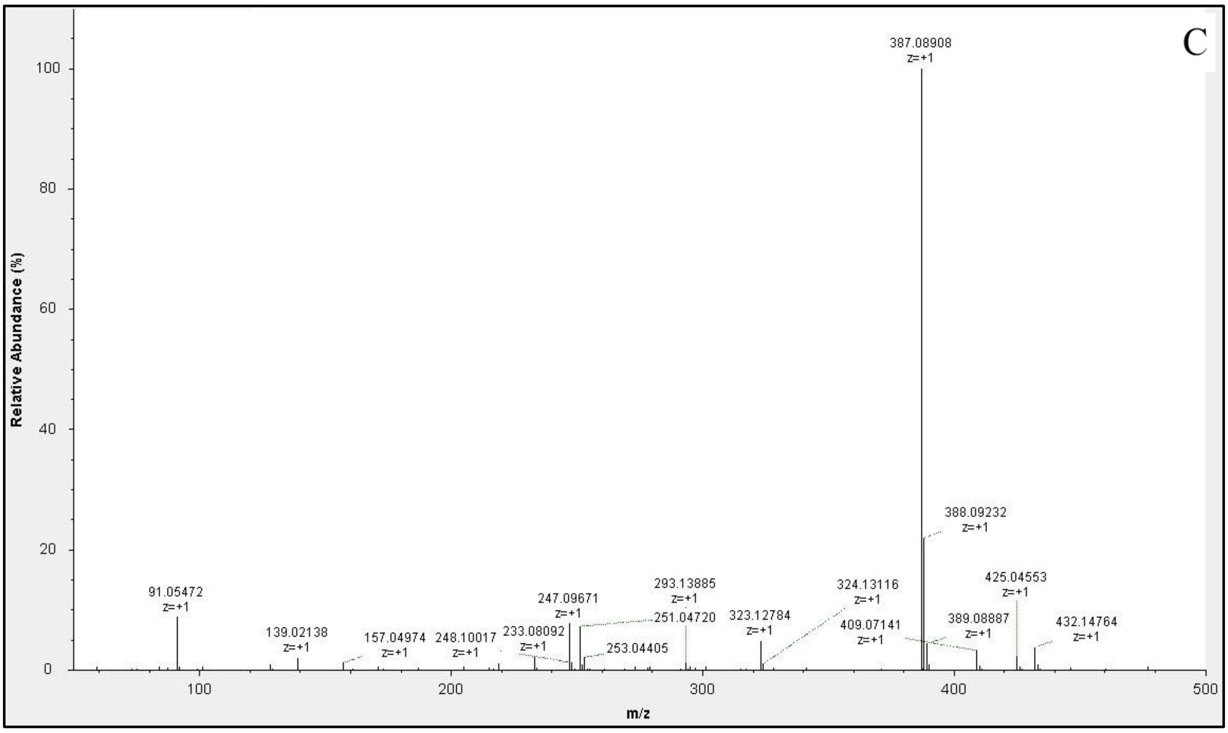

Figure S7: (A) ^{1}H-NMR spectra of [5-(benzyloxy)-4-oxo-4H-pyran-2-yl]methyl 4-methylbenzene-1-sulfonate (8) at 400 MHz in $CDCl_3$, (B)^{13}C-NMR spectra of [5-(benzyloxy)-4-oxo-4H-pyran-2-yl]methyl 4-methylbenzene-1-sulfonate (8) at 100 MHz in $CDCl_3$ and (C) HRMS of [5-(benzyloxy)-4-oxo-4H-pyran-2-yl]methyl 4-methylbenzene-1-sulfonate (8).

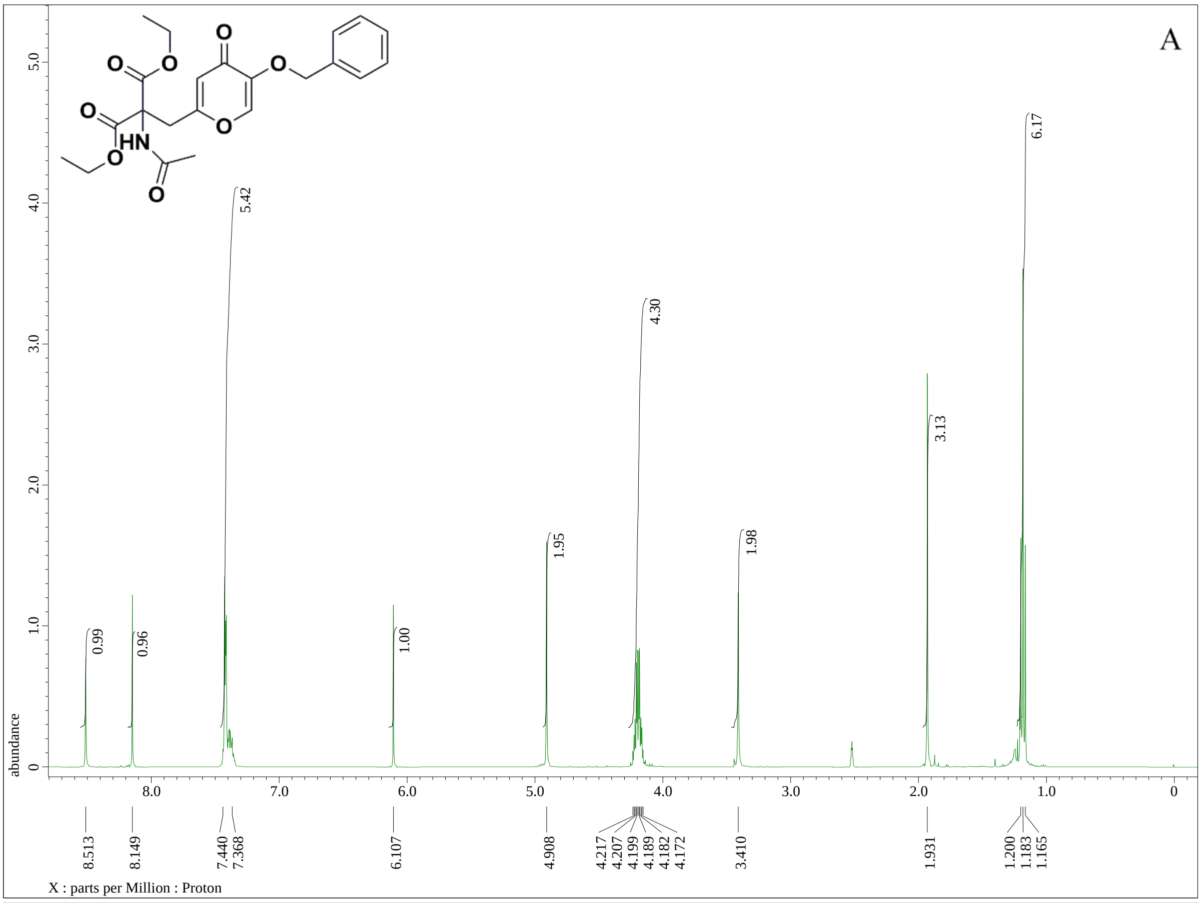
A
abundance
X : parts per Million : Proton
0.99
0.96
5.42
1.00
1.95
4.30
1.98
3.13
6.17
8.513
8.149
7.440
7.368
6.107
4.908
4.217
4.207
4.199
4.189
4.182
4.172
3.410
1.931
1.200
1.183
1.165

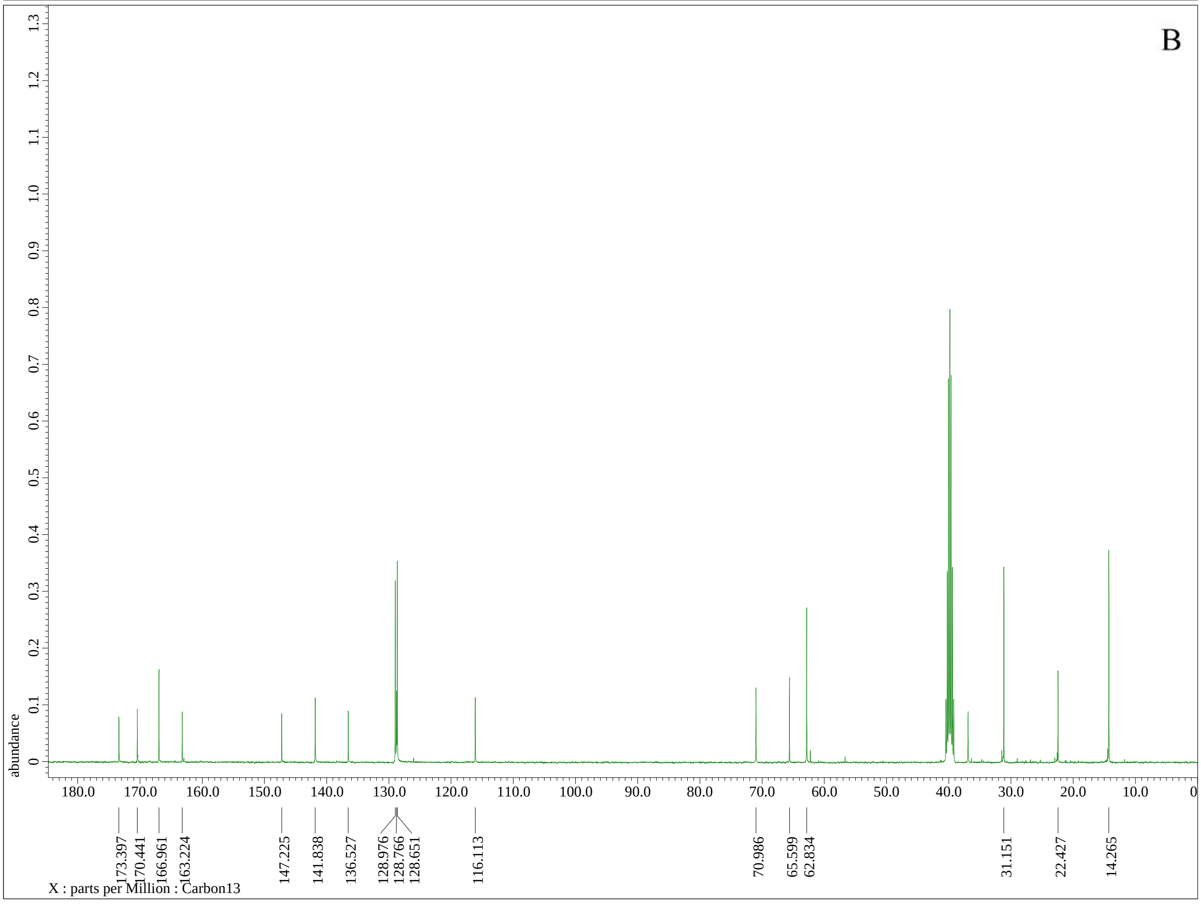
B
abundance
X : parts per Million : Carbon13
173.397
170.441
166.961
163.224
147.225
141.838
136.527
128.976
128.766
128.651
116.113
70.986
65.599
62.834
31.151
22.427
14.265

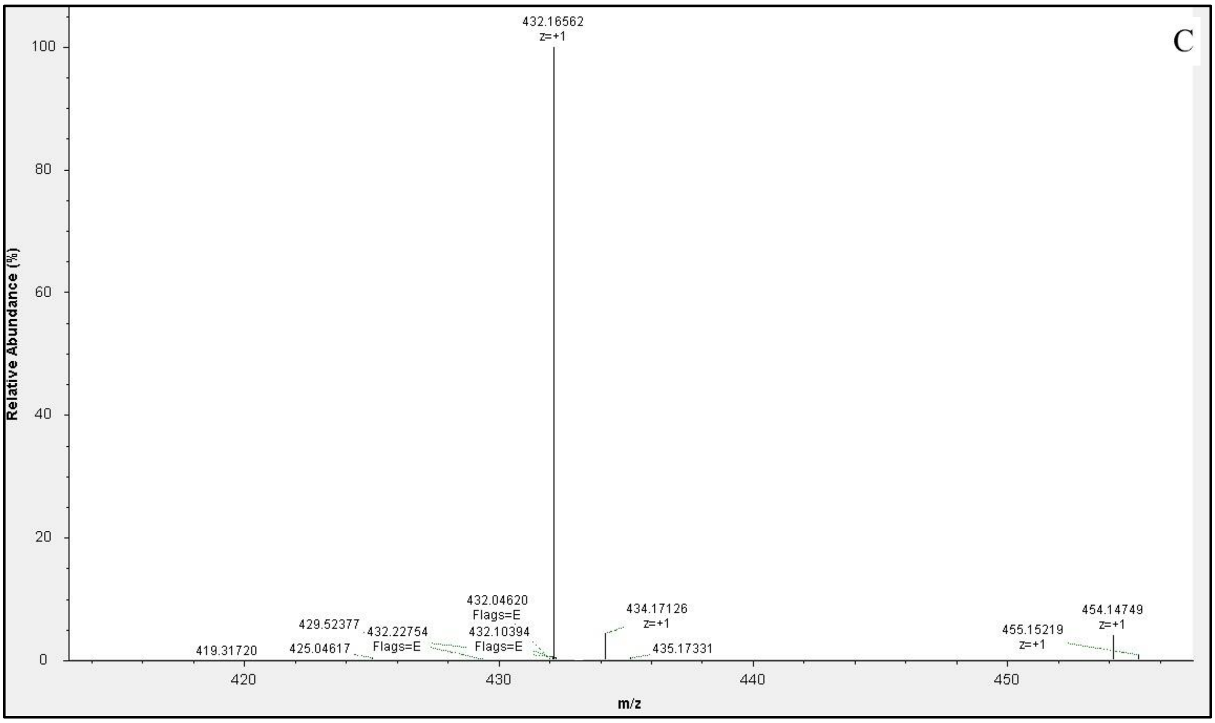

Figure S8: (A) ^{1}H-NMR spectra of 1,3-diethyl 2-{[5-(benzyloxy)-4-oxo-4H-pyran-2-yl]methyl}-2-acetamidopropanedioate (9) at 400 MHz in DMSO-d_6 and (B)^{13}C-NMR spectra of 1,3-diethyl 2-{[5-(benzyloxy)-4-oxo-4H-pyran-2-yl]methyl}-2-acetamidopropanedioate (9) at 100 MHz in DMSO-d_6 and (C) HRMS of 1,3-diethyl 2-{[5-(benzyloxy)-4-oxo-4H-pyran-2-yl]methyl}-2-acetamidopropanedioate (9)

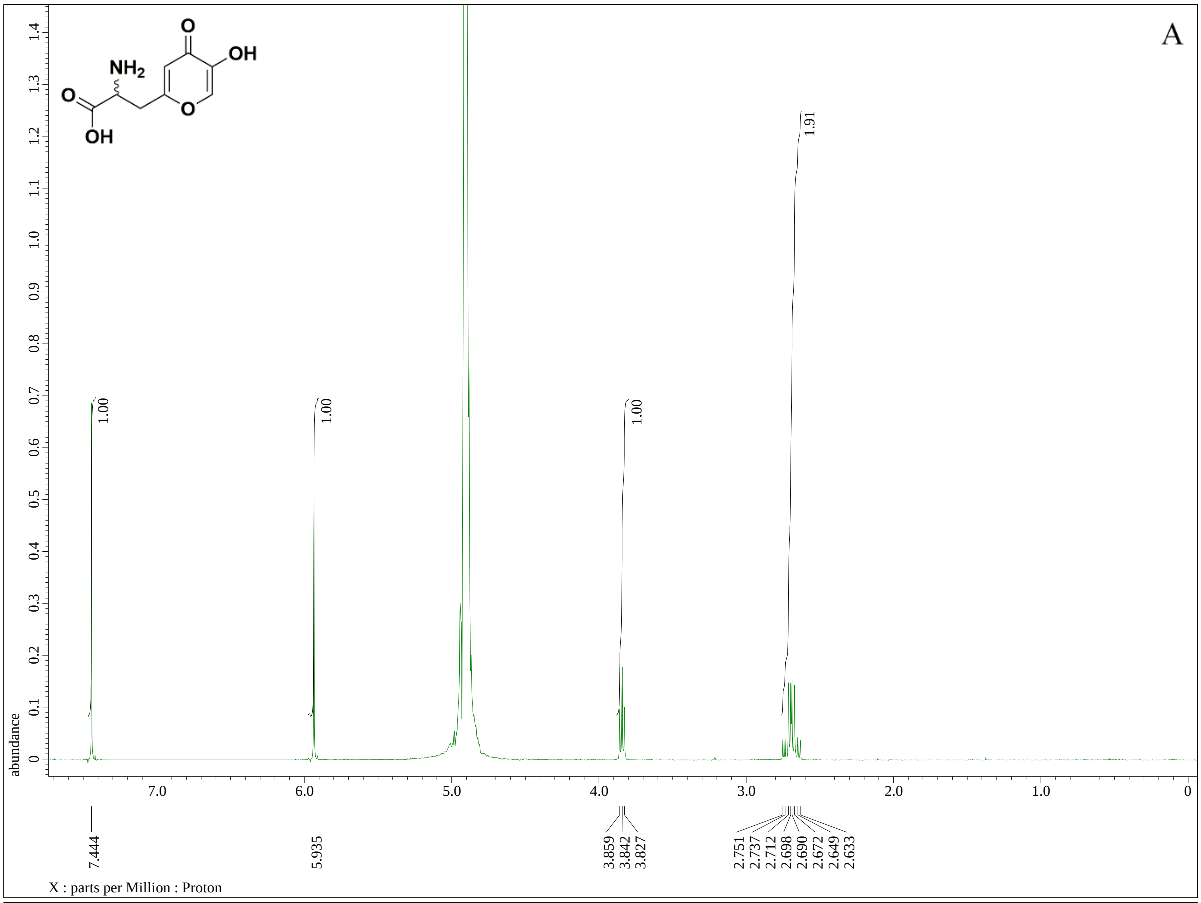
A
NH2
O
OH
OH
O
O
1.00
1.00
1.00
1.91
abundance
7.0
6.0
5.0
4.0
3.0
2.0
1.0
0
7.444
5.935
3.859
3.842
3.827
2.751
2.737
2.712
2.698
2.690
2.672
2.649
2.633
X : parts per Million : Proton

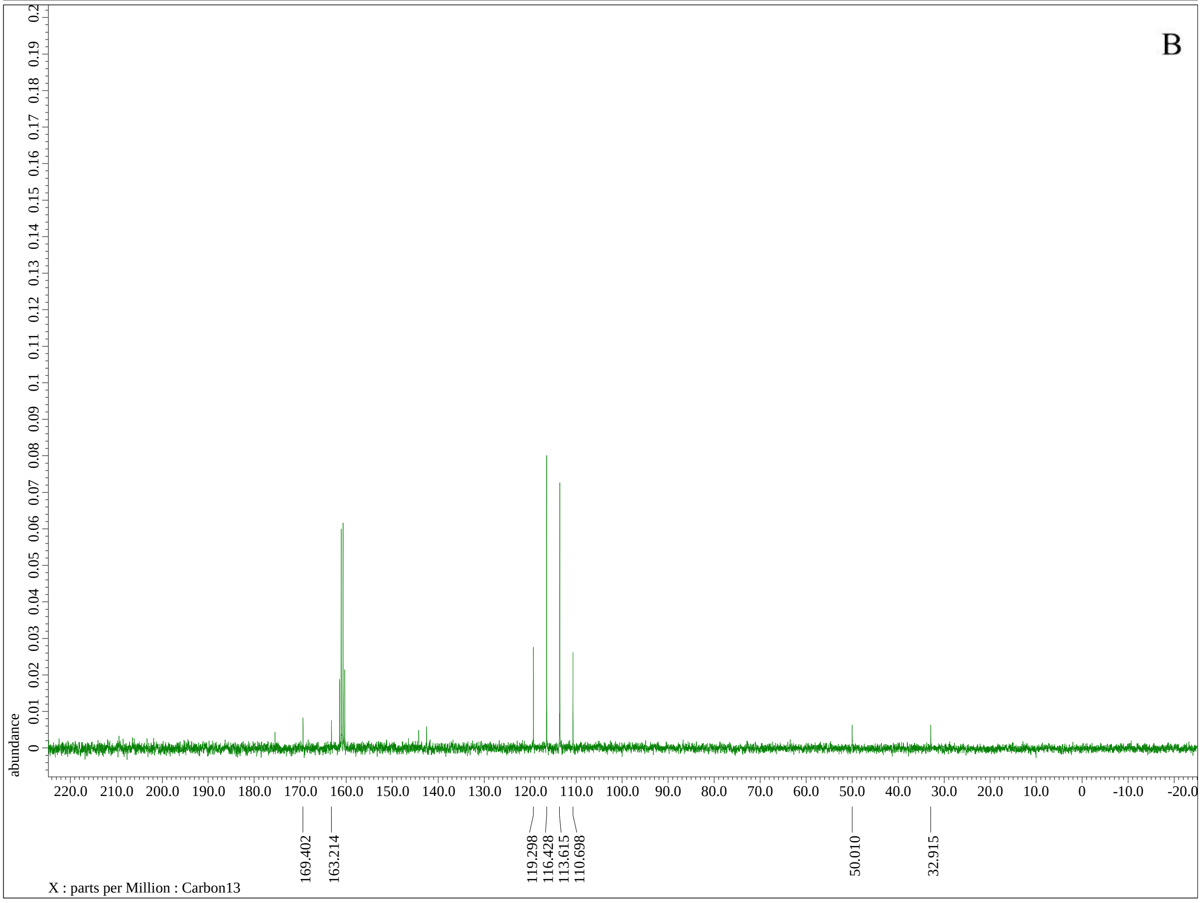
B
abundance
169.402
163.214
119.298
116.428
113.615
110.698
50.010
32.915
X : parts per Million : Carbon13

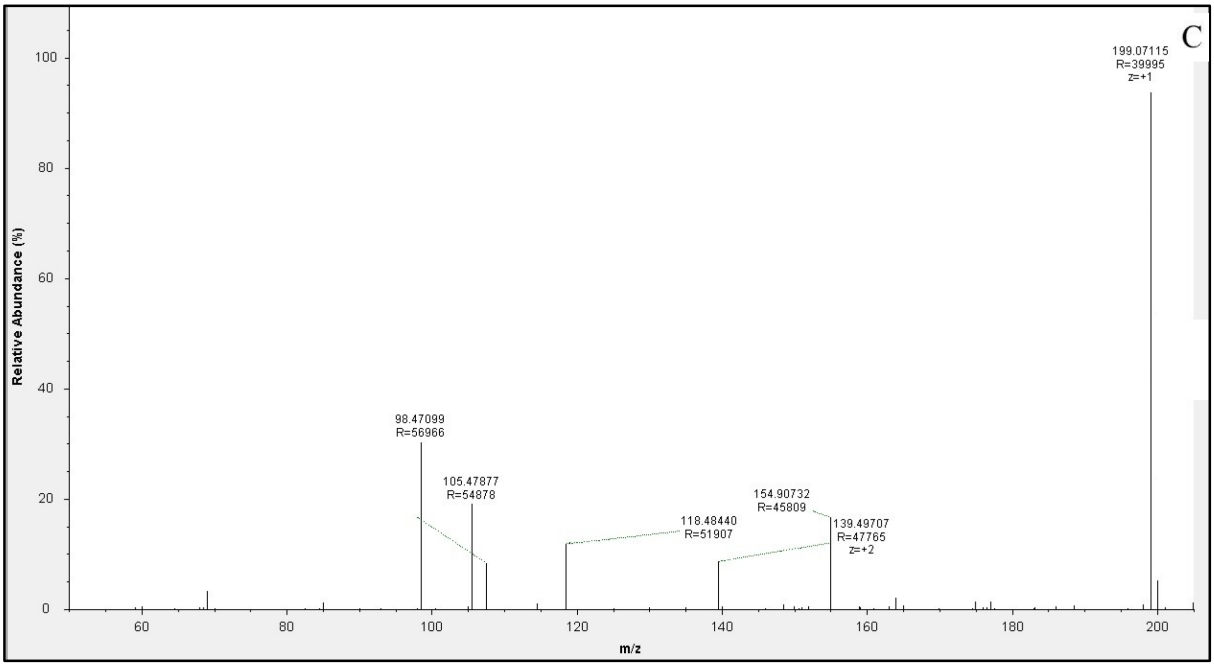

Figure S9: ^{1}H-NMR spectra r*ac*-2-amino-3-(5-hydroxy-4-oxo-4H-pyran-2-yl)propanoic acid (10) at 400 MHz in D_2O/CF_3CO_2D and (B)^{13}C-NMR spectra of *rac*-2-amino-3-(5-hydroxy-4-oxo-4H-pyran-2-yl)propanoic acid (10) at 100 MHz in D_2O/CF_3CO_2D and (C) HRMS of *rac*-2-amino-3-(5-hydroxy-4-oxo-4H-pyran-2-yl)propanoic acid (10).

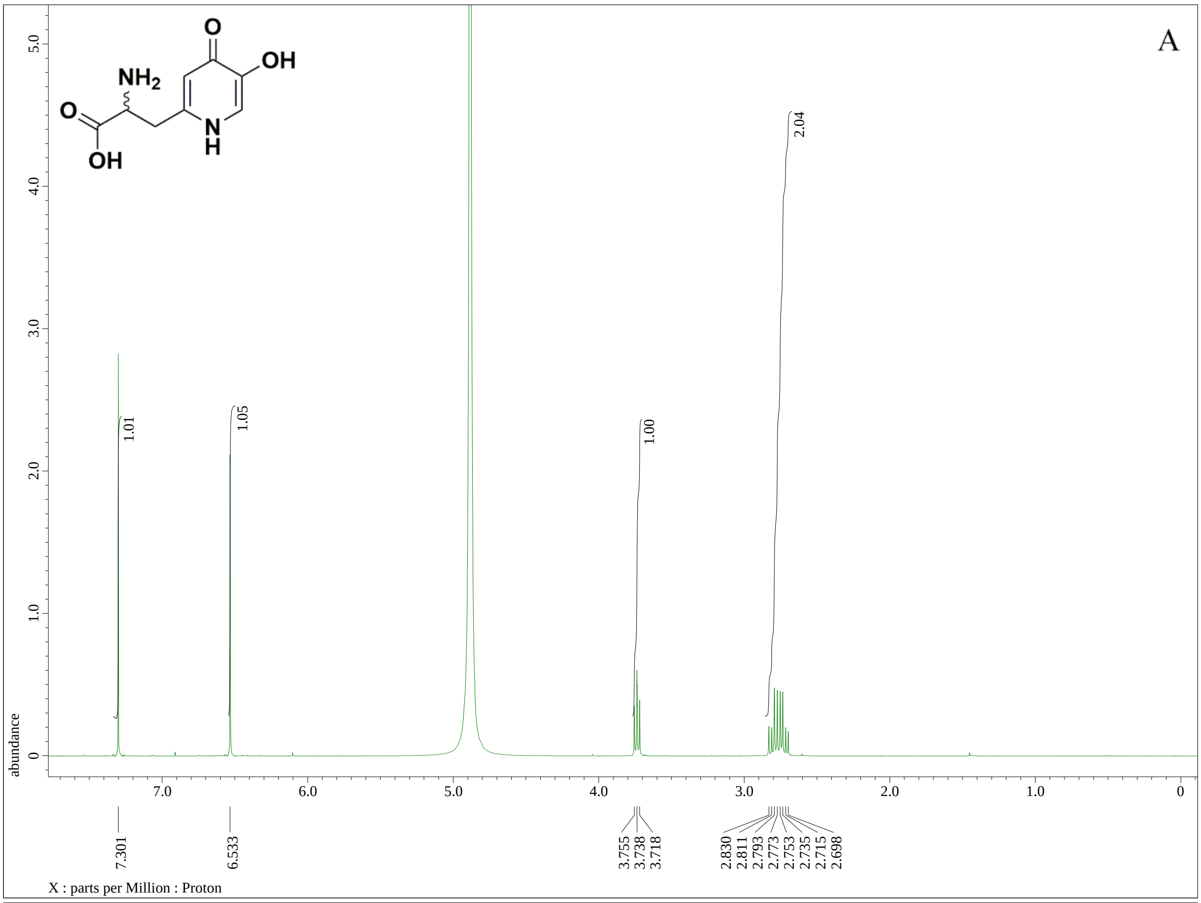
A
NH2
O
OH
N
H
abundance
1.01
1.05
1.00
2.04
7.301
6.533
3.755
3.738
3.718
2.830
2.811
2.793
2.773
2.753
2.735
2.715
2.698
X : parts per Million : Proton

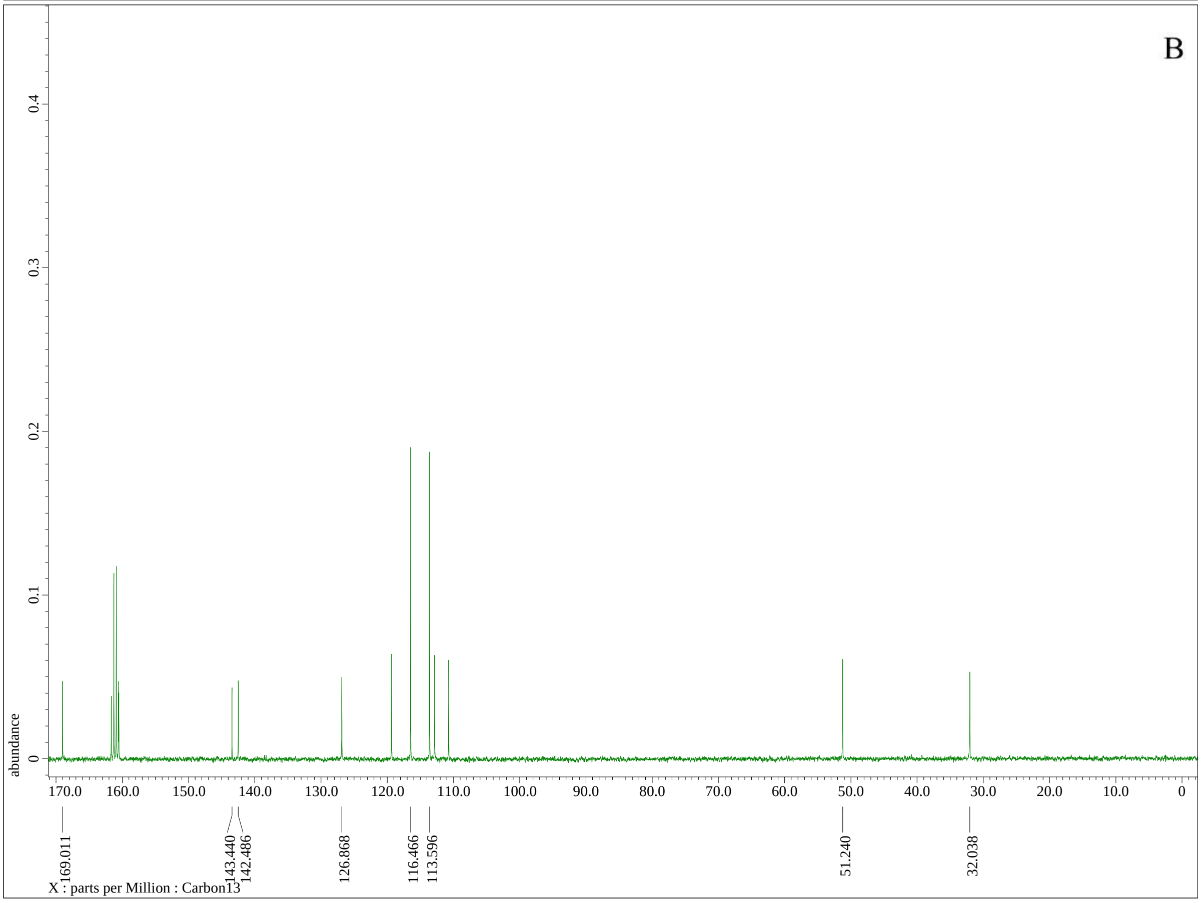
B
abundance
169.011
143.440
142.486
126.868
116.466
113.596
51.240
32.038
X : parts per Million : Carbon13

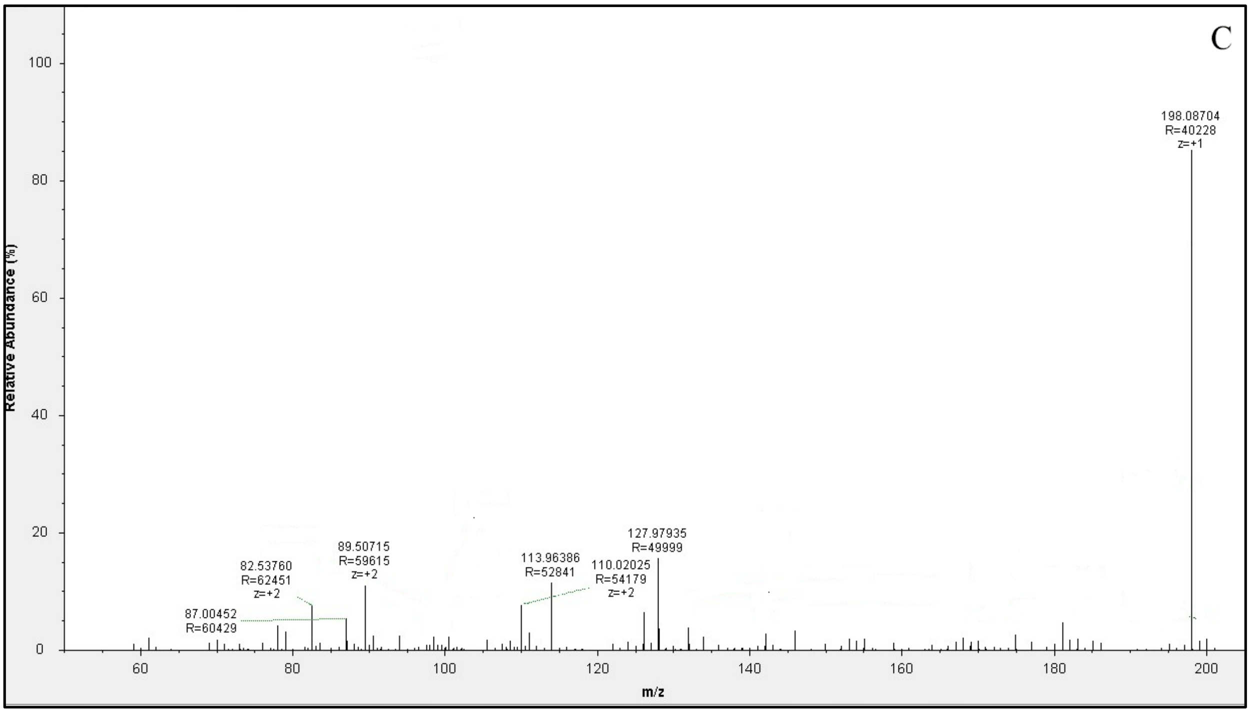

Figure S10: ^{1}H-NMR spectra *rac*-2-amino-3-(5-hydroxy-4-oxo-1,4-dihydropyridin-2-yl)propanoic acid (11) at 400 MHz in D_2O/CF_3CO_2D and (B)^{13}C-NMR spectra of *rac*-2-amino-3-(5-hydroxy-4-oxo-1,4-dihydropyridin-2-yl)propanoic acid (11) at 100 MHz in D_2O/CF_3CO_2D and (C) HRMS of *rac*-2-amino-3-(5-hydroxy-4-oxo-1,4-dihydropyridin-2-yl)propanoic acid (11)

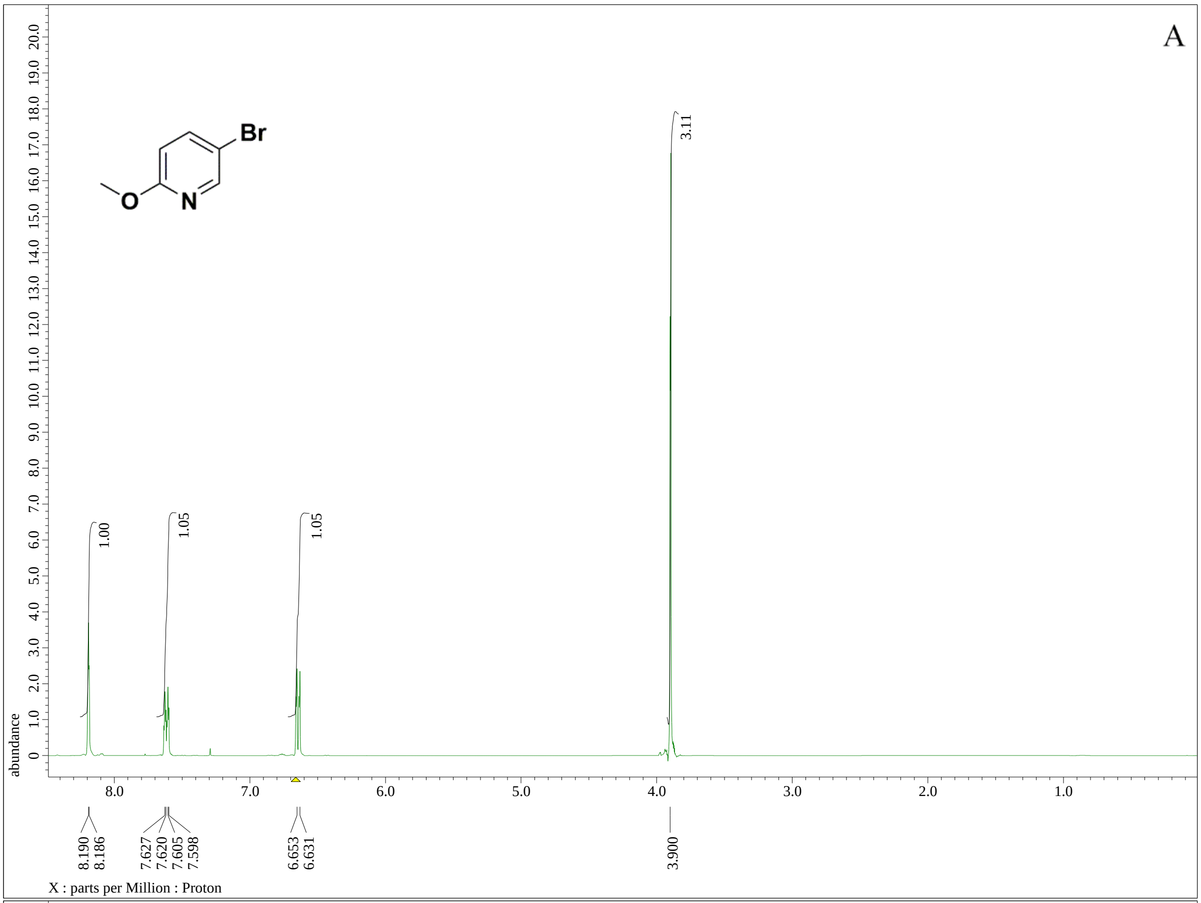

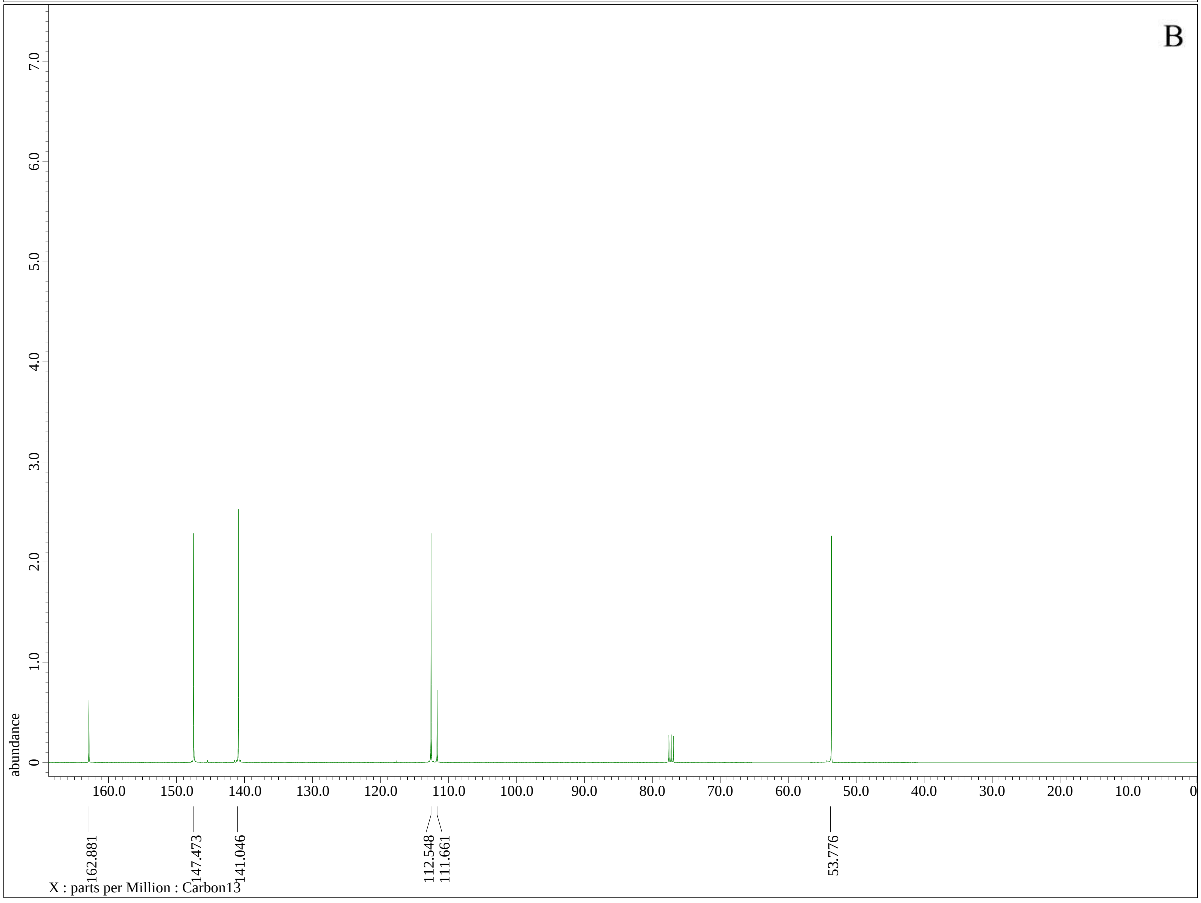

Figure S11: (A) ^{1}H-NMR spectra of 5-bromo-2-methoxypyridine (12) at 400 MHz in $CDCl_3$, (B)^{13}C-NMR spectra of 5-bromo-2-methoxypyridine (12) at 100 MHz in $CDCl_3$.

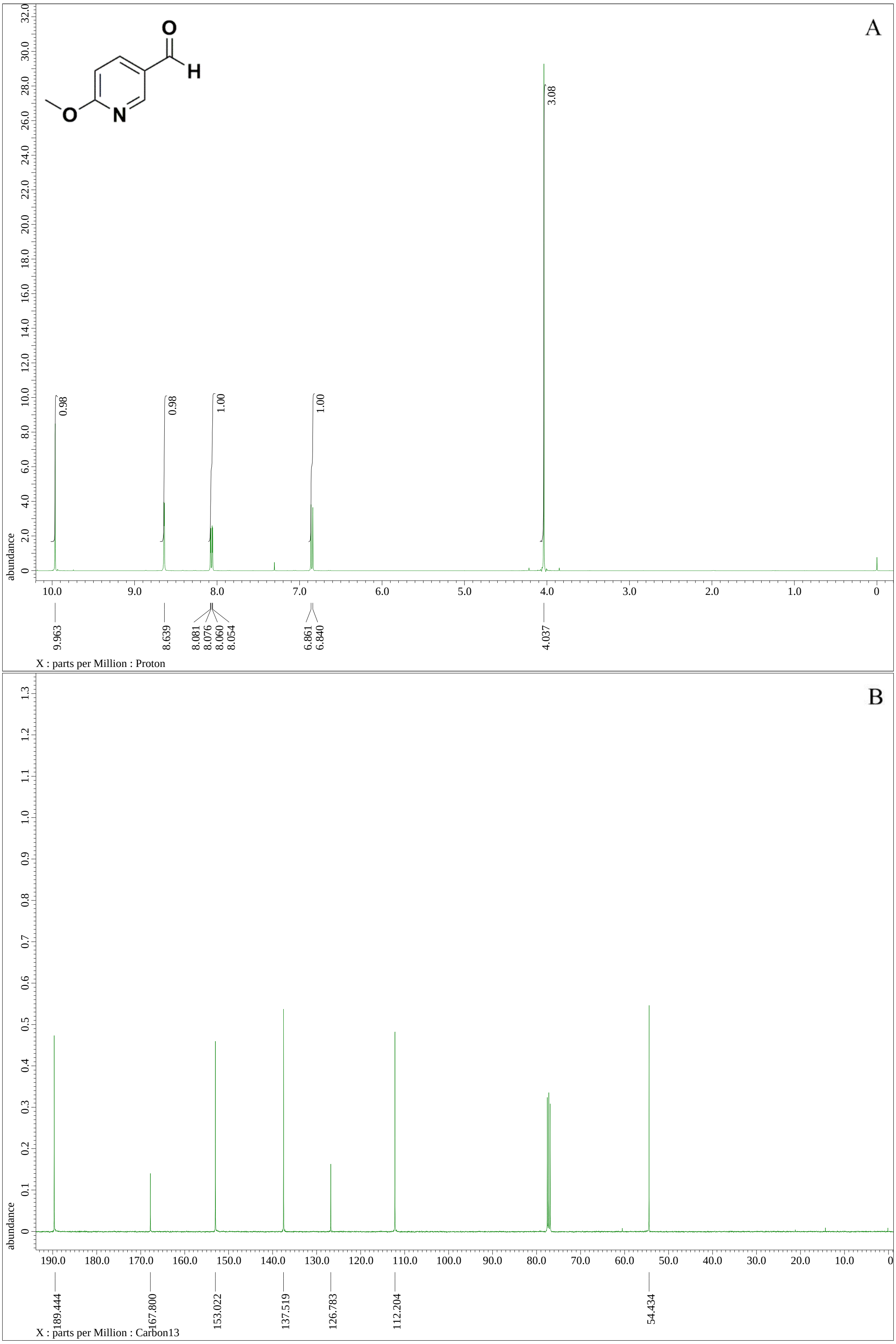

Figure S12: (A) ^{1}H-NMR spectra of 6-methoxypyridine-3-carbaldehyde (13) at 400 MHz in $CDCl_3$, (B)^{13}C-NMR spectra of 6-methoxypyridine-3-carbaldehyde (13) at 100 MHz in $CDCl_3$.

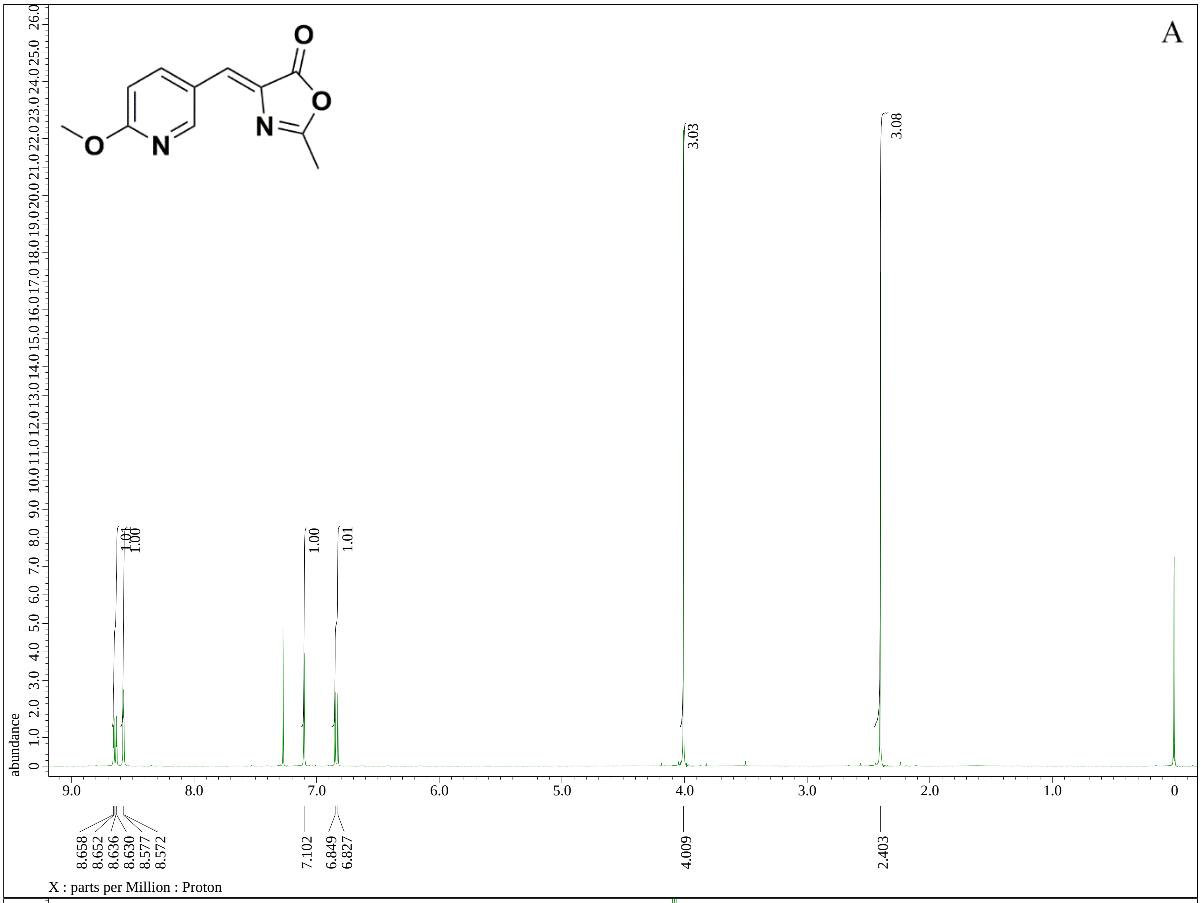
A
abundance
1.01
1.00
1.00
1.01
3.03
3.08
8.658
8.652
8.636
8.630
8.577
8.572
7.102
6.849
6.827
4.009
2.403
X : parts per Million : Proton

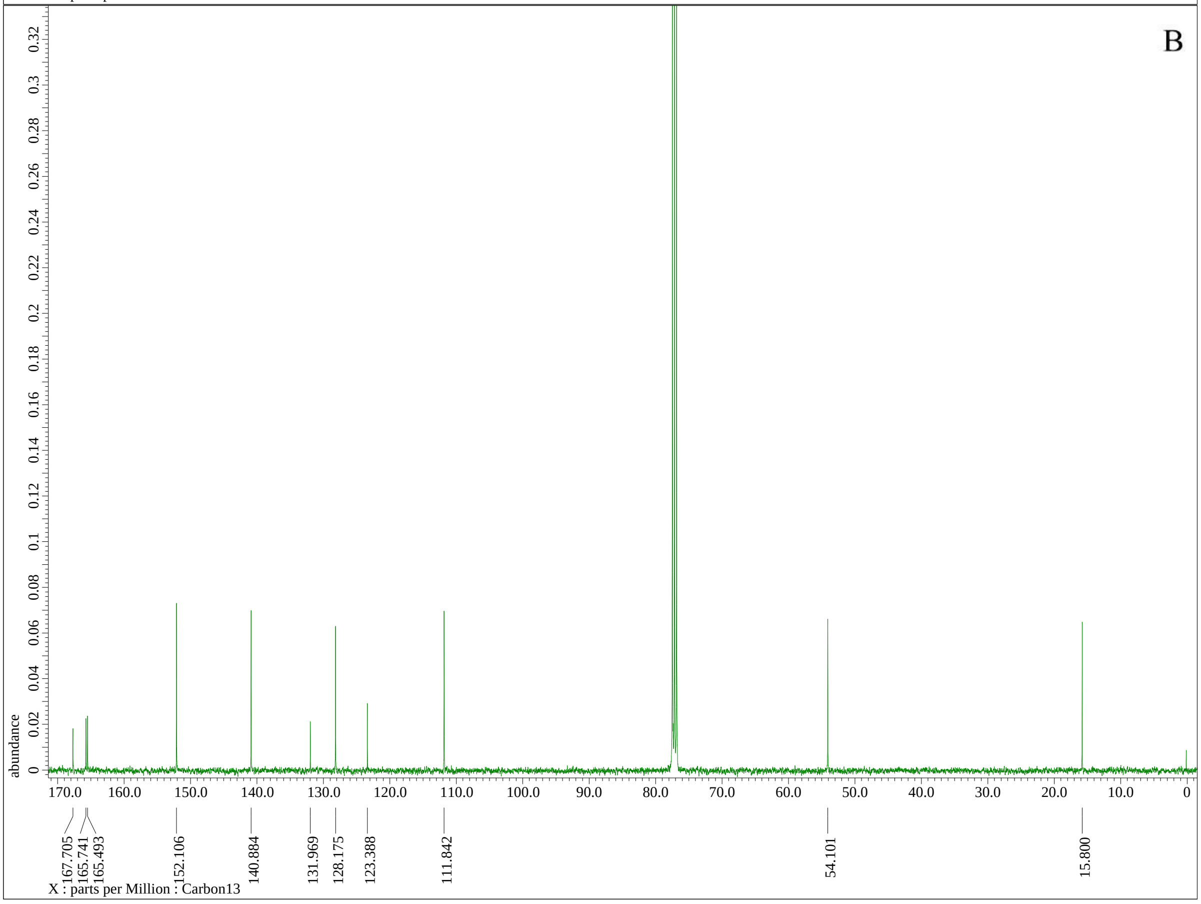
B
abundance
167.705
165.741
165.493
152.106
140.884
131.969
128.175
123.388
111.842
54.101
15.800
X : parts per Million : Carbon13

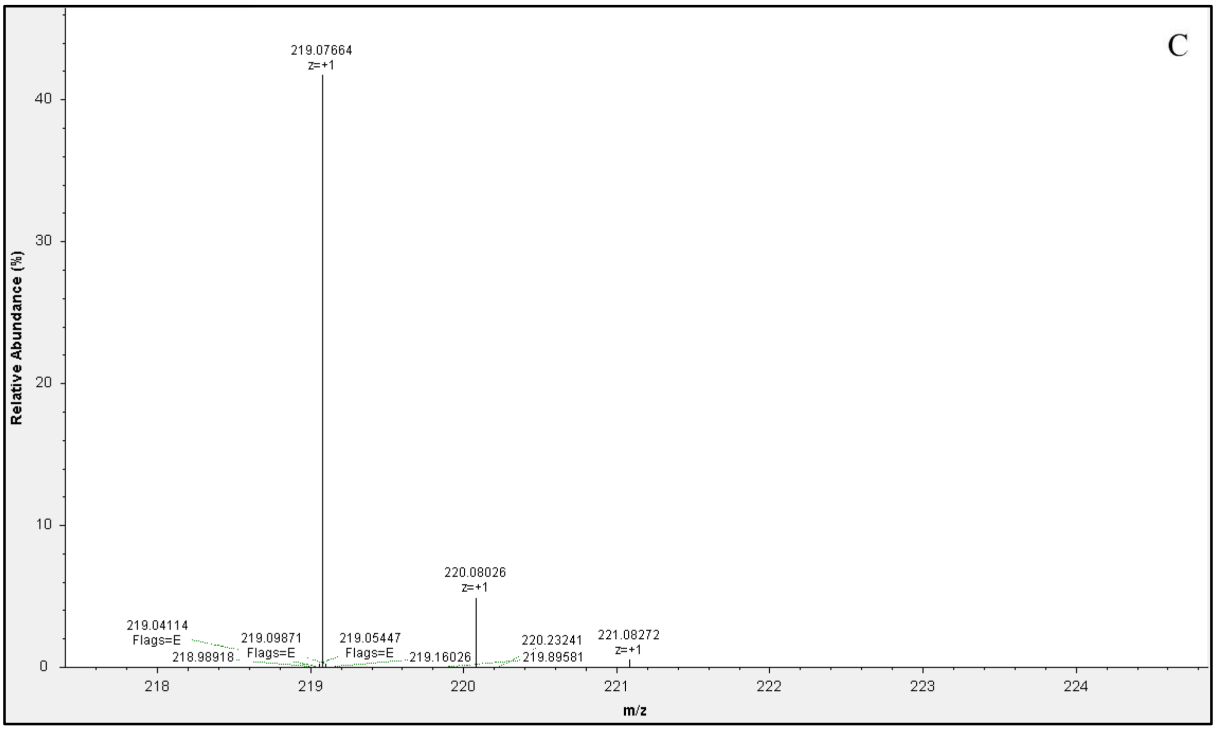

Figure S13: ^{1}H-NMR spectra (4Z)-4-[(6-methoxypyridin-3-yl)methylidene]-2-methyl-4,5-dihydro-1,3-oxazol-5-one (14) at 400 MHz in $CDCl_3$ and (B)^{13}C-NMR spectra of (4Z)-4-[(6-methoxypyridin-3-yl)methylidene]-2-methyl-4,5-dihydro-1,3-oxazol-5-one (14) at 100 MHz in $CDCl_3$ and (C) HRMS of (4Z)-4-[(6-methoxypyridin-3-yl)methylidene]-2-methyl-4,5-dihydro-1,3-oxazol-5-one (14).

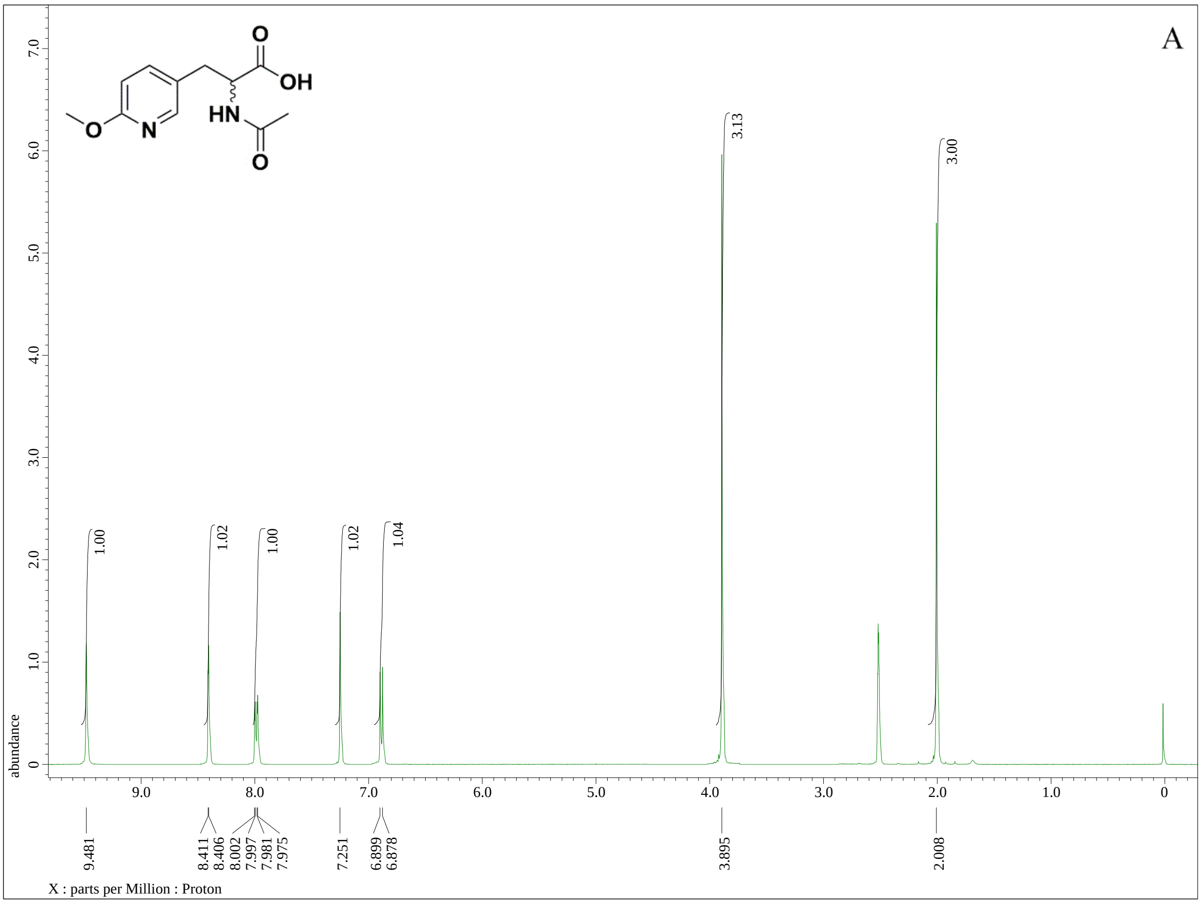
A
O
OH
HN
O
N
O
abundance
1.00
1.02
1.00
1.02
1.04
3.13
3.00
9.481
8.411
8.406
8.002
7.997
7.981
7.975
7.251
6.899
6.878
3.895
2.008
X : parts per Million : Proton

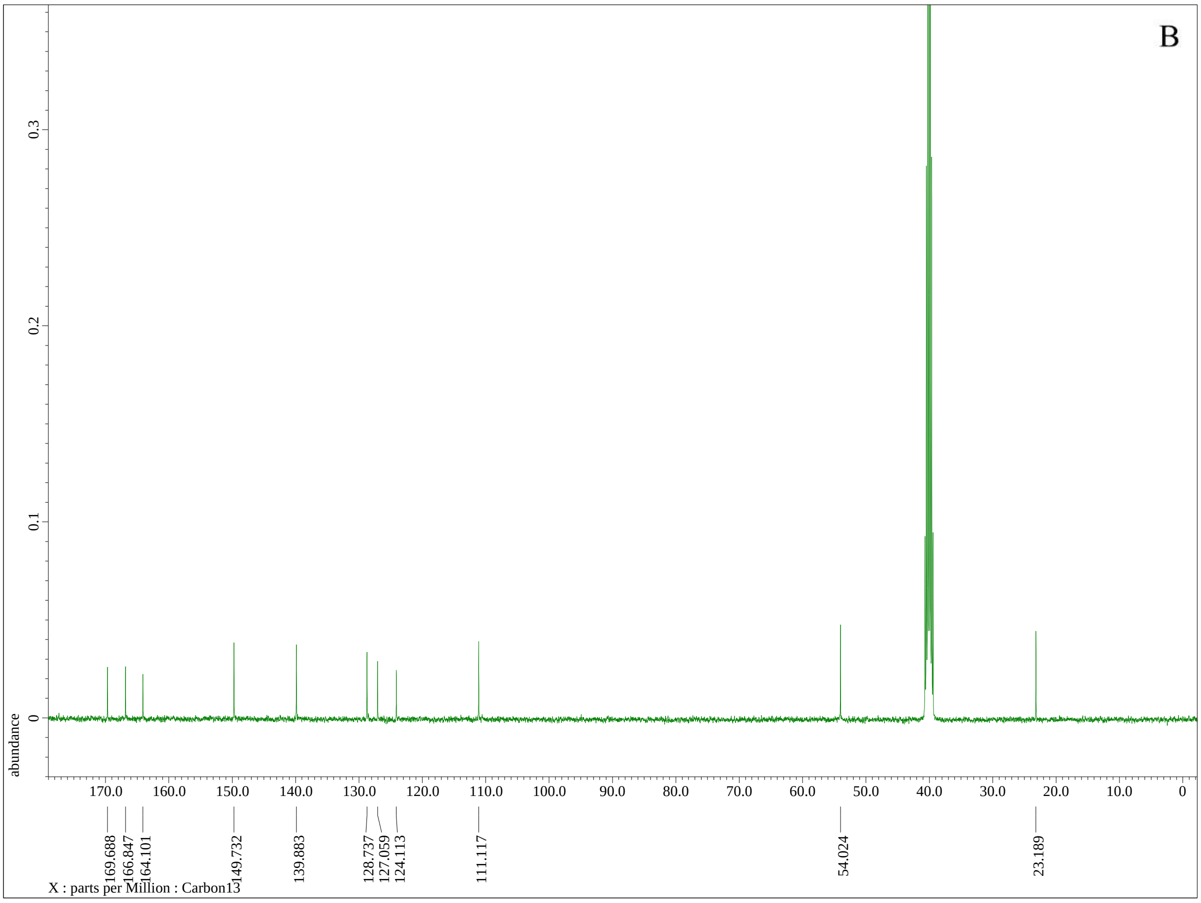
B
abundance
169.688
166.847
164.101
149.732
139.883
128.737
127.059
124.113
111.117
54.024
23.189
X : parts per Million : Carbon13

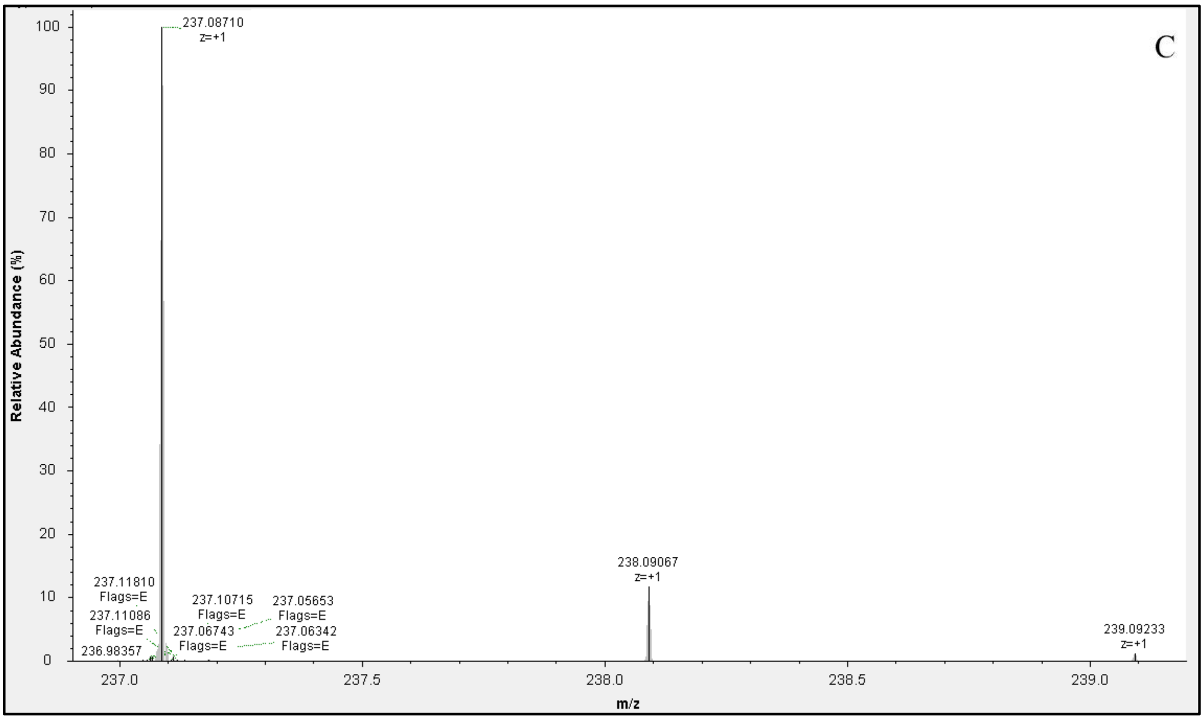

Figure S14: ^{1}H-NMR spectra of (2Z)-2-acetamido-3-(6-methoxypyridin-3-yl)prop-2-enoic acid (15) at 400 MHz in $CDCl_3$ and (B)^{13}C-NMR spectra of (2Z)-2-acetamido-3-(6-methoxypyridin-3-yl)prop-2-enoic acid (15) at 100 MHz in $CDCl_3$ and (C) HRMS of (2Z)-2-acetamido-3-(6-methoxypyridin-3-yl)prop-2-enoic acid (15)

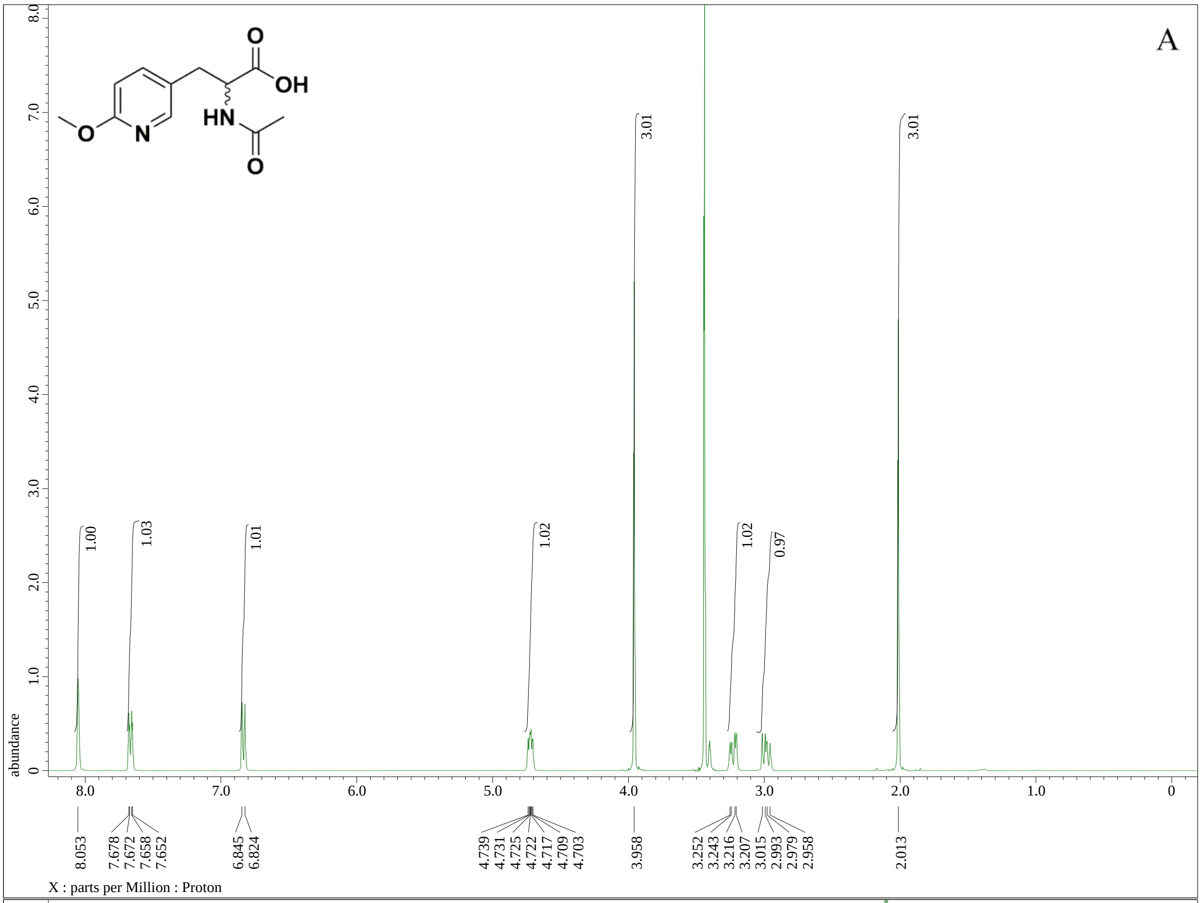
A
O
OH
HN
O
N
O
1.00
1.03
1.01
1.02
3.01
1.02
0.97
3.01
8.053
7.678
7.672
7.658
7.652
6.845
6.824
4.739
4.731
4.725
4.722
4.717
4.709
4.703
3.958
3.252
3.243
3.216
3.207
3.015
2.993
2.979
2.958
2.013
abundance
X : parts per Million : Proton

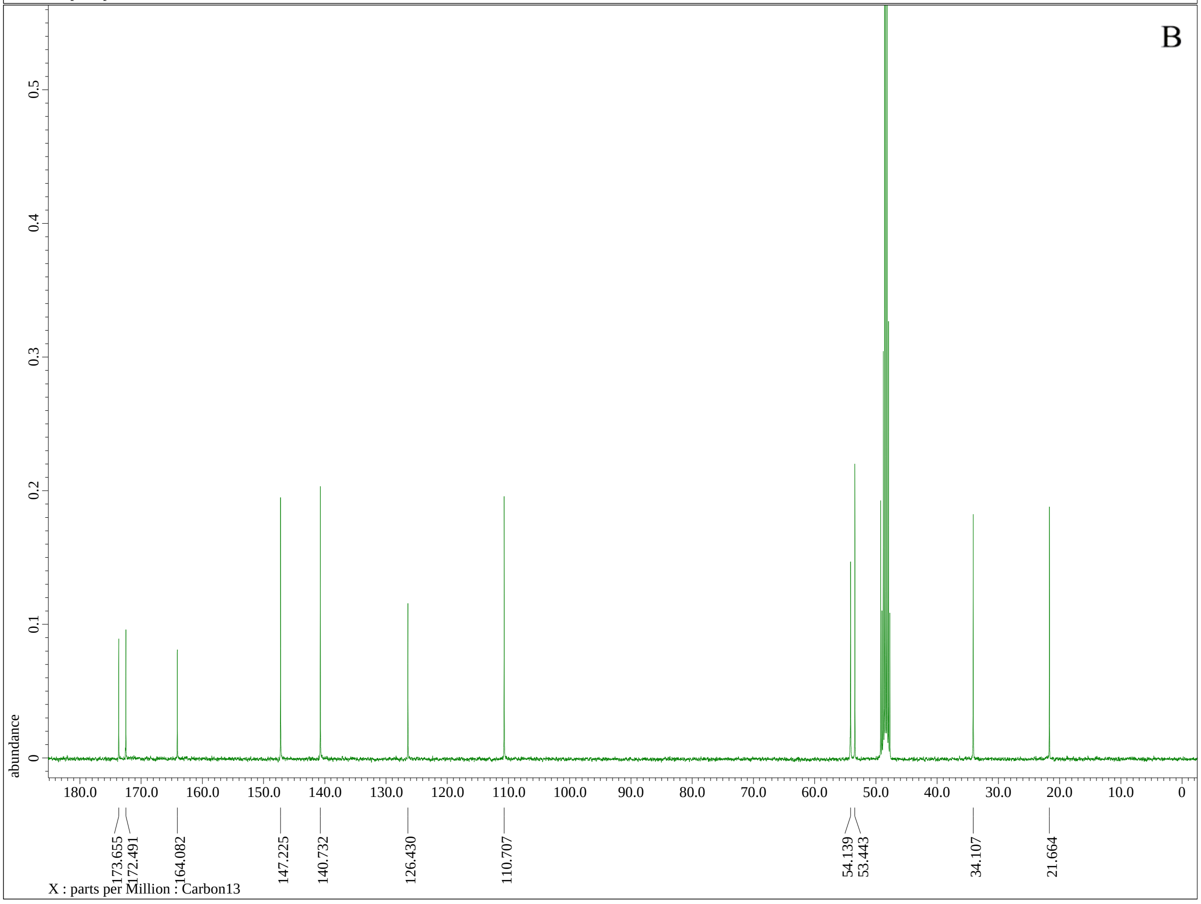
B
173.655
172.491
164.082
147.225
140.732
126.430
110.707
54.139
53.443
34.107
21.664
abundance
X : parts per Million : Carbon13

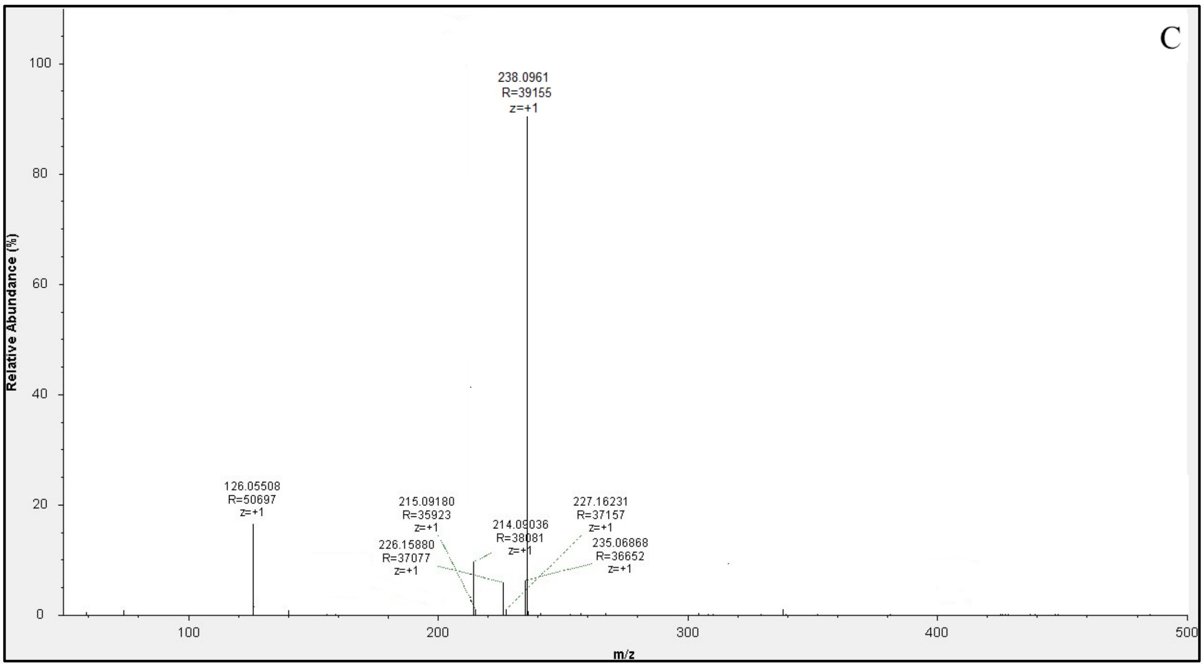

Figure S15: ^{1}H-NMR spectra of *rac*-2-acetamido-3-(6-methoxypyridin-3-yl)propanoic acid (16) at 400 MHz in MeOH-d_4 and (B)^{13}C-NMR spectra of *rac*-2-acetamido-3-(6-methoxypyridin-3-yl)propanoic acid (16) at 100 MHz in MeOH-d_4 and (C) HRMS of *rac*-2-acetamido-3-(6-methoxypyridin-3-yl)propanoic acid (16)

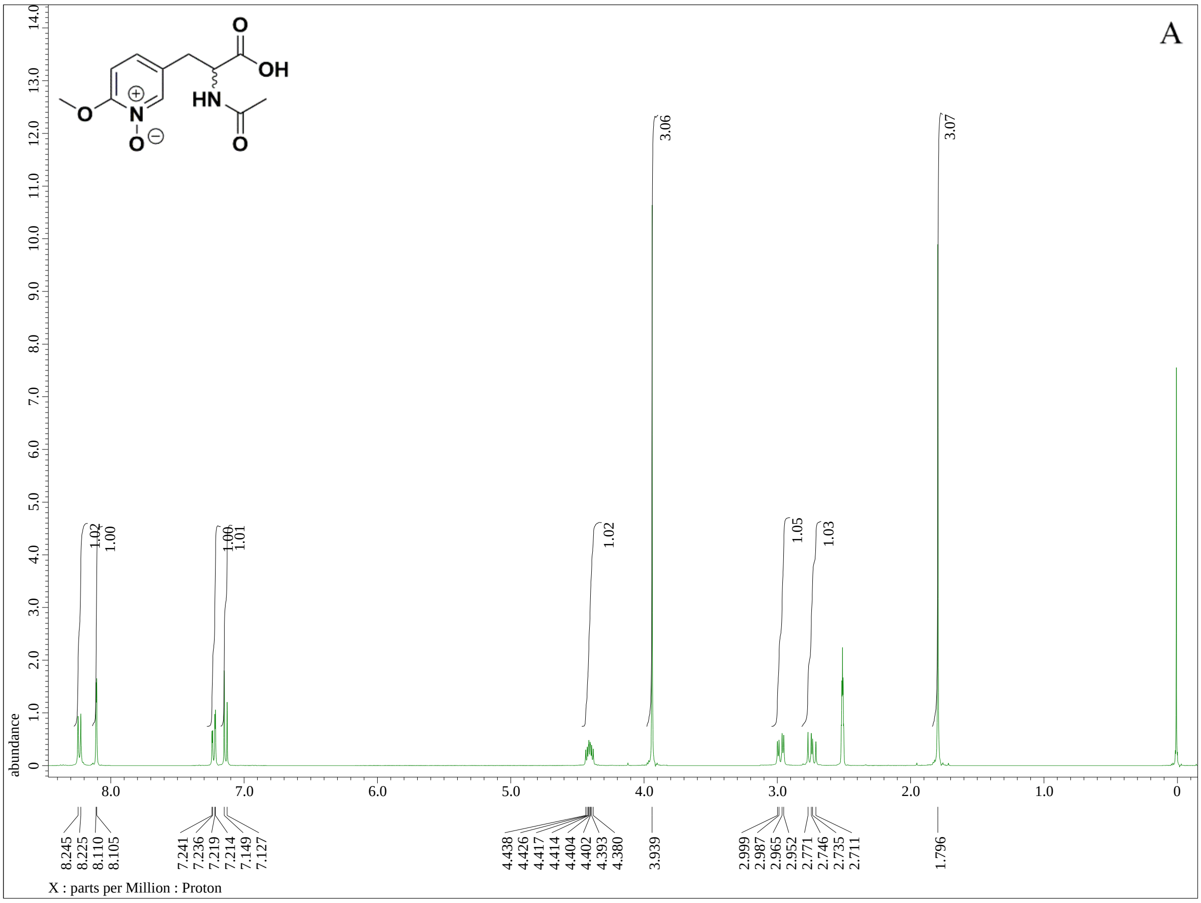
A
1.02
1.00
1.00
1.01
1.02
3.06
1.05
1.03
3.07
8.245
8.225
8.110
8.105
7.241
7.236
7.219
7.214
7.149
7.127
4.438
4.426
4.417
4.414
4.404
4.402
4.393
4.380
3.939
2.999
2.987
2.965
2.952
2.771
2.746
2.735
2.711
1.796
X : parts per Million : Proton
abundance

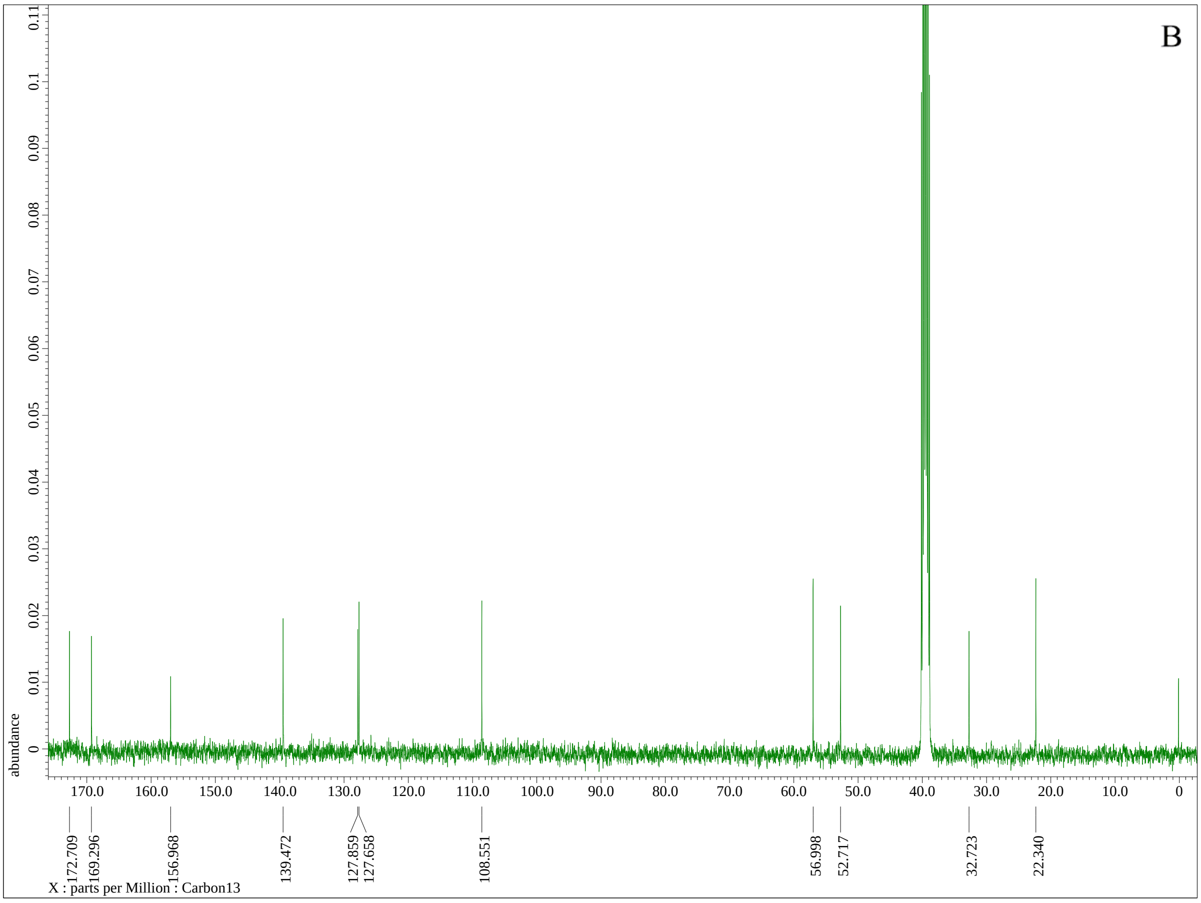
B
172.709
169.296
156.968
139.472
127.859
127.658
108.551
56.998
52.717
32.723
22.340
X : parts per Million : Carbon13
abundance

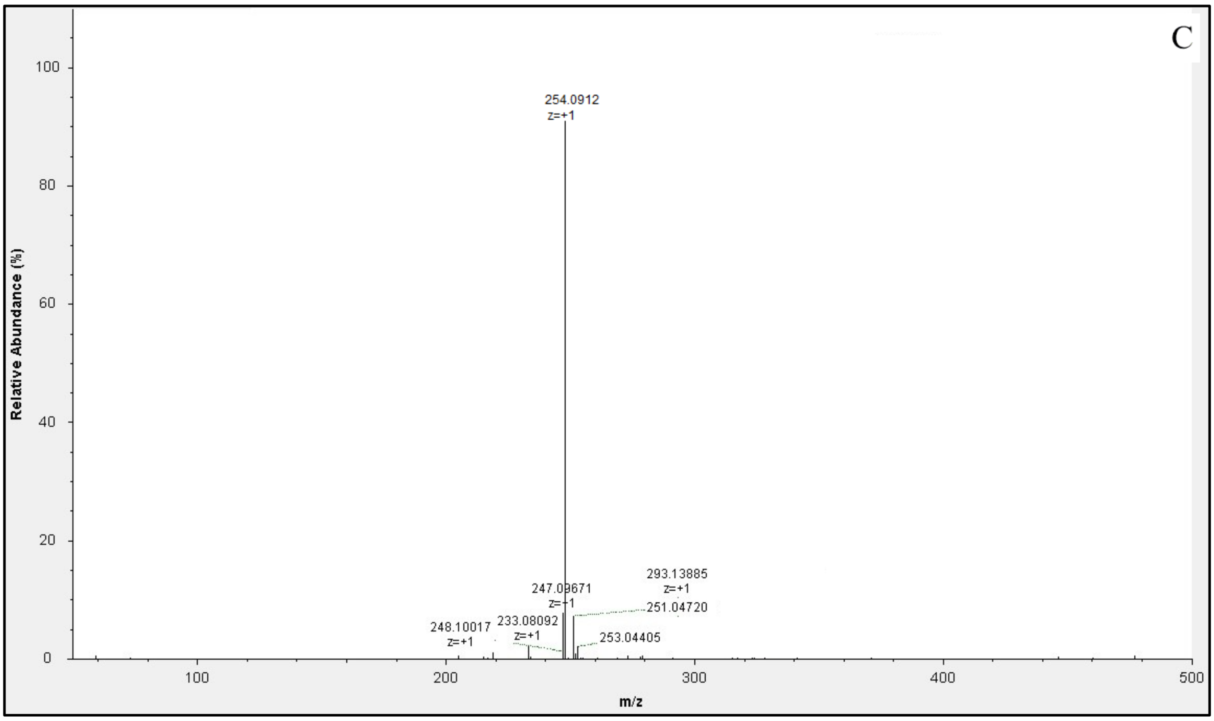

Figure S16: **^{1}H-NMR spectra of rac-5-(2-carboxy-2-acetamidoethyl)-2-methoxypyridin-1-ium-1-olate (17) at 400 MHz in DMSO-d_6 and (B)^{13}C-NMR spectra of rac-5-(2-carboxy-2-acetamidoethyl)-2-methoxypyridin-1-ium-1-olate (17) at 100 MHz in DMSO-d_6 and (C) HRMS of rac-5-(2-carboxy-2-acetamidoethyl)-2-methoxypyridin-1-ium-1-olate (17)**

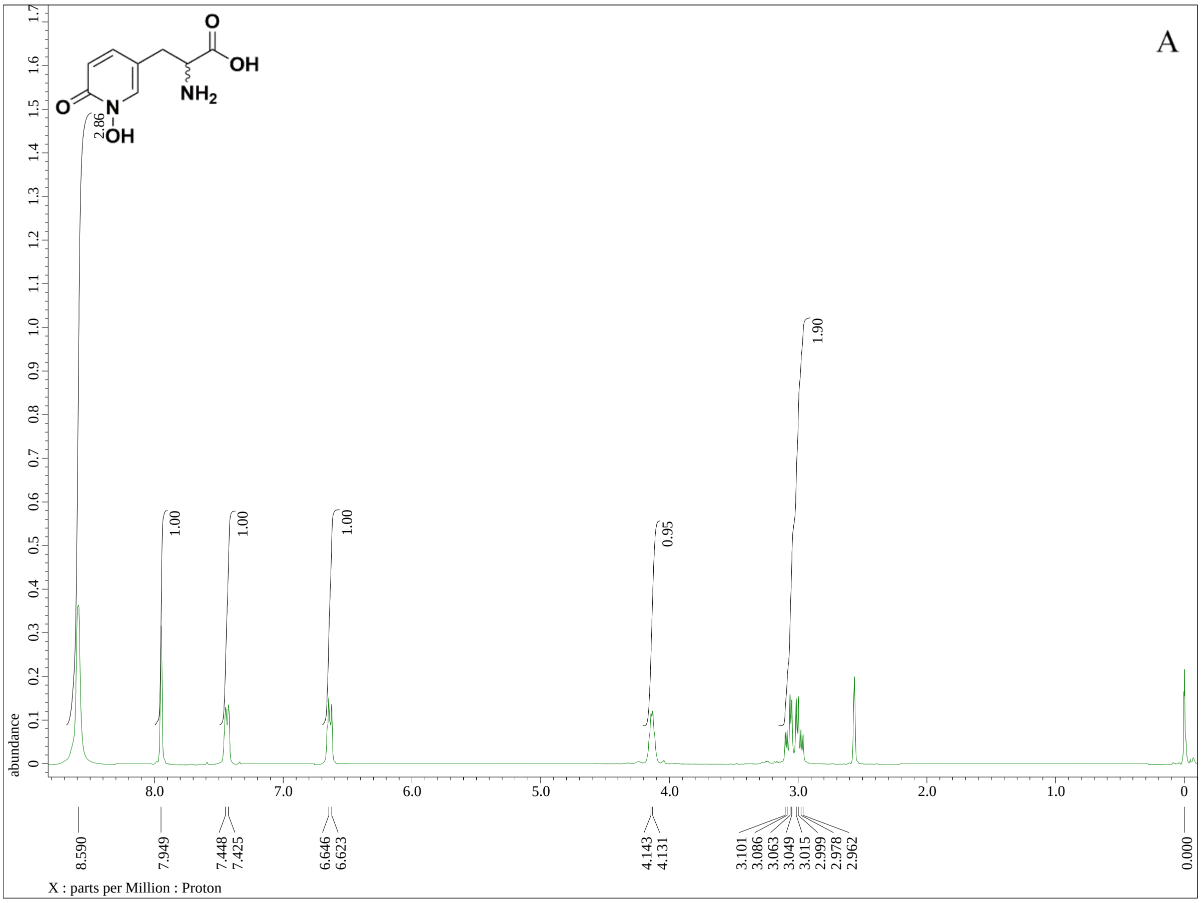
A
NH2
OH
O
N
OH
2.86
1.00
1.00
1.00
0.95
1.90
abundance
8.590
7.949
7.448
7.425
6.646
6.623
4.143
4.131
3.101
3.086
3.063
3.049
3.015
2.999
2.978
2.962
0.000
X : parts per Million : Proton

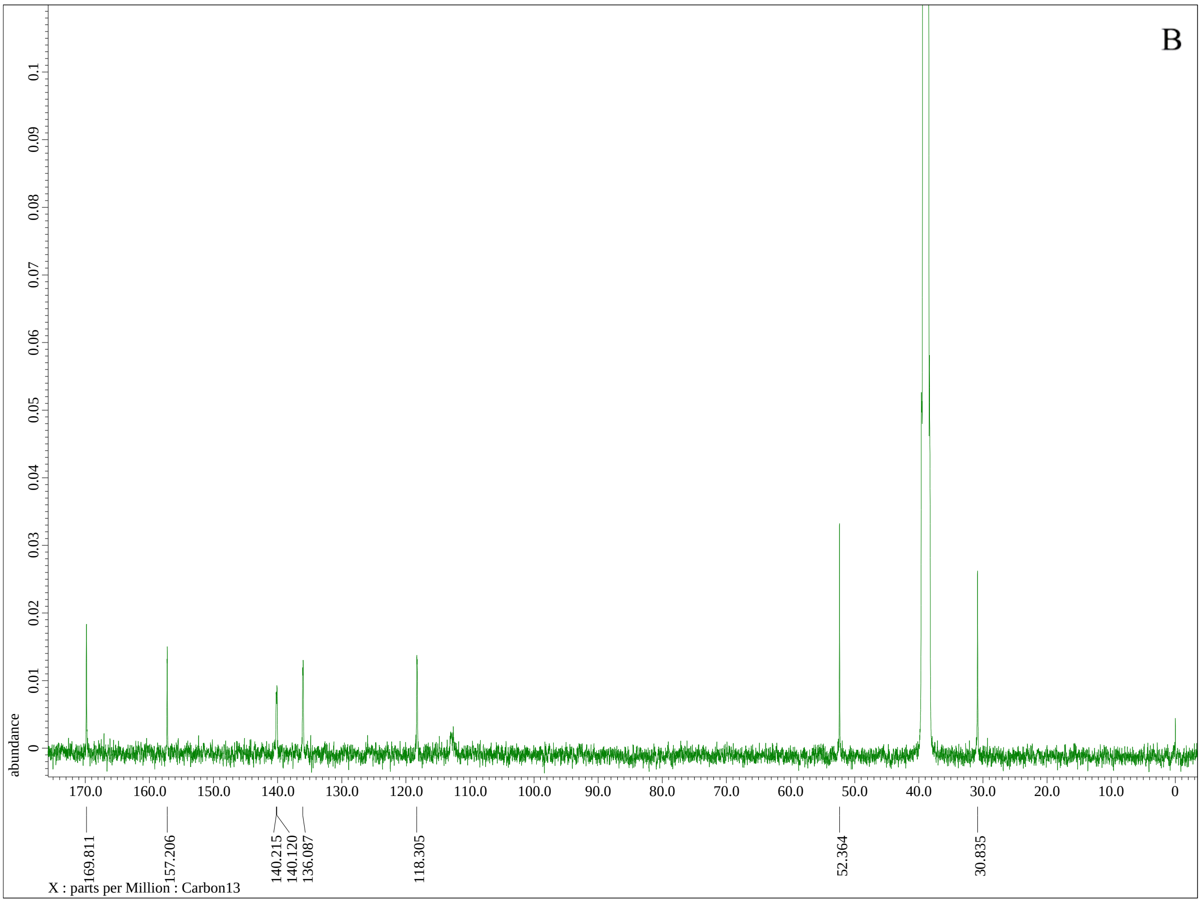
B
abundance
169.811
157.206
140.215
140.120
136.087
118.305
52.364
30.835
X : parts per Million : Carbon13

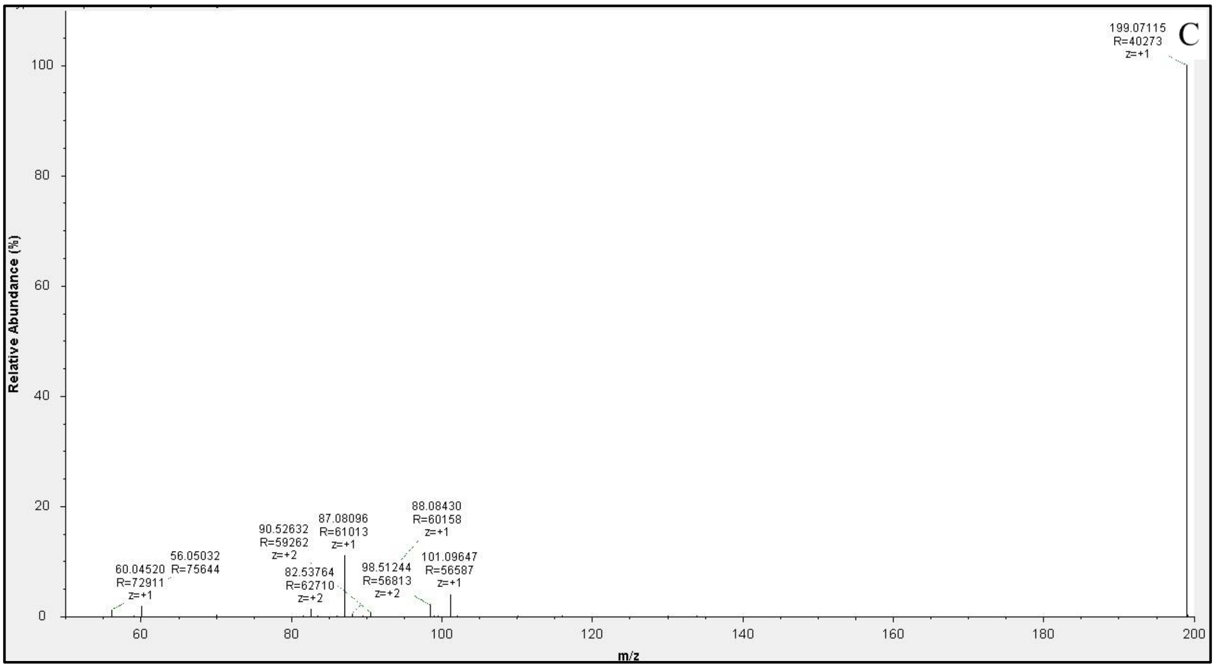

Figure S17: ^{1}H-NMR spectra of *rac*-2-amino-3-(1-hydroxy-6-oxo-1,6-dihydropyridin-3-yl)propanoic acid (18) at 400 MHz in DMSO-d_6 and (**B**)^{13}C-NMR spectra of *rac*-2-amino-3-(1-hydroxy-6-oxo-1,6-dihydropyridin-3-yl)propanoic acid (18) at 100 MHz in DMSO-d_6 and (**C**) HRMS of *rac*-2-amino-3-(1-hydroxy-6-oxo-1,6-dihydropyridin-3-yl)propanoic acid (18)

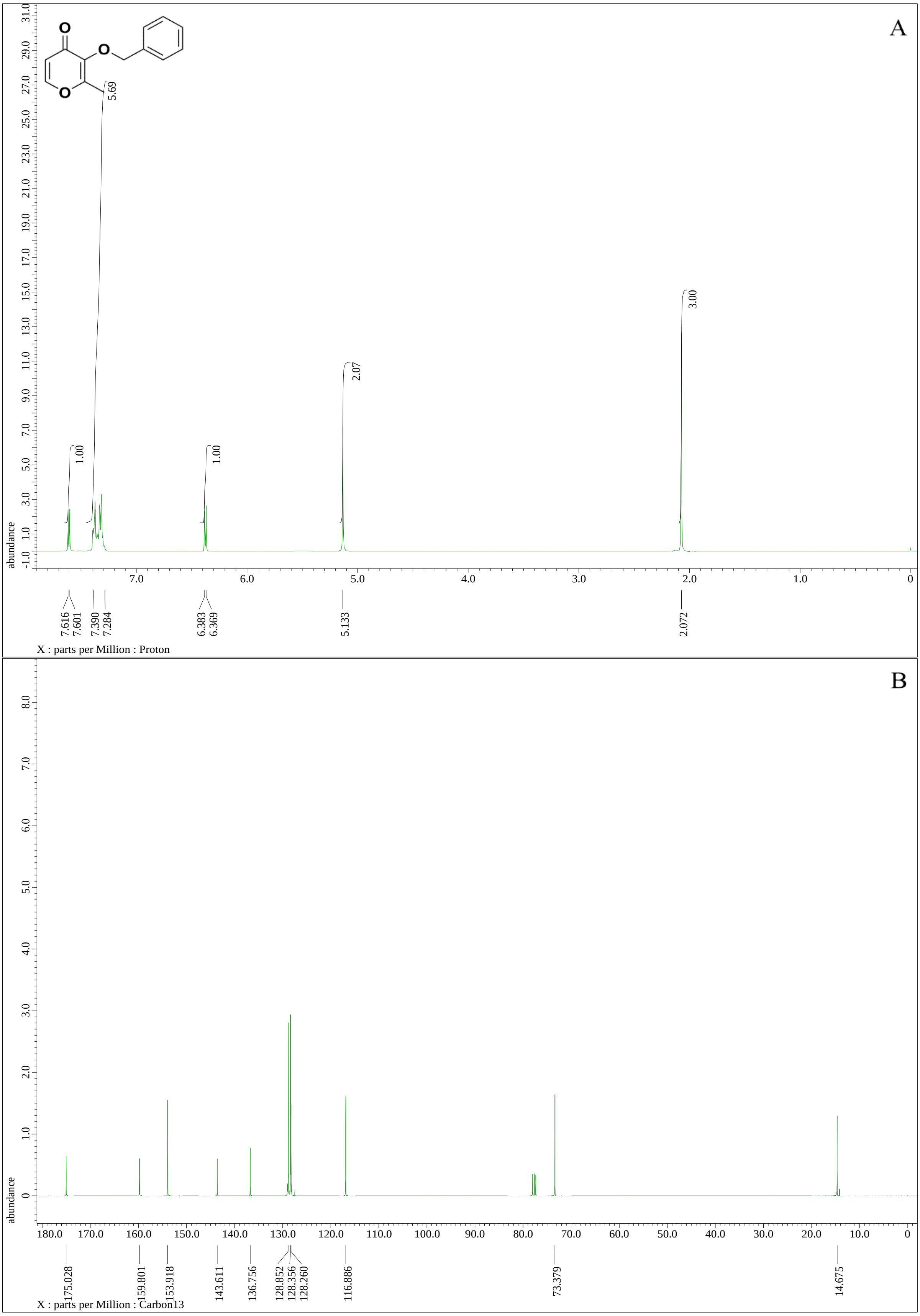

Figure S18: (A) ^{1}H-NMR spectra of 3-(benzyloxy)-2-methyl-1,4-dihydropyridin-4-one (19) at 400 MHz in $CDCl_3$, (B)^{13}C-NMR spectra of 3-(benzyloxy)-2-methyl-1,4-dihydropyridin-4-one (19) at 100 MHz in $CDCl_3$

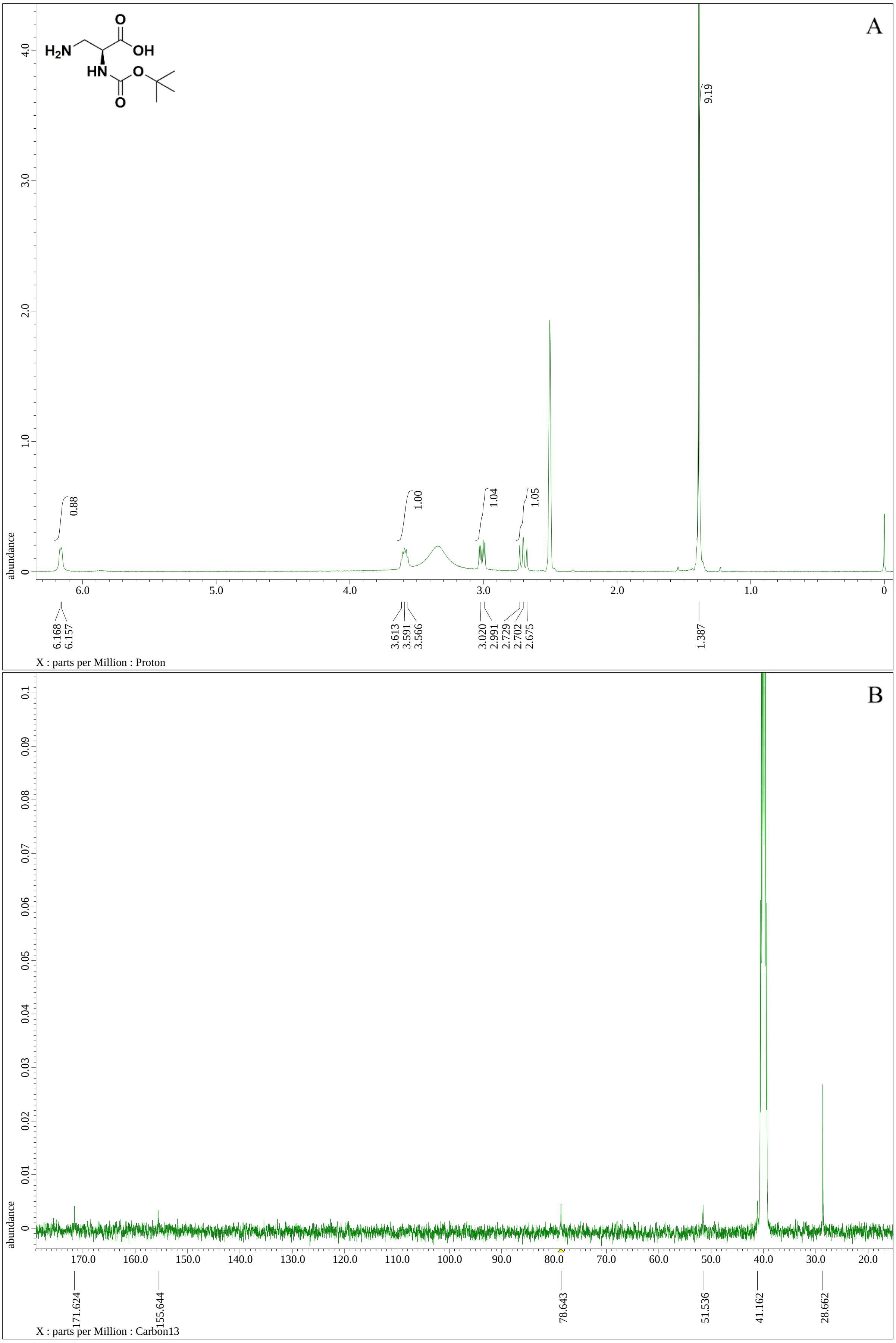

Figure S19: (A) ^{1}H-NMR spectra of (2L)-3-amino-2-{[(tert-butoxy)carbonyl]amino}propanoic acid (20) at 400 MHz in DMSO-*d₆* and (B)^{13}C-NMR spectra of (2L)-3-amino-2-{[(tert-butoxy)carbonyl]amino}propanoic acid (20) at 100 MHz in DMSO-*d₆*.

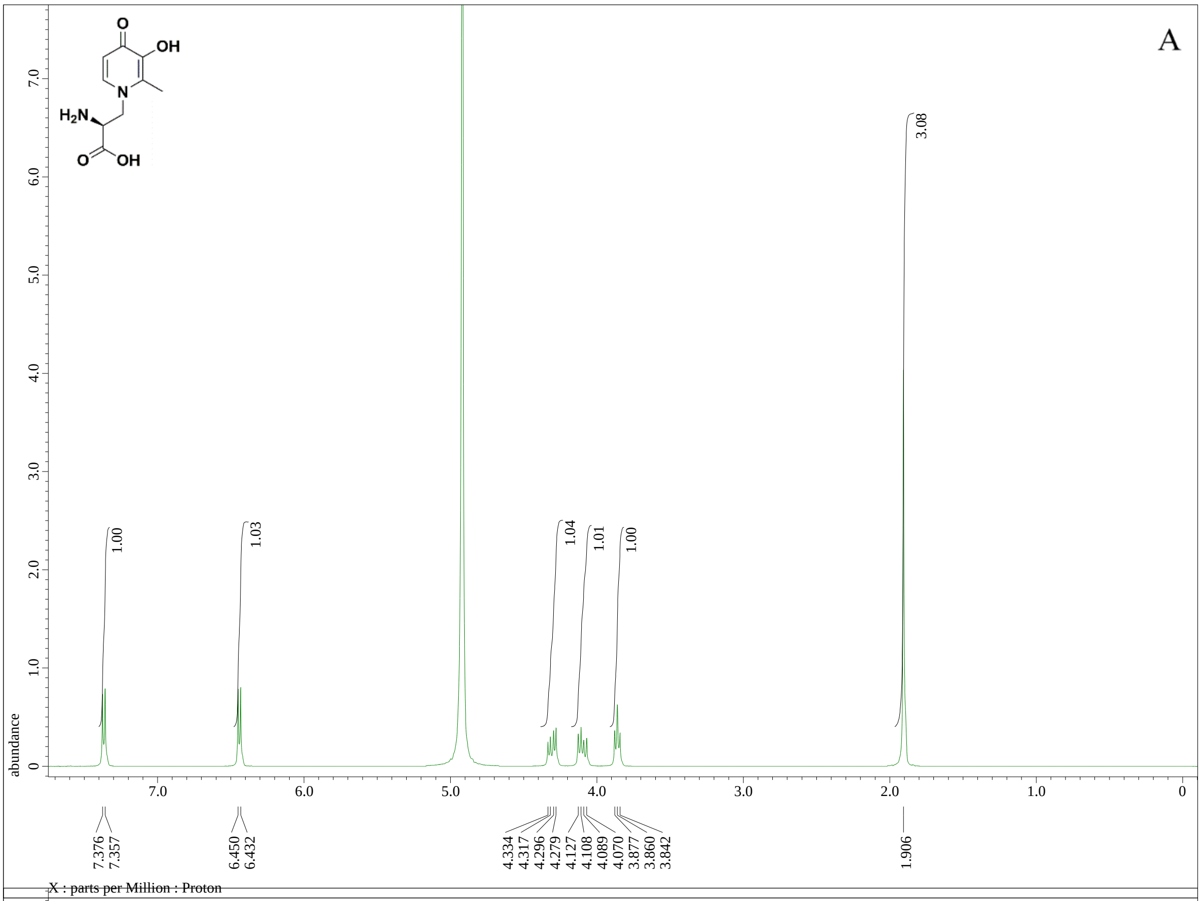
A
O
OH
N
H2N
O
OH
abundance
1.00
1.03
1.04
1.01
1.00
3.08
7.376
7.357
6.450
6.432
4.334
4.317
4.296
4.279
4.127
4.108
4.089
4.070
3.877
3.860
3.842
1.906
X : parts per Million : Proton

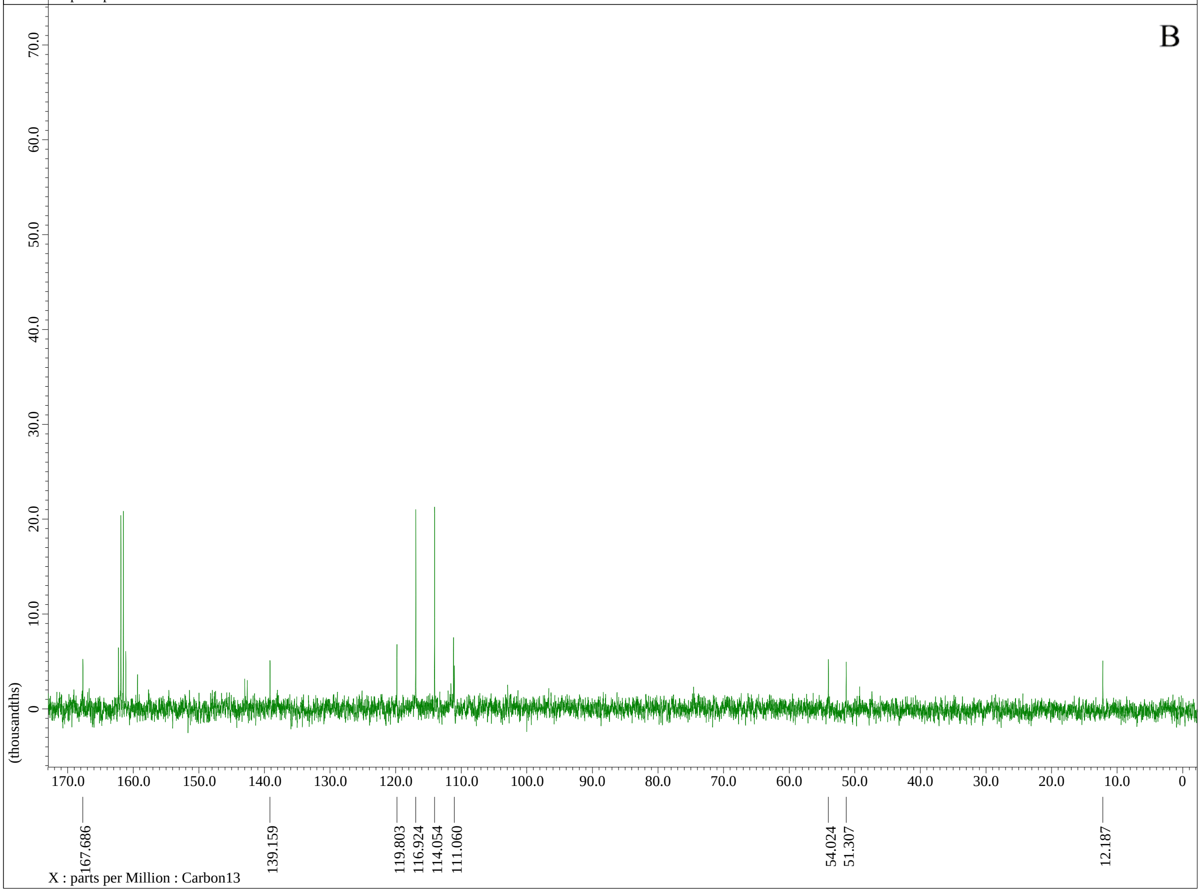
B
(thousandths)
167.686
139.159
119.803
116.924
114.054
111.060
54.024
51.307
12.187
X : parts per Million : Carbon13

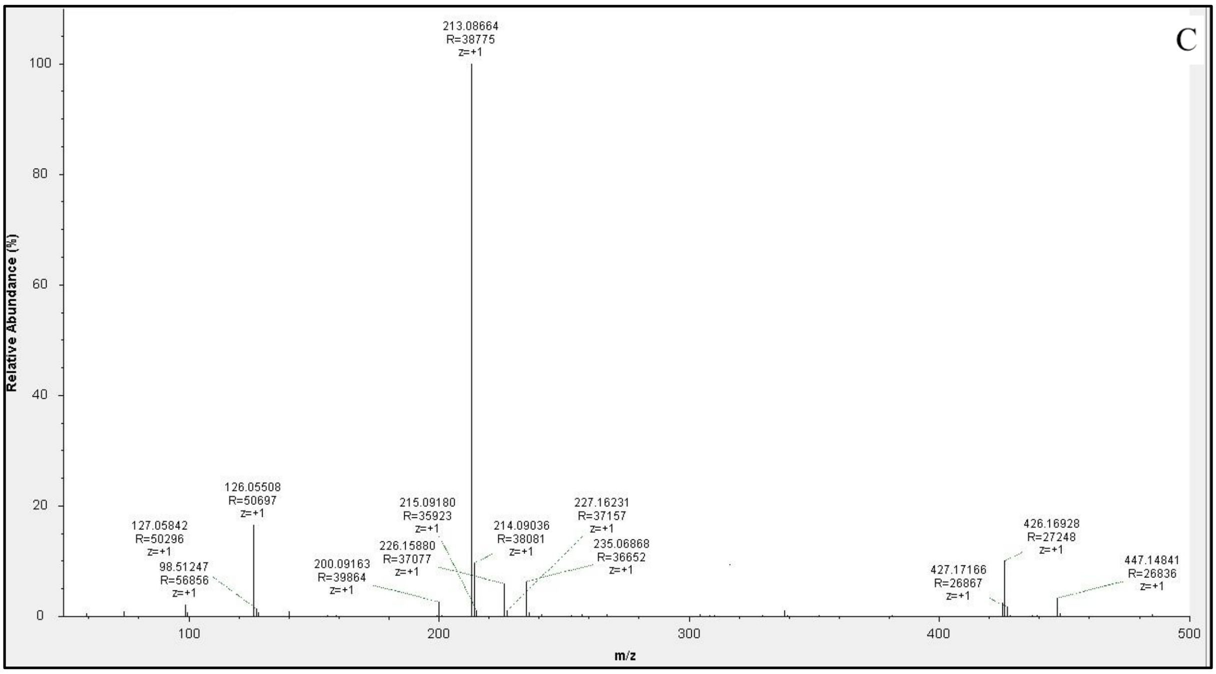

Figure S10: (A) ^{1}H-NMR spectra (2L)-2-amino-3-(3-hydroxy-2-methyl-4-oxo-1,4-dihydropyridin-1-yl)propanoic acid (22) at 400 MHz in D_2O/CF_3CO_2D and (B)^{13}C-NMR spectra of (2L)-2-amino-3-(3-hydroxy-2-methyl-4-oxo-1,4-dihydropyridin-1-yl)propanoic acid (22) at 100 MHz in D_2O/CF_3CO_2D and (C) HRMS of (2L)-2-amino-3-(3-hydroxy-2-methyl-4-oxo-1,4-dihydropyridin-1-yl)propanoic acid (22).

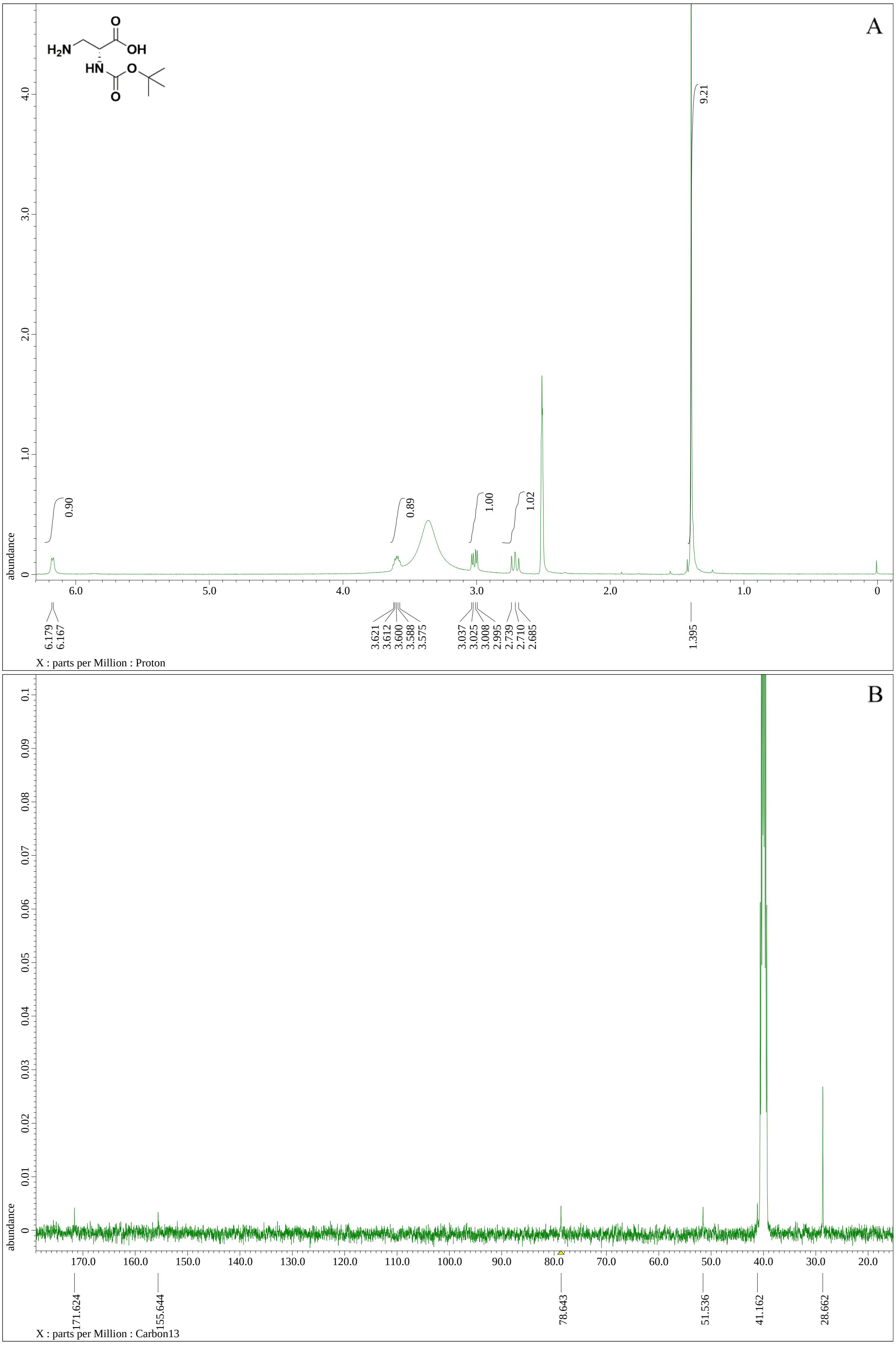

Figure S21: (A) ^{1}H-NMR spectra of (2D)-3-amino-2-{[(tert-butoxy)carbonyl]amino}propanoic acid (21) at 400 MHz in
DMSO-d6 and (B) ^{13}C-NMR spectra of (2D)-3-amino-2-{[(tert-butoxy)carbonyl]amino}propanoic acid

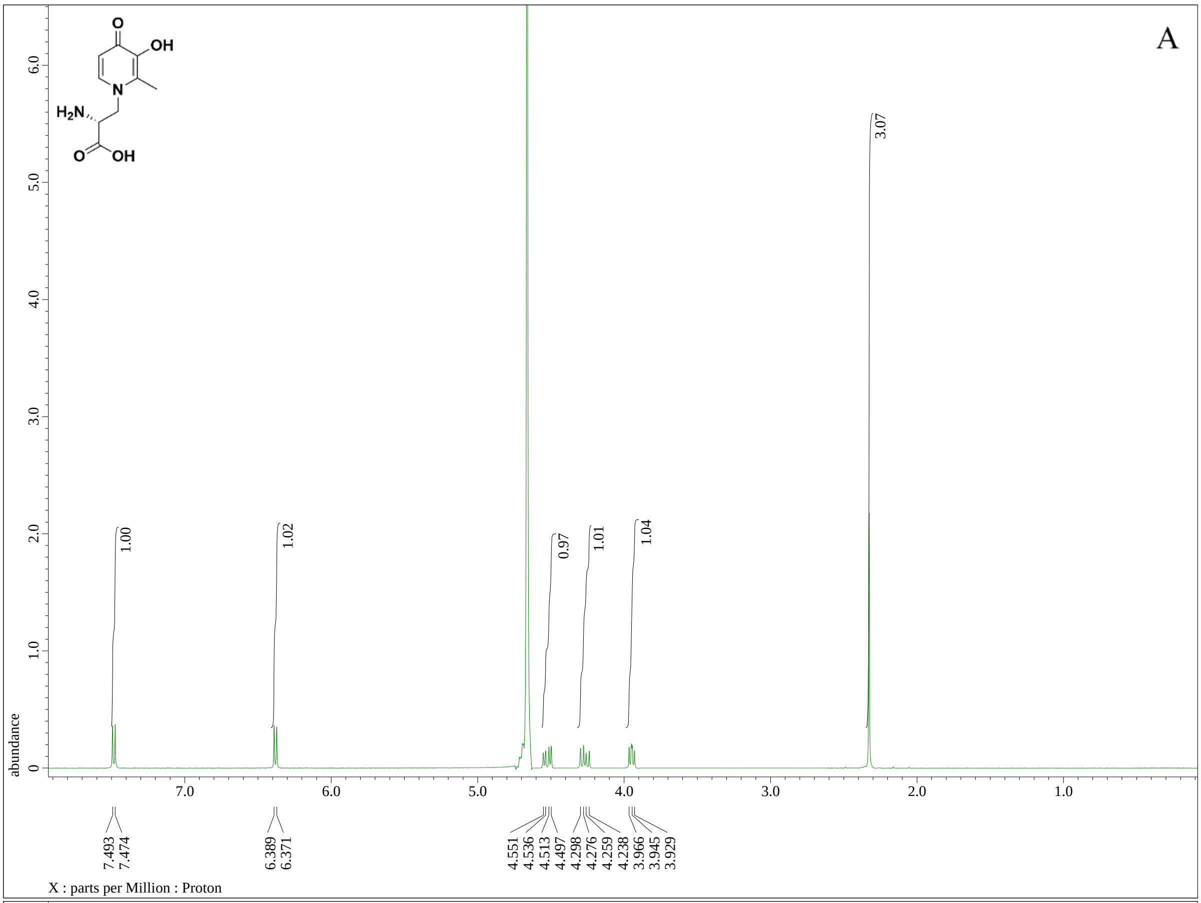
A
OH
O
N
H2N
OH
O
abundance
1.00
1.02
0.97
1.01
1.04
3.07
7.493
7.474
6.389
6.371
4.551
4.536
4.513
4.497
4.298
4.276
4.259
4.238
3.966
3.945
3.929
X : parts per Million : Proton

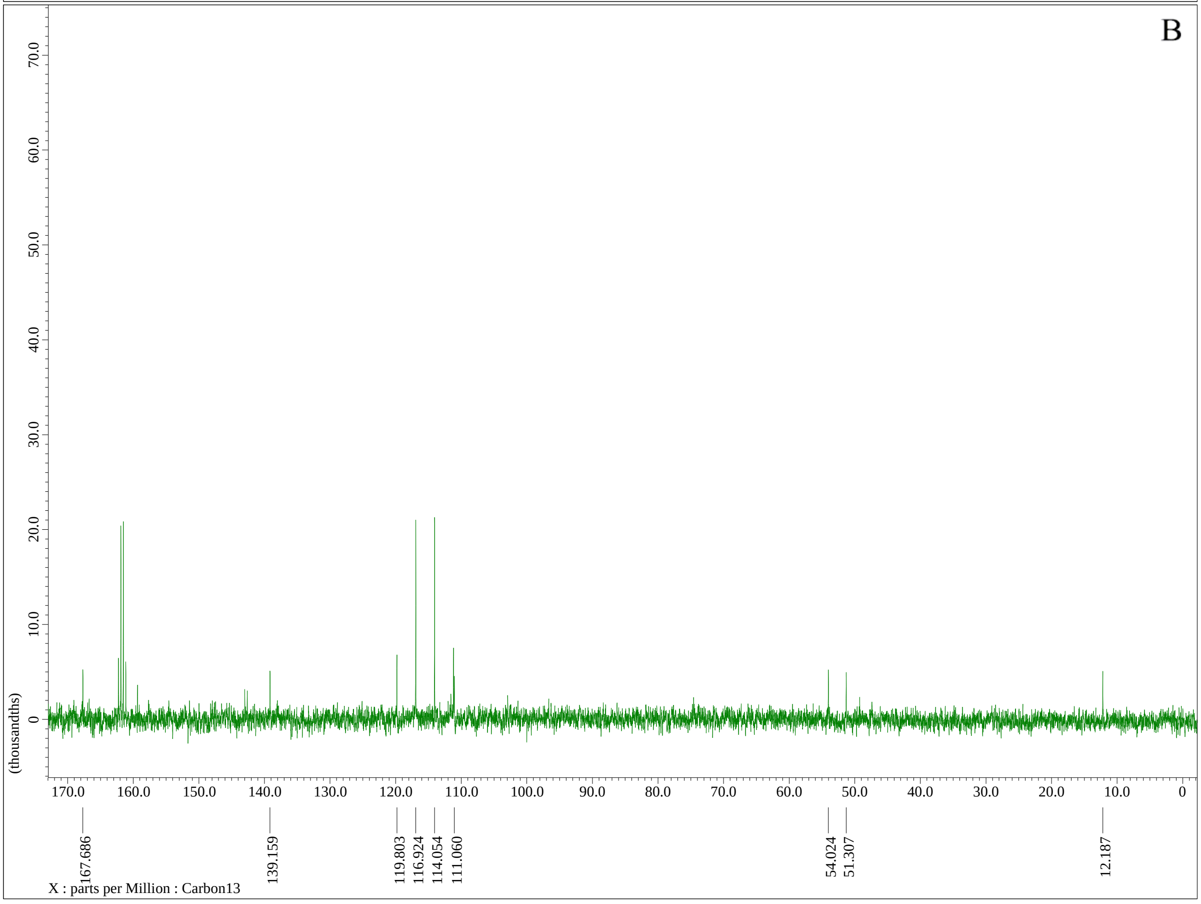
B
(thousandths)
167.686
139.159
119.803
116.924
114.054
111.060
54.024
51.307
12.187
X : parts per Million : Carbon13

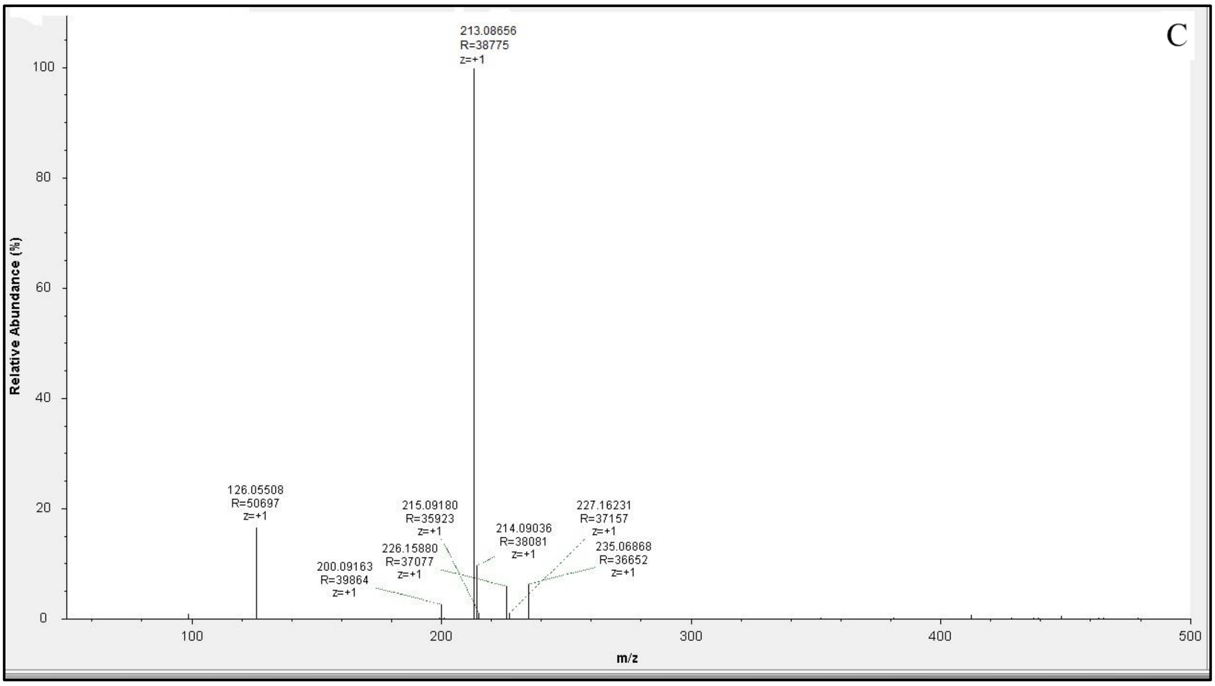

Figure S22: (A) ^{1}H-NMR spectra (2D)-2-amino-3-(3-hydroxy-2-methyl-4-oxo-1,4-dihydropyridin-1-yl)propanoic acid (23) at 400 MHz in D_2O/CF_3CO_2D and (B)^{13}C-NMR spectra of (2D)-2-amino-3-(3-hydroxy-2-methyl-4-oxo-1,4-dihydropyridin-1-yl)propanoic acid (23) at 100 MHz in D_2O/CF_3CO_2D and (C) HRMS of (2R)-2-amino-3-(3-hydroxy-2-methyl-4-oxo-1,4-dihydropyridin-1-yl)propanoic acid (23).

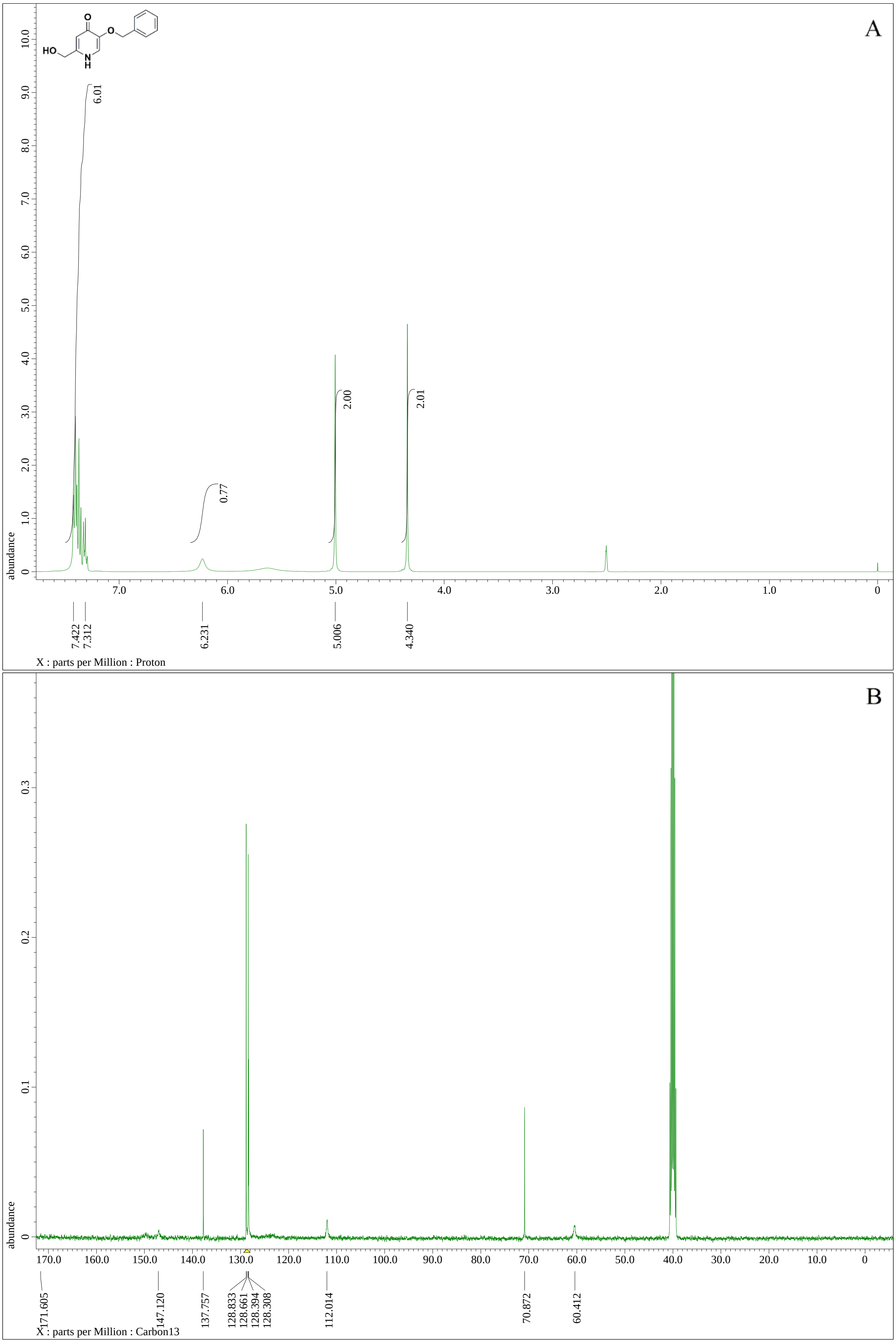

Figure S23: (A) ^{1}H-NMR spectra of 5-(benzyloxy)-2-(hydroxymethyl)-1,4-dihydropyridin-4-one (24) at 400 MHz in DMSO-*d₆* and (B)^{13}C-NMR spectra of 5-(benzyloxy)-2-(hydroxymethyl)-1,4-dihydropyridin-4-one (24) at 100 MHz in DMSO-*d₆*.

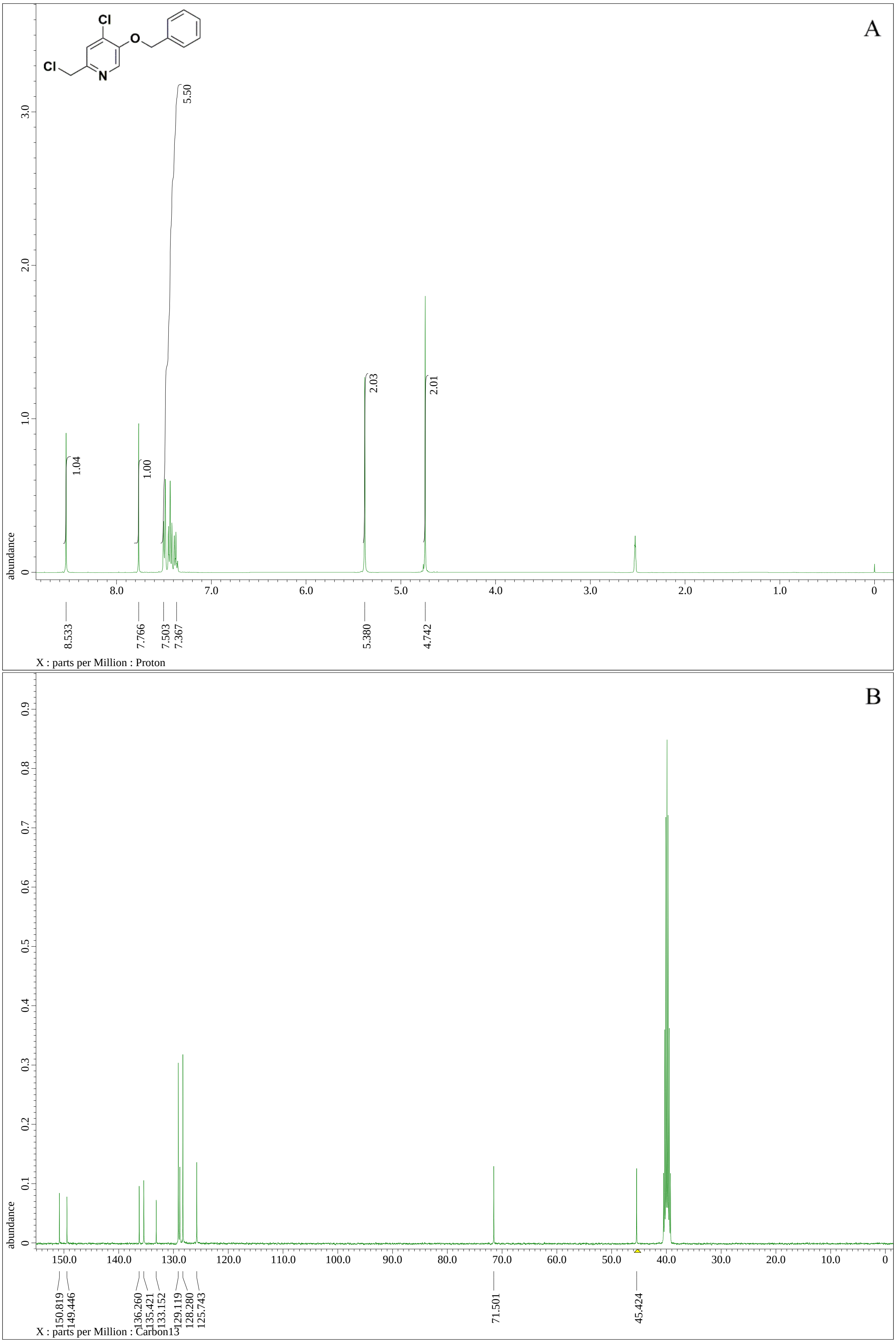

Figure S24: (A) ^{1}H-NMR spectra of 5-(benzyloxy)-4-chloro-2-(chloromethyl)pyridine (25) at 400 MHz in DMSO-d_6 and (B)^{13}C-NMR spectra of 5-(benzyloxy)-4-chloro-2-(chloromethyl)pyridine (25) at 100 MHz in DMSO-d_6.

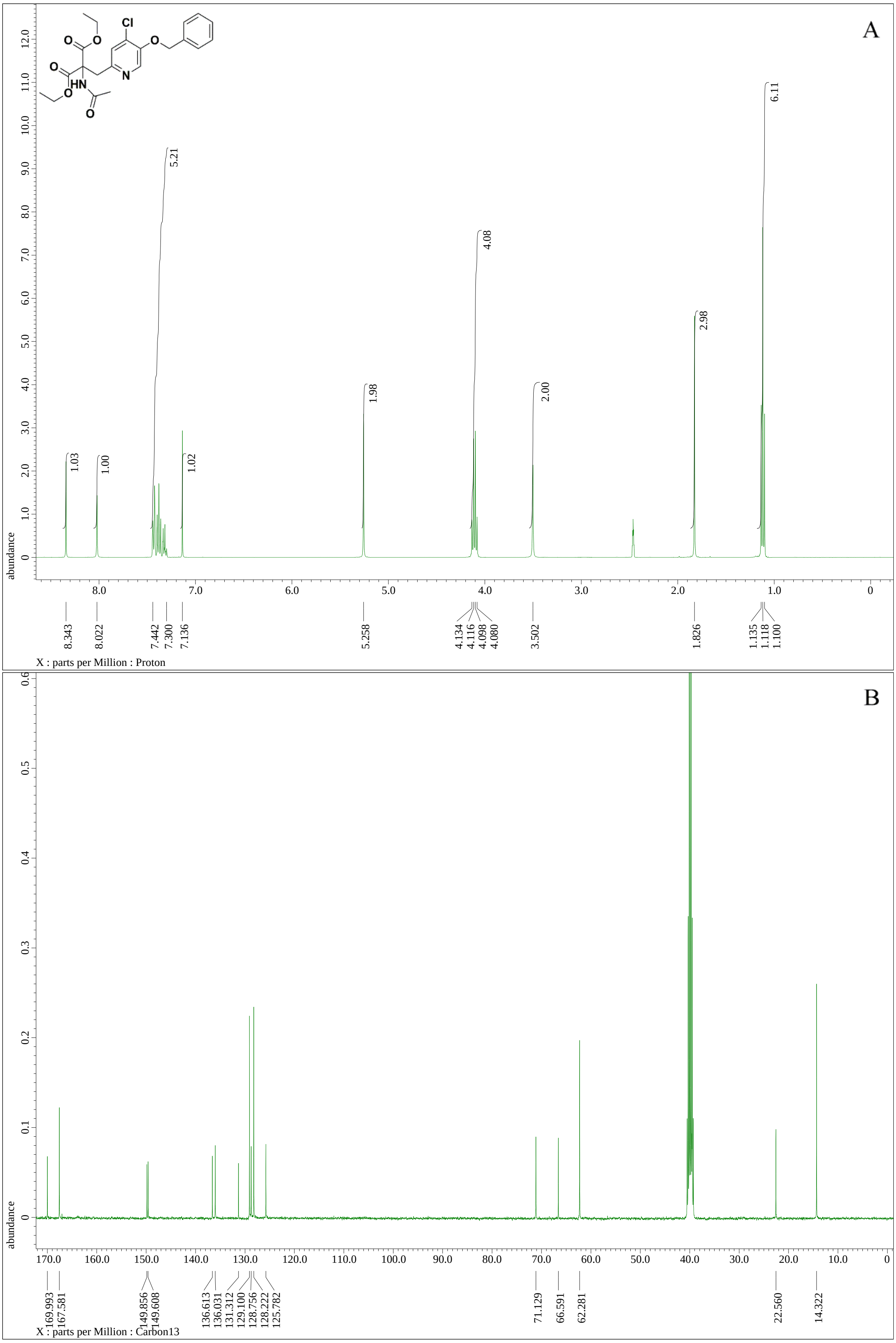

Figure S25: (A) 1H-NMR spectra of 1,3-diethyl 2-{[5-(benzyloxy)-4-chloropyridin-2-yl]methyl}-2-acetamidopropanedioate (26) at 400 MHz in DMSO-d6 and (B)13C-NMR spectra of 1,3-diethyl 2-{[5-(benzyloxy)-4-chloropyridin-2-yl]methyl}-2-acetamidopropanedioate (26) at 100 MHz in DMSO-d6.

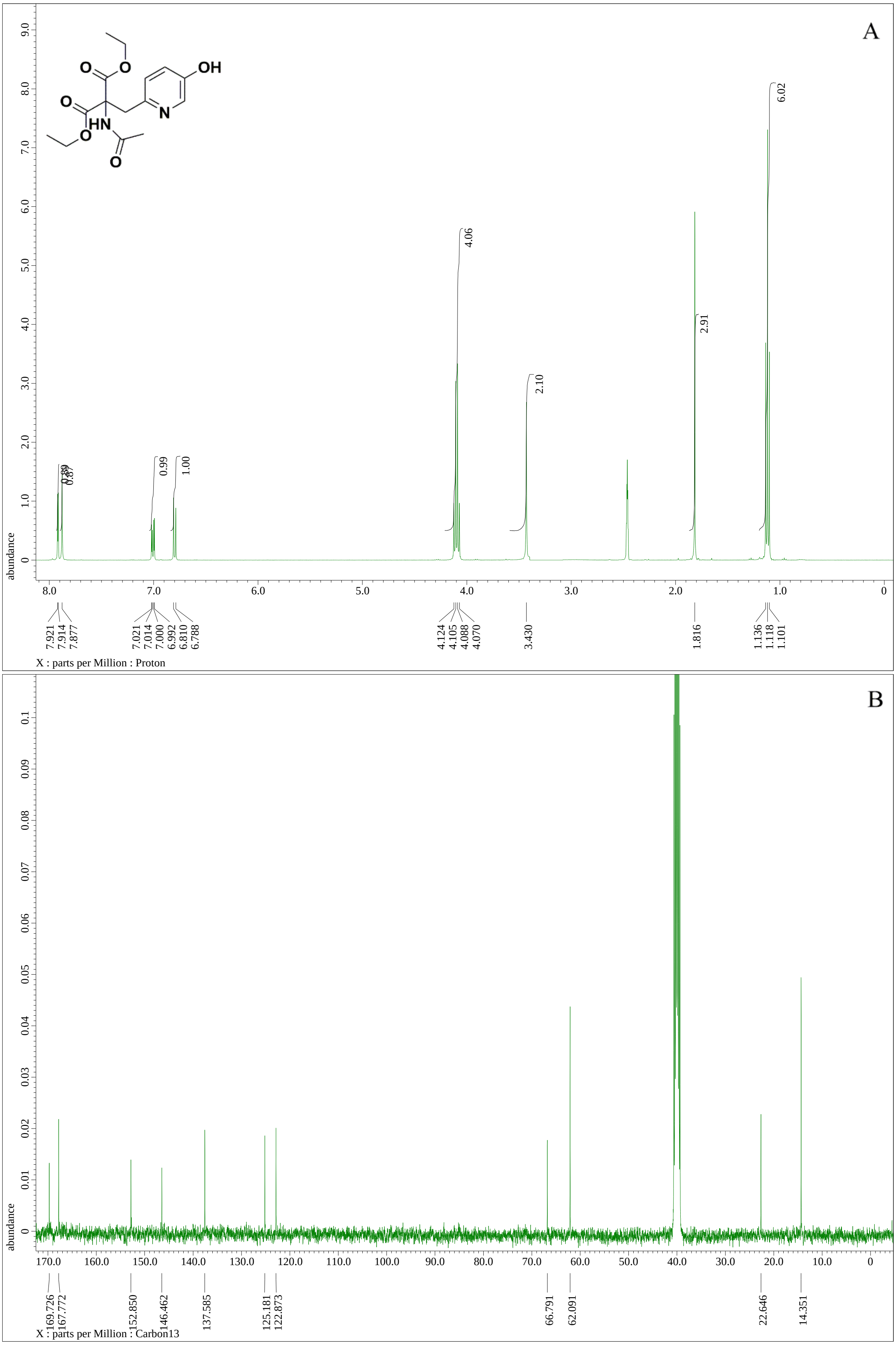

Figure S26: (A) ^{1}H-NMR spectra of 1,3-diethyl 2-acetamido-2-[(5-hydroxypyridin-2-yl)methyl]propanedioate (27) at 400 MHz in DMSO-d_6 and (B)^{13}C-NMR spectra of 1,3-diethyl 2-acetamido-2-[(5-hydroxypyridin-2-yl)methyl]propanedioate (27) at 100 MHz in DMSO-d_6.

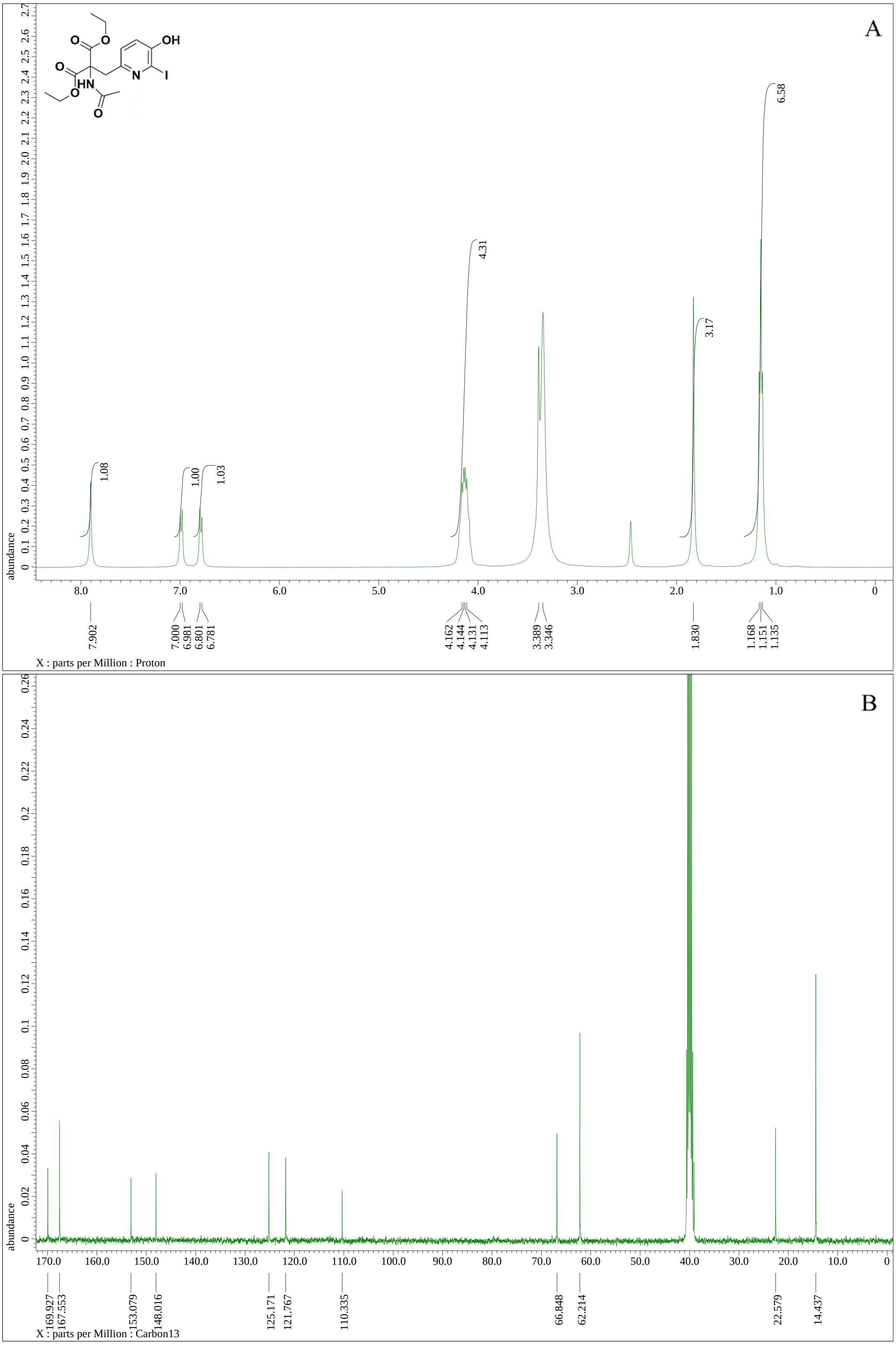

Figure S27: (A) ^{1}H-NMR spectra of 1,3-diethyl 2-acetamido-2-[(5-hydroxy-6-iodopyridin-2-yl)methyl]propanedioate (28) at 400 MHz in DMSO-d_6 and (B)^{13}C-NMR spectra of 1,3-diethyl 2-acetamido-2-[(5-hydroxy-6-iodopyridin-2-yl)methyl]propanedioate (28) at 100 MHz in DMSO-d_6

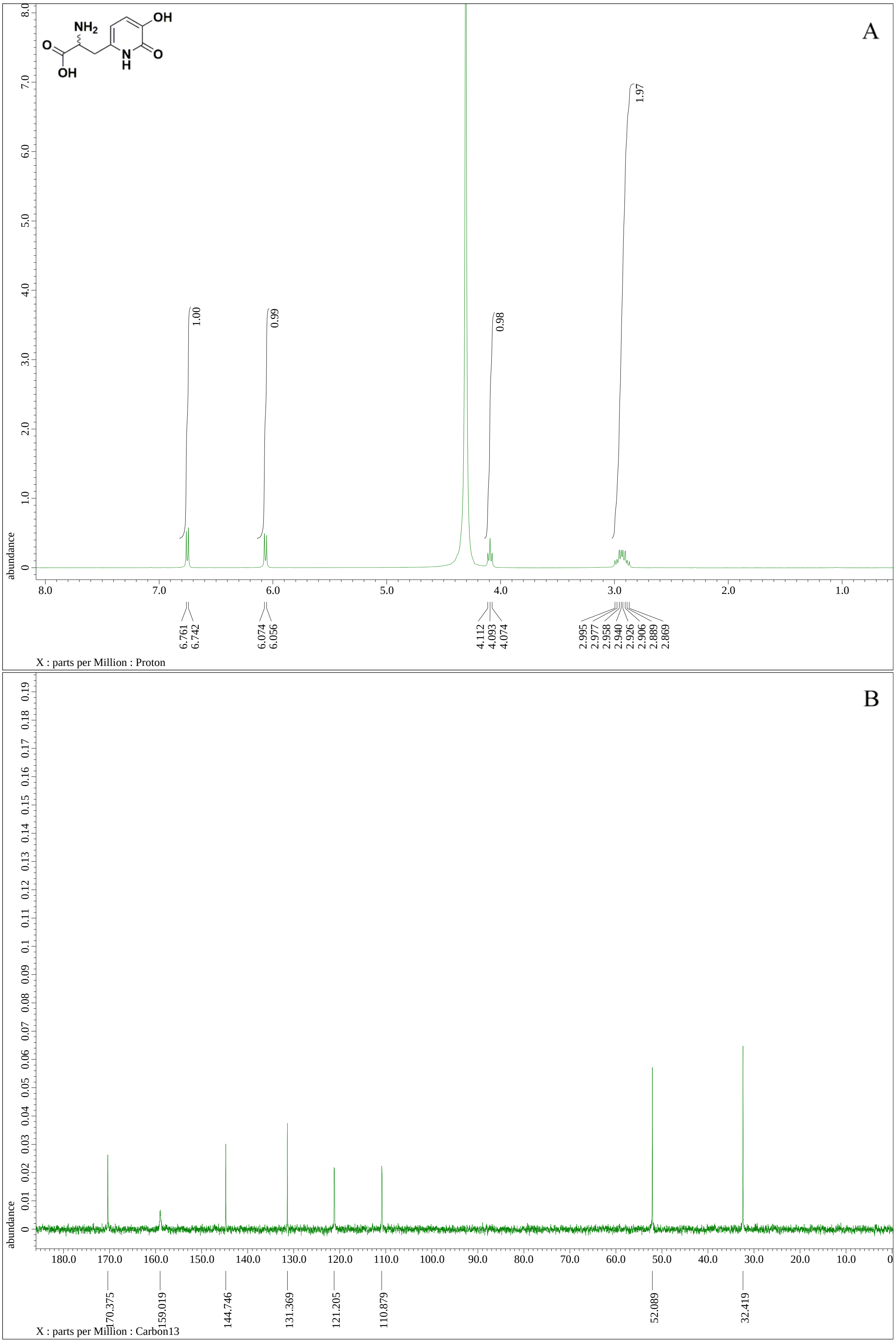

A
NH2
OH
O
OH
N
H
O
abundance
1.00
0.99
0.98
1.97
6.761
6.742
6.074
6.056
4.112
4.093
4.074
2.995
2.977
2.958
2.940
2.926
2.906
2.889
2.869
X : parts per Million : Proton
B
abundance
170.375
159.019
144.746
131.369
121.205
110.879
52.089
32.419
X : parts per Million : Carbon13

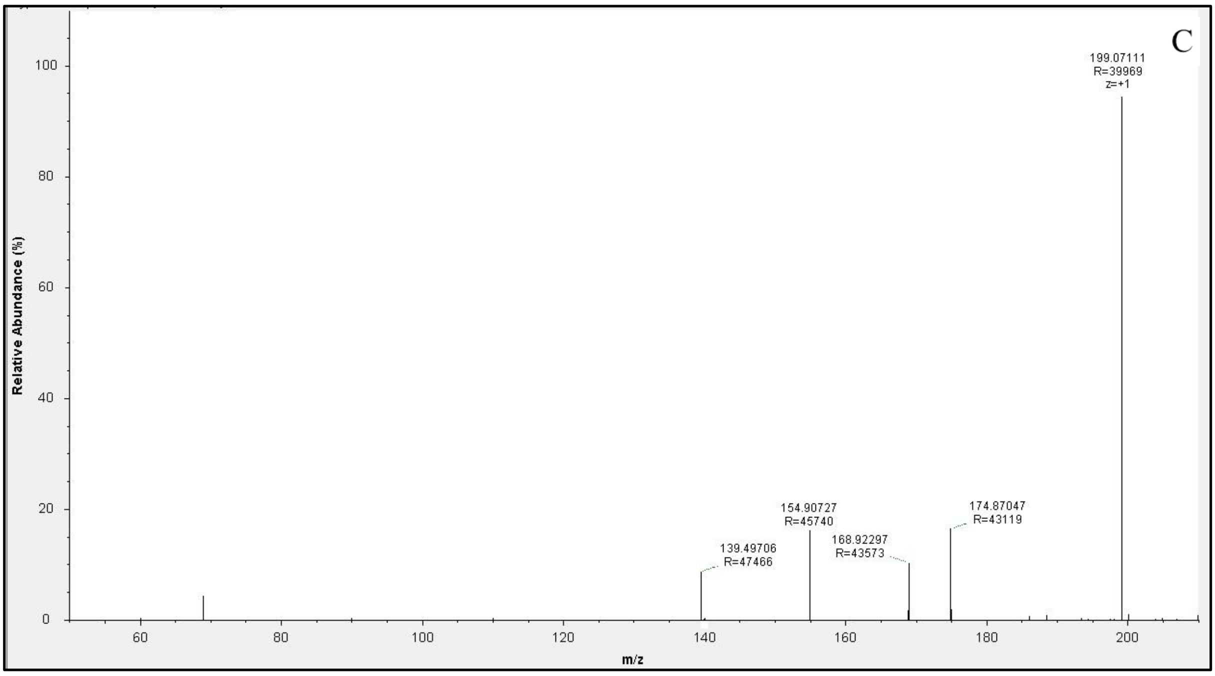

Figure S28: (A) ^{1}H-NMR spectra of rac-2-amino-3-(5-hydroxy-6-oxo-1,6-dihydropyridin-2-yl)propanoic acid (29) at 400 MHz in D_2O/CF_3CO_2D, (B)^{13}C-NMR spectra of rac-2-amino-3-(5-hydroxy-6-oxo-1,6-dihydropyridin-2-yl)propanoic acid (29) at 100 MHz in D_2O/CF_3CO_2D and (C) HRMS of rac-2-amino-3-(5-hydroxy-6-oxo-1,6-dihydropyridin-2-yl)propanoic acid (29).

REFERENCES

1. Harris RLN (1976) Potential Wool Growth Inhibitors Improved Syntheses of Mimosine and Related 4(1H)-Pyridones. Aust J Chem 29(6): 1329-1334.

2. Thomas FA (1962) The Oxidation of the Side Chain in Kojic Acid Benzyl Ether. J Chem Soc: 439-441.

3. Struk Ł, Sośnicki JG (2012) Noncryogenic Synthesis of Functionalized 2-Methoxypyridines by Halogen-Magnesium Exchange Using Lithium dibutyl(isopropyl)magnesate(1-) and Lithium Chloride. Synthesis (Stuttg) 44 (5): 735-746.

4. Liu S, Shang R, Shi L, Wan DCC, Lin H (2014) Synthesis and Biological Evaluation of 7H-thiazolo[3,2-B]-1,2,4-Triazin-7-One Derivatives as Dual Binding Site Acetylcholinesterase Inhibitors. Eur J Med Chem 81: 237-244.

5. Stojkovic MA, Piotrowski P, Schmuck C, Piantanida I (2015) A Short, Rigid Linker between Pyrene and Guanidiniocarbonyl-Pyrrole Induced a New Set of Spectroscopic Responses to the ds-DNA Secondary Structure. Org Biomol Chem 13(6): 1629-1633.

6. Harris RLN, Teitei T (1977) Potential Wool Growth Inhibitors. 2(1H)-Pyridone Analogues of Mimosine. Aust J Chem 30 (3): 649-655.

7. Liu ZD, Hider CR (2002) Design of Iron Chelators with Therapeutic Application. Coord Chem Rev 232: 151-171.
